# ﻿Emendation of morphology and infrageneric standards of *Parasola* (*Psathyrellaceae*, *Agaricales*) and its species diversity in China

**DOI:** 10.3897/imafungus.16.143796

**Published:** 2025-05-30

**Authors:** LiYang Zhu, Tolgor Bau

**Affiliations:** 1 Engineering Research Center of Chinese Ministry of Education for Edible and Medicinal Fungi, Jilin Agricultural University, Changchun, Jilin 130118, China Jilin Agricultural University Changchun China; 2 Key Laboratory of Edible Fungi Resources and Utilization (North), Ministry of Agriculture, Changchun, Jilin 130118, China Key Laboratory of Edible Fungi Resources and Utilization (North), Ministry of Agriculture Changchun China

**Keywords:** Coprinoid fungi, evolution, morphology, phylogeny, taxonomy

## Abstract

The genus *Parasola*, a significant lineage of coprinoid fungi, represents a basal clade within the family *Psathyrellaceae*, with species saprotrophic on soil, wood, or occasionally on dungs. While the infrageneric classification of Parasolahas been established into two sections,sect.Conopileae and sect.Parasola, based on phylogenetic studies, the corresponding morphological differentiation criteria remain poorly defined, and the species diversity of *Parasola* in China is poorly understood, with only eight known species previously recorded. Through extensive fieldwork across ten provinces in China, this study discovered eight proposed new species and three new records to the country, each accompanied by detailed description and line drawings. A molecular phylogenetic analysis, incorporating samples from China and other species confirmed by previous studies, was performed using multiple loci, including the internal transcribed spacer regions (ITS), the large subunit nuclear ribosomal RNA (nrLSU), the translation elongation factor EF-1 alpha gene (tef1-α), and the beta-tubulin gene (β-tublin), and integrated with morphological features and geographic data. The results confirmed the current infrageneric classification of *Parasola* into two sections and established morphological differentiation criteria: (1) species in sect.Conopileae exhibit psathyrelloid basidiomata, while those in sect.Parasola display parasoloid basidiomata; (2) the formation of pileus plication due to the growth of secondary pileipellis at maturity distinguishes sect.Parasola, whereas its absence characterizes sect.Conopileae; and (3) the two sections differ in lamellae-stipe attachment types, influenced by the arrangement of caulocystidia at stipe’s upper part— adjacent in sect.Conopileae without accumulated caulocystidia, and free in sect.Parasola with enlongated stipepellis hyphae bearing terminal cystidia. Notably, the presence or absence of sclerocystidia, a previously used classification criterion, no longer aligns with monophyletic grouping system of this genus. The study also explores the formation mechanism of the pileus plication and the free-type lamellae-stipe attachment in *Parasola*, highlighting evolutionary trends such as the emergence of secondary pileipellis, the increased distance between the stipe and gills, loss of sclerocystidia, transition from monomorphic to dimorphic basidia, the flattening and polygonalization of basidiospores, and the partialization of germ pores, all mapped onto phylogenetic framework. A taxonomic key to the species of Parasola, validated by phylogenetic results, is provided, enhancing our understanding of the morphological and phylogenetic diversity of *Parasola* and offering new insights into its infrageneric classification and evolutionary path.

## ﻿Introduction

Coprinoid fungi, an enigmatic group of agarics, may harbor numerous yet-undiscovered species and ecological mysteries. Among these, the genus *Parasola* Redhead, Vilgalys & Hopple, belonging to the family *Psathyrellaceae* Vilgalys, Moncalvo & Redhead, comprises small to medium-sized coprinoid fungi characterized by the absence of squamose pilei. These fungi are widely distributed across nearly all grassland or forest ecosystems globally, as documented by the Global Biodiversity Information Facility (GBIF, https://www.gbif.org) and numerous literature records (Redhead et al. 2001; Nagy et al. 2009; Szarkándi et al. 2017; Wächter and Melzer 2020; Schafer et al. 2022). Species within this genus are saprotrophic, typically colonizing clayish soil or humus layer, moreover, with some also found growing on dungs of herbivorous animals (Uljé and Bas 1988; Uljé 2005; Nagy et al. 2009).

To date, *Parasola* species have been reported across multiple continents, including Europe (Uljé and Bas 1988; Nagy et al. 2009; Schafer 2014; Szarkándi et al. 2017; Vote 2020; Pošta et al. 2023), Asia (Pegler 1986; Chi and Bau 2013; Hussain et al. 2016; Hussain et al. 2017; Cho et al. 2018; Hussain et al. 2018; Ganga and Manimohan 2019; Huang 2019; Zhu et al. 2022; Khan et al. 2023), North America (Smith and Hesler 1946; Pegler 1983; Pérez-Silva 2018), Africa (Pegler 1968) and Oceania (Keirle 2004; Hubregtse 2019; Bougher 2020). According to Index Fungorum (http://www.indexfungorum.org, accessed on 30 Aug. 2024), 40 valid species names are currently recorded. However, the actual number of species might be lower due to taxonomic revisions. For instance, *Parasolagalericuliformis* (Losa ex Watling) Redhead, Vilgalys & Hopple and *Parasolaleiocephala* (P.D. Orton) Redhead, Vilgalys & Hopple have been demonstrated to be synonymous with *Parasolalactea* (A.H. Sm.) Redhead, Vilgalys & Hopple. Additionally, *Parasolahemerobia* (Fr.) Redhead, Vilgalys & Hopple is likely conspecific with *Parasolaplicatilis* (Curtis) Redhead, Vilgalys & Hopple, as supported by morphological observation of type specimens and a series of phylogenetic studies (Nagy et al. 2009; Nagy et al. 2010a; Szarkándi et al. 2017; Hussain et al. 2018; Wächter and Melzer 2020). Nevertheless, the taxonomic status of several species remains contentious due to the lack of robust molecular evidence.

The genus *Parasola* is distinguished from other coprinoid fungi by the absence of a conspicuous veil and the nearly non-deliquescent or just weakly deliquescent nature of the mature basidiomata. Additionally, all species within this genus possess a hymeniform pileipillis composed of sphaeropedunculate or clavate cells arranged in palisade-like pattern (Redhead et al. 2001; Uljé 2005; Schafer 2010, 2014; Szarkándi et al. 2017; Wächter and Melzer 2020). Key morphological characteristics used for species delimitation and identification within *Parasola* include the color and size of basidiomata, the presence or absence of a plicate pileus, the shape and size of basidiospores, the central or eccentric position of the germ pore, and presence or absence of sclerocystidia on pileipellis. In some cases, the shape and size of cheilocystidia and pleurocystidia are also considered (Nagy et al. 2009; Nagy et al. 2010a; Schafer 2010; Hussain et al. 2016a; Hussain et al. 2017; Szarkándi et al. 2017). Despite these diagnostic features, species within *Parasola* are frequently misidentified due to their macro-morphological similarities with each other or with species in *Tulosesus* D. Wächt. & A. Melzer.

*Parasola* was limited to species characterized by deeply grooved pileus for a long time. Lange (1915) established sect.Glabri (J.E. Lange) D.J. Schaf., representing a linage of coprinoid fungi with plicated pileus but lacking a veil and setae. Singer (1948) expanded the concept of this lineage and treated *Coprinusauricomus* (now referred as *Parasolaauricoma* (Pat.) Redhead, Vilgalys & Hopple), which possesses setae on its pileus, as a distinct section, *Auricomi*. Subsequently, the taxonomic placement of the aforementioned sections underwent changes through the genus *Pseudocoprinus* Kühner and *Coprinus* Per. (Kühner and Romagnesi 1978; Orton and Watling 1979; Singer 1986; Uljé and Bas 1988; Uljé and Bender 1997), until Redhead et al. (2001), based on the phylogenetic findings of Hopple and Vilgalys (1994, 1999), established the genus *Parasola*. This establishment led to the widespread acceptance of its taxonomic status within the academic community, and it has been consistently recognized and used ever since (Schafer 2010; Hussain et al. 2016; Hussain et al. 2017; Szarkándi et al. 2017; Hussain et al. 2018; Ganga and Manimohan 2019; Zhu et al. 2022; Khan et al. 2023; Pošta et al. 2023). Furthermore, Redhead et al. reclassified sect.Glabri as sect.Parasola Redhead, Vilgalys & Hoppledue to their parasol-like appearance. Up to this point, the infrageneric classification of the genus *Parasola* comprises two sections, namely sect. *Parasola* and sect. *Auricomi*, with the distinction between the two lying in presence or absence of sclerocystidia.

Based on phylogenetic studies, Larsson and Örstadius (2008) discovered *Psathyrellaconopilea* belongs to the genus *Parasola*. Due to the presence of yellow-brown setae on its pileus, they classified this species under sect.Auricomi. However, since its basidiomata resemble those of genus *Psathyrella* and its pileus lacks striations, this finding challenged the traditional concept of *Parasola*. A series of studies have revealed that the inclusion of *Parasolaconopilea* (Fr.) Örstadius & E. Larss. and its closely related species results in sect.Auricomi being paraphyletic (Hussain et al. 2016; Hussain et al. 2017; Szarkándi et al. 2017; Hussain et al. 2018; Ganga and Manimohan 2019). Wächter and Melzer (2020) proposed a monophyletic taxonomic system for this genus, dividing the genus into sect. ConopileaeD. Wächt. & A. Melzer andsect.Parasola. According to their classification, the main difference of these two sections is that sect. Conopileaelacks radially sulcate pileus, whereassect.Parasola possesses this feature. As a result, the presence or absence of setae was no longer used as a key distinguishing feature. However, the discovery of *Parasolapsathyrelloides* K.G.G. Ganga & Manim., which exhibits a finely sulcate-plicated pileus yet is phylogenetically classified under sect.Conopileae, rendered this criterion ineffective for accurately differentiating between these two sections. Consequently, it remains worthwhile to investigate which morphological traits align with the monophyletic grouping of *Parasola*.

Nagy et al. (2009) revealed that species with psathyrelloid basidioma represents an early diverging taxon within *Parasola*. Subsequently, the evolution of parasoloid species—characterized by mature basidiomata resembling parasols—was accompanied by the emergence of several distinctive features, including a plicate pileus, pleurocystidia, brachybasidia, and partial deliquescence of the lamellae up maturation. Additionally, their basidiospores tend to transition from ellipsoid to polyangonal or heart-shaped, with the germ pore shifting from a central to an eccentric position. Malysheva (2019) reclassified *Galeropsisaporos* into *Parasola* as *Parasolaaporos* (Courtec.) E.F. Malysheva based on molecular phylogenetic analyses, although the taxonomic position of this secotioid species within this genus remains unclear. This species represents a third morphological form, alongside parasoloid and psathyrelloid species, within the genus. Given the discoveries of new species in recent years, prudent modifications to the proposed evolutionary framework may be necessary.

To date, eight species of *Parasola* have been reported from China: *Parasolaauricoma*, *Parasolaconopilea*, *Parasolalilatincta* (Bender & Uljé) Redhead, Vilgalys & Hopple, *Parasolalactea* [once were treated as *Parasolaleiocephala*], *Parasolaplicatilis*, *Parasolalilatinctoides* P. Voto (once were treated as *Parasolaschroeteri* (P. Karst.) Redhead, Vilgalys & Hopple), *Parasolasetulosa* (Berk. & Broome) Redhead, Vilgalys & Hopple and *Parasolamisera* (P. Karst.) Redhead, Vilgalys & Hopple. Except for *Parasolamisera*, which is distributed in tropical regions, the majority of these species are found in temperate zones (Tai 1979; Bi et al. 1997; Zhou and Wen 2007; Chi and Bau 2013; Li et al. 2015; Huang 2019; Zhu et al. 2022). However, given that most early records of this genus lack molecular evidence to verify their authenticity and that their macroscopic morphology exhibits a high degree of similarity, further research on this genus is highly necessary.

The objectives of this paper are threefold: (1) to comprehensively assess the diversity of *Parasola* species in China through extensive sampling, morphological observation, phylogenetic analyses, geographic distribution, and ecological characteristics; (2) to examine the morphological basis for the classification of monophyletic sections in the genus *Parasola*; (3) to investigate the anatomical variations underlying macro-morphological changes of *Parasola* species.

## ﻿Materials and methods

### ﻿Sampling and morphological characterization

Samples were collected in June-October, 2017–2022, in Jilin Province, Anhui Province, Hubei Province, Jiangsu Province, Zhejiang Province, Shanghai City, Inner Mongolia Autonomous Region, Xinjiang Uygur Autonomous Region and Guizhou Province, China. Specimens were photographed, tagged and recorded for ecological information before collecting in the field. All descriptions of color were based on Methuen Handbook of Colour (Kornerup and Wanscher 1978). Specimens were put in silica gel for at least 12 h for desiccating and kept in zip-lock bags. Voucher specimens are deposited in Herbarium of Mycology of Jilin Agricultural University (HMJAU), if not otherwise indicated.

The detailed features of specimens were observed under stereoscope (stemi 2000C, Zeiss Co., Ltd., Germany), including a number of large and small lamellae and the base of the stipe. The specimens were sectioned by hand and studied under light microscope (BX53, Olympus Co., Ltd., Japan). For anatomical studies slides were prepared in water, 5% aqueous KOH, 5% aqueous lactic acid, and Melzer’s reagent, respectively, added 1% Cango Red if necessary. Microscopic features including size, shape and color of basidiospores, basidia, pseudoparaphyses, cheilocystidia, pleurocystidia, pileipellis, stipitipellis, gill trama, subpileipellis and stipe trama with at least 40 structures were measured and the presence of clamp connection was observed in each sample. Free mature basidiospores collected from the surface of pileus or stipes were chosen for observation in front view and/or side view through 1000× magnification and measurement by software EP viewer (Olympus Co., Ltd., Japan) with precision to 0.01 μm; the diameter of germ pore was also measured, and the hilum was excluded. Statistical results of measured value presented in (a) b–c (d) form, b–c represented 90% confidence interval; (a) and (d) represented minimum and maximum value, respectively. Basidiospore sizes were presented as follows: length range × breadth range × width range for most species of *Parasola* with relatively flatten basidiospores. Q values were calculated as: Q = length divided by width; when breadth range was measured, Q_1_ = length divided by breadth range and Q_2_ = length divided by width (Uljé and Bas 1988; Nagy et al. 2009; Szarkándi et al. 2017); the shape terms corresponding to Q value were described according to Bas (1969). Other structures were measured and described through 400× magnification: in the case of basidia, the length of sterigma was excluded from the length of basidia; the measured values of cystidia included amorphous incrustation on surface; the value of the widest point was chosen as the width length of cystidia and basidia.

### ﻿DNA extraction, PCR amplification and DNA sequencing

Protocols for genomic DNA extraction, PCR amplification and sequencing followed those of Bau and Yan (2021) and Mou and Bau (2021). Three regions (ITS, LSU and tef1-α) were amplified for this study, which using ITS1F/ITS4 (White et al. 1990), LR0R/LR7 (Hopple and Vilgalys 1999), and 983F/1567R/2218R (Rehner et al. 2001) as primers, respectively. The DNA sequencing was done by Sangon Biotech Co. Ltd. (Shanghai, China). All newly generated sequences were deposited in GenBank (Table 1).

**Table 1.** Fungal species and sequences used in phylogenetic analyses.

Original identification	Revised identification	Voucher/strain number	Location	GenBank accession number				Reference
				ITS	LSU	tef1-α	β-tublin	
*Coprinopsisatramentaria*		SZMC-NL-4245	Hungary	FN396123	FN396172	FN396225		Nagy et al. 2011
*Coprinopsispannucioides*		SZMC-NL-3528	Hungary	FN396143	FN396202	FN396238		Nagy et al. 2011
*Homophroncernuum*		LO134-98	Sweden	DQ389726	DQ389726	KJ732828		Larsson and Orstadius 2008
*Lacrymariaglareosa*		LAS06-019	Sweden	KC992954	KC992954	KJ732827		Örstadius et al. 2015
Parasolaaff.lilatincta		SZMC:NL:0086	Hungary	FM163204	FM160705			Nagy et al. 2009
Parasolaaff.lilatincta		SZMC:NL:0096	Hungary	FM163205	FM160704			Nagy et al. 2009
*Parasolaaporos*		CL-F09.005	France	MK397586	MK397606			Malysheva et al. 2019
*Parasolaaporos*		CL-F09.008	France	MK397587	MK397607			Malysheva et al. 2019
*Parasolaaporos*		RC-F92.191	France	MH196349	MH196357			Malysheva et al. 2019
***Parasolaauricoma***		**HMJAU46429**	**China**	**OP861708**	**OP874857**	**PQ332982**		**this study**
***Parasolaauricoma***		**HMJAU64096**	**China**	**OP861709**				**this study**
***Parasolaauricoma***		**HMJAU46322**	**China**			**PQ332979**		this study
*Parasolaauricoma*		HMJAU46332	China	OL355051				Zhu et al. 2022
*Parasolaauricoma*		SZMC-NL-0087	Hungary	JN943107	JQ045871	FM897236	FN396252	Nagy et al. 2009
***Parasolachowii***		**HMJAU60358 (type)**	**China**	**OP874810**	**OP874895**			**this study**
***Parasolachowii***		**HMJAU60374**	**China**	**OP874811**	**OP874896**			**this study**
***Parasolachowii***		**HMJAU64099**	**China**	**OP874812**	**OP874876**			**this study**
***Parasolachowii***		**HMJAU64100**	**China**	**OP874813**	**OP874878**	**PQ332997**		**this study**
*Parasolacinnamomescens* Z. Khan, M.A. Arshad, Niazi & Khalid		LAH37333 (holotype)	Pakistan	OQ779055	OQ779058			Khan et al. 2023
*Parasolacinnamomescens*		LAH37335	Pakistan	OQ779057	OQ779060			Khan et al. 2023
*Parasolaconopilea*		CZ429	China	FJ755216	FJ755216			Wächter and Melzer 2020
*Parasolaconopilea*		SZMC-NL-0285	Hungary	FM163224	FM160685		FN396247	Nagy et al. 2009
*Parasolaconopilea*		SZMC-NL-0286	Hungary	FM163225	FM160684	FM897237		Nagy et al. 2009
*Parasolaconopilea*		LO186-02	Sweden	DQ389725	DQ389725			Larsson and Orstadius 2008
*Parasolaconopilea*		ZRL20151990	China	LT716064	KY418880			Zhao et al. 2017
***Parasolaconopilea***		**HMJAU60342**	**China**	**OP861711**	**OP874849**			**this study**
***Parasolaconopilea***		**HMJAU60365**	**China**	**OP861712**				**this study**
***Parasolaconopilea***		**HMJAU60368**	**China**	**OP874843**				**this study**
***Parasolaconstrictospora***		**HMJAU60336 (type)**	**China**	**OP874809**	**OP874854**	**PQ332986**		**this study**
***Parasolaconstrictospora***		**HMJAU60337**	**China**	**OP874808**	**OP874853**	**PQ332987**		**this study**
*Parasolacrataegi* Schmidt-Stohn		L. Nagy NL-4175 (type)	Hungary	KY928603	KY928631			Szarkándi et al. 2017
*Parasolacrataegi*		SSt98-239	Germany	KY928604				Szarkándi et al. 2017
*Parasolacrataegi*		SSt09-105	Germany	KY928606				Szarkándi et al. 2017
***Parasolacrispa***		**HMAJU60339 (type)**	**China**	**OP874824**	**OP874850**	**PQ332988**		**this study**
***Parasolacrispa***		**HMAJU60340**	**China**	**OP874825**	**OP874851**	**PQ332989**		**this study**
***Parasolacrispa***		**HMAJU60341**	**China**	**OP874826**	**OP874852**			**this study**
***Parasolacrispa***		**HMJAU64093**	**China**	**OP874827**	**OP874858**			**this study**
***Parasolacrispa***		**HMJAU64096**	**China**	**OP874828**	**OP874879**			**this study**
*Parasolacuniculorum*		K(M)191984	Britain	OL630105				Schafer et al. 2022
*Parasolacuniculorum*		DJS20120211001 (type)	Cyprus	OL630103			OQ850172	Schafer et al. 2022
***Parasolaeburnea***		**HMJAU60080**	**China**	**OP874805**	**OP874868**			**this study**
***Parasolaeburnea***		**HMJAU60081**	**China**	**OP874806**	**OP874873**			**this study**
***Parasolaeburnea***		**HMJAU60082**	**China**	**OP874807**	**OP874874**			**this study**
***Parasolaeburnea***		**HMJAU60346**	**China**	**OP874804**	**OP874891**	**PQ332977**		**this study**
***Parasolaeburnea***		**HMJAU60347**	**China**	**OP874803**	**OP874892**	**PQ332978**		**this study**
*Parasolagalericuliformis* (Losa ex Watling) Redhead, Vilgalys & Hopple	*Parasolalactea* (A.H. Sm.) Redhead, Vilgalys & Hopple	SZMC-NL-0095	Sweden	FM163188	FM160721			Nagy et al. 2009
*Parasolagalericuliformis*	*Parasolalactea*	SZMC-NL-6601	Hungary	FM163187	FM160722			Nagy et al. 2009
*Parasolaglabra* S. Hussain, Afshan, H. Ahmad & Khalid		LAH-SHP-23	Pakistan	KY461718	KY621805			Hussain et al. 2018
*Parasolaglabra*		LAH-SHP-5 (Holotype)	Pakistan	KY461717	KY621806	KY461735		Hussain et al. 2018
***Parasolagrisella***		**HMJAU60338 (type)**	**China**	**OP874829**	**OP874855**			**this study**
***Parasolagrisella***		**HMJAU64097**	**China**		**OP874862**			**this study**
***Parasolagrisella***		**HMJAU64098**	**China**	**OP874831**				**this study**
*Parasolahercules* (Uljé & Bas) Redhead, Vilgalys & Hopple		L:Ulje:1269	The Netherlands	FM163190	FM160719			Nagy et al. 2009
*Parasolahercules*		L146 (Holotype)	The Netherlands	HQ847027	HQ847112			Nagy et al. 2013
Parasolaaff.hercules	*Parasolamegasperma* (P.D. Orton) Redhead, Vilgalys & Hopple	L:Volders L78	The Netherlands	KY928630	KY928646			Szarkándi et al. 2017
*Parasolakuehneri* (Uljé & Bas) Redhead, Vilgalys & Hopple		L.C.B. Ulje 31-V-1987/type	The Netherlands	KY928608	KT928633		OQ850173	Szarkándi et al. 2017
***Parasolakuehneri***		**HMJAU60343**	**China**	**OP861494**	**OP874881**	**PQ332990**		**this study**
***Parasolakuehneri***		**HMJAU60344**	**China**	**OP861495**	**OP874880**	**PQ332991**		**this study**
***Parasolakuehneri***		**HMJAU60345**	**China**	**OP861496**	**OP874882**			**this study**
***Parasolakuehneri***		**HMJAU60077**	**China**	**OP861492**				**this study**
***Parasolakuehneri***		**HMJAU60087**	**China**	**OP861493**				**this study**
*Parasolakuehneri*		Ulje 1241	The Netherlands	HQ847026	HQ847111			Nagy et al. 2013
*Parasolalactea*		L. Nagy NL-0660b	Hungary	KY928611				Szarkándi et al. 2017
*Parasolalactea*		L. Nagy NL-2494	Hungary	KY928613				Szarkándi et al. 2017
*Parasolalactea*		L. Nagy NL-0678	Hungary	KY928612				Szarkándi et al. 2017
*Parasolaleiocephala* (P.D. Orton) Redhead, Vilgalys & Hopple	*Parasolalactea*	SZMC-NL-0283	Germany	JN943113	JQ045887	FM897239		Szarkándi et al. 2017
*Parasolaleiocephala*	*Parasolalactea*	SZMC-NL-0288	Sweden	JN943106	JQ045872	FM897233	FN396250	Szarkándi et al. 2017
*Parasolaleiocephala*	*Parasolalactea*	SZMC-NL-0466	Hungary	FM163192	FM160717		FN396254	Nagy et al. 2009
*Parasolalilatincta* (Bender & Uljé) Redhead, Vilgalys & Hopple		HUP-SH-P2	Pakistan	KP886463				Hussain et al. 2016
*Parasolalilatincta*	*Parasolalilatinctoides*	SZMC:NL:0468a	Hungary	FM163200	FM160709			Nagy et al. 2009
*Parasolalilatincta*		SZMC-NL-0472	Hungary	FM163199	FM160705			Nagy et al. 2009
*Parasolalilatincta*		SZMC-NL-0667	Hungary	FM163198.1	FM160711.1			Nagy et al. 2009
***Parasolalilatincta***		**HMJAU60356**	**China**	**OP861516**	**OP874886**			**this study**
***Parasolalilatincta***		**HMJAU60357**	**China**	**OP861517**	**OP874887**			**this study**
***Parasolalilatincta***		**HMJAU60375**	**China**	**OP861514**	**OP874869**	**PQ332994**		**this study**
***Parasolalilatincta***		**HMJAU60376**	**China**	**OP861513**	**OP874871**			**this study**
***Parasolalilatincta***		**HMJAU60377**	**China**	**OP861515**	**OP874872**			**this study**
***Parasolalilatincta***		**HMJAU60378**	**China**	**OP861512**	**OP874870**			**this study**
***Parasolalilatinctoides***		**HMJAU60355**	**China**	**OP874818**	**OP874883**	**PQ332993**		**this study**
***Parasolalilatinctoides***		**HMJAU60353**	**China**	**OP874819**	**OP874885**			**this study**
***Parasolalilatinctoides***		**HMJAU60354**	**China**	**OP874817**	**OP874884**			**this study**
***Parasolalilatinctoides***		**HMJAU60379**	**China**	**OP874820**	**OP874859**			**this study**
***Parasolalilatinctoides***		**HMJAU60384**	**China**	**OP874816**	**OP874861**			**this study**
***Parasolalilatinctoides***		**HMJAU60385**	**China**	**OP874815**				**this study**
***Parasolalilatinctoides***		**HMJAU64102**	**China**	**OP874814**				**this study**
*Parasolalitoralis* Loizides, D.J. Schaf. & P. Alvarado		DJS20120213004	Cyprus	OL630106				Schafer et al. 2022
*Parasolalitoralis*		DJS20130125001	Cyprus	OL630107				Schafer et al. 2022
*Parasolamalakandensis*		LAH-SHP-13	Pakistan	KU599828	KU599829	KU599831		Hussain et al. 2016
*Parasolamalakandensis*		LAH-SHP-17	Pakistan	KU599827	KU599830	KU599832		Hussain et al. 2016
***Parasolamalakandensis***		**HMJAU60360**	**China**	**OP861552**				**this study**
***Parasolamalakandensis***		**HMJAU64090**	**China**	**OP861549**	**OP874860**			**this study**
***Parasolamalakandensis***		**HMJAU64091**	**China**	**OP861550**				**this study**
***Parasolamalakandensis***		**HMJAU64092**	**China**	**OP861551**				**this study**
*Parasolamegasperma*		SZMC-NL-1924	Sweden	FM163208	FM160701	FM897232		Nagy et al. 2010b
*Parasolamegasperma*		L.C.B. Ulje 1275	Netherlands	KY928618	KY928637		OQ850175	Szarkándi et al. 2017
*Parasolamegasperma*		C:19683	Danmark	FM163206	FM163703			Nagy et al. 2009
***Parasolamegasperma***		**HMJAU64101**	**China**	**OP861519**	**OP874864**			**this study**
*Parasolamisera* (P. Karst.) Redhead, Vilgalys & Hopple		L. Nagy NL-0462	Hungary	KY928619	KY928638			Szarkándi et al. 2017
*Parasolamisera*		SZMC-NL-0677	Hungary	FM163211	FM160698	FM897240		Nagy et al. 2009
*Parasolamisera*		SZMC-NL-0490	Hungary	FM163209	FM160700			Nagy et al. 2009
*Parasolamisera*		SZMC-NL-0280 (neotype)	Hungary	FM163210	FM160699			Nagy et al. 2009
***Parasolaneoplicatilis***		**HMJAU64087 (holotype)**	**China**	**OP861544**	**OP874865**	**PQ332996**		**this study**
***Parasolaneoplicatilis***		**HMJAU64088**	**China**	**OP861545**	**OP874866**			**this study**
***Parasolaneoplicatilis***		**HMJAU64089**	**China**	**OP861546**				**this study**
*Parasolaneoplicatilis*		CBM-FB-40433	Vietnam	LC097063				Wächter and Melzer 2020
*Parasolaneoplicatilis*		CBM-FB-40243	Vietnam	LC097065				
*Parasolanudiceps* (P.D. Orton) Redhead, Vilgalys & Hopple	*Parasolaschroeteri* (P. Karst.) Redhead, Vilgalys & Hopple	HB19870911A	Germany	MK063783			OQ850176	Wächter and Melzer 2020
*Parasolaochracea*	*Parasolaschroeteri*	SZMC-NL-3167	Sweden	JN943136	JQ045865			Szarkándi et al. 2017
*Parasolaochracea*		SZMC-NL-3621 (Holotype)	Norway	JN943134	JQ045875			Szarkándi et al. 2017
***Parasolaorientolactea***		**HMJAU60078**	**China**	**OP874845**		**PQ332984**		**this study**
***Parasolaorientolactea***		**HMJAU60079**	**China**	**OP874844**	**OP874877**	**PQ332985**		**this study**
***Parasolaorientolactea***		**HMJAU60348**	**China**	**OP874846**				**this study**
***Parasolaorientolactea***		**HMJAU60350**	**China**	**OP874847**	**OP874888**			**this study**
***Parasolaorientolactea***		**HMJAU60352**	**China**	**OP874848**	**OP874890**			**this study**
*Parasolapapillatospora*		CNF 1/7858	Croatia	OQ862770	OQ862755	OQ850164	OQ850179	Pošta et al. 2023
*Parasolapapillatospora*		CNF 1/7600	Croatia	OQ862790	OQ862578	OQ850163		Pošta et al. 2023
*Parasolaplicatilis*		HMJAU46402	China	OL355159	OL376340			Zhu et al. 2022
*Parasolaplicatilis*		L. Nagy NL-2949	Hungary	KY928624	KY928642		FN396251	Szarkándi et al. 2017
*Parasolaplicatilis*		L. Nagy NL-3525	Hungary	KY928625	KY928643		FN396253	
*Parasolaplicatilis*		SZMC:NL:0477	Hungary	FM163212	FM160697		FN396245	Nagy et al. 2011
***Parasolaplicatilis***		**HMJAU60359**	**China**	**OP861532**	**OP874894**			**this study**
*Parasolaplicatilis*		HMJAU46405	China	OL355167	OL376339	**PQ332981**		Zhu et al. 2022
***Parasolaplicatilis***		**HMJAU60366**	**China**	**OP861533**				**this study**
***Parasolaplicatilis***		**HMJAU46460**	**China**	**OP861531**	**OP874891**	**PQ332983**		**this study**
*Parasolaplicatilis*		SZMC-NL-0075a (epitype)	Hungary	FM163213	FM160696			Nagy et al. 2009
*Parasolaplicatilis*		SZMC-NL-0295	Hungary	FM163216	FM160693	FM897242		Nagy et al. 2009
*Parasolaplicatilis-similis* L. Nagy, Szarkándi & Dima		SZMC-NL-2125 (Holotype)	Sweden	KY928620				Szarkándi et al. 2017
*Parasolaplicatilis-similis*		L. Nagy NL-3980	Slovakia	KY928621	KY928639		OQ850180	Szarkándi et al. 2017
*Parasolapsathyrelloides* K.G.G. Ganga & Manim.		CAL 1753 (type)	India	MK682756	MK682754			Ganga and Manimohan 2019
*Parasolapsathyrelloides*		AMH10119	India	MK682752				Ganga and Manimohan 2019
*Parasolapseudolactea* Sadiqullah, S. Hussain & Khalid		HUP-SU-412 (Holotype)	Pakistan	KY461719	KY621799	KY461733		Hussain et al. 2018
*Parasolapseudolactea*		HUP-SU-413	Pakistan	KY461720	KY621800	KY461734		Hussain et al. 2018
*Parasolaschroeteri*	*Parasolalilatinctoides*	HMJAU46363	China	MW822899	OL376321			Zhu et al. 2022
*Parasolaschroeteri*	*Parasolalilatinctoides*	HMJAU46370	China	MW822898	OL376318			Zhu et al. 2022
*Parasolaschroeteri*/*lilatincta*	*Parasolalilatinctoides*	LAH-SHP-8 (type)	Pakistan	KY461722	KY461725	KY461731		Hussain et al. 2018
*Parasolaschroeteri*/*lilatincta*	*Parasolalilatinctoides*	LAH-SHP-31	Pakistan	KY461723	KY461726	KY461732		Hussain et al. 2018
*Parasolaschroeteri*	*Parasolamegasperma*	L.C.B. Ulje 1140	The Netherlands	KY928628				Szarkándi et al. 2017
*Parasolaschroeteri*	*Parasolamegasperma*	Dahncke 1502	Germany	KY928616	KY928635			Szarkándi et al. 2017
*Parasolaschroeteri*	*Parasolaplicatilis-similis*	SZMC-NL-0287	Sweden	FM163218	FM160691			Szarkándi et al. 2017
*Parasolasetulosa*		HMJAU46367	China	MW822929	OL376319	**PQ332980**		Zhu et al. 2022
*Parasolasetulosa*		L32	Hungary	HQ847030	HQ847115			Nagy et al. 2013
*Parasolasetulosa*		SFC20150812-15	South Korea	MF445222				Cho et al. 2018
***Parasolasetulosa***		**HMJAU64099**	**China**	**OP861677**				**this study**
***Parasolatenuissima***		**HMJAU64084 (type)**	**China**	**OP874821**	**OP874867**	**PQ332995**		**this study**
***Parasolatenuissima***		**HMJAU64085**	**China**	**OP874822**	**OP874863**			**this study**
***Parasolatenuissima***		**HMJAU64086**	**China**	**OP874823**	**OP874875**			**this study**

### ﻿Alignment and phylogenetic analyses

Newly generated sequences were edited using Sequencher 4.1.4 (Gene Codes, Ann Arbor, MI, United States) and haplotypes of heterozygotes were resolved according to Hughes et al. (2013). The missing or ambiguous loci were coded as “N”. Other sequences for phylogenetic analyses were downloaded from GenBank following Nagy et al. (2009), Nagy et al. (2011), Örstadius et al. (2015), Szarkándi et al. (2017), Hussain et al. (2017), Zhao et al. (2017), Cho et al. (2018), Hussain et al. (2018), Ganga et al. (2019), Malysheva (2019), Vizzini et al. (2019), Wächter and Melzer (2020), Schafer et al. (2022) and Zhu et al. (2022) (Table 1). Nucleotide sequences from each gene were aligned using MAFFT v.7.245 (Katoh and Standley 2013) and the ambiguous alignment were adjustment manually with MEGA7 (Kumar et al. 2016). Ambiguously aligned regions were removed from the datasets with Gblocks in Phylosuite undergoing the parameters: minimum number of sequences for a conserved position: 146; minimum number of sequences for a flank position: 146; maximum number of contiguous non-conserved positions: 8; minimum length of a block: 2; allowed gap positions: with half (Talavera and Castresana 2007; Wu et al. 2014; Zhang et al. 2020). Sequences of *Coprinopsisatramentaria* (Bull.) Redhead, Vilgalys & Moncalvo, *C.pannucioides* (J.E. Lange) Örstadius & E. Larss., *Homophroncernuum* (Vahl) Örstadius & E. Larss. and *Lacrymariaglareosa* (J. Favre) Watling were chosen as outgroups for analysis.

The maximum likelihood (ML) and Bayesian inference (BI) methods were used to analyze the combined and single-gene datasets of ITS, nrLSU, tef1-αand β-tublin. Multimarkers were concatenated as a combined file with SequenceMatrix (Vaidya et al. 2011). The congruences of combined datasets of ITS-LSU, and ITS-LSU-tef1-α and ITS-LSU-tef1-α-β-tublin evaluated with the incongruence length difference (ILD) test performed by PAUP* version 4.0b10, undergoing heuristic search and 1000 homogeneity replicates (Farries et al. 1994; Swofford 2002). For maximum likelihood (ML) analyses, GTRGAMMAI model and 1000 bootstrap resamples in separate partitions were conducted by RAxML-HPC BlackBox on Cipres or raxmlGUI2.0 (Miller et al. 2010; Stamatakis et al. 2014). For Bayesian Inference (BI) analysis, the best-fit models of nucleotide evolution for each dataset were operated in MrModeltest v.2.3 (Nylander et al. 2008) and gaps were treated as missing data; consequent phylogenetic analysis was conducted in MrBayes v.3.2.6 (Ronquist et al. 2012). Four Markov chains were run for 3,000,000 generations with sampling every 100 generations until the split deviation frequency value was less than 0.01 (Ronquist and Huelsenbeck 2003). Bootstrap support (BS) values greater than 70% (RAxML analyses) and Bayesian posterior probabilities (BPP) higher than 0.95 (Mrbayes analyses) were regarded as significantly supported, BS between 50% and 70% and BPP between 0.90 and 0.95 were considered as weakly supported, otherwise it is thought to be unresolved (Huelsenbeck et al. 1993; Leaché et al. 2002).

### ﻿Phylogenetic species delimitation and polyphasic taxonomic information matching

We used the Genealogical Concordance Phylogenetic Species Recognition (GCPSR) method to identify the phylogenetic species which should be at least supported with high support value (BI, ML) in a single-loci phylogenetic dataset (Taylor et al. 2000; Dettman et al. 2003; Han et al. 2020). We chose one representative collection for each phylogenetic species and used the three-loci (ITS-LSU-tef1-α) dataset to build a species tree that match the polyphasic classification data, including five macromorphological features, seven micromorphological features, an ecological characteristic and a geographical distribution information, viz. shape, color and size of pileus; presence of plication on pileus; connection type between stipe and lamellae; presence of secondary pileipellis; arrangement, shape, and size of caulocystidia at the top part of stipe; size, color, shape in face view and position of germ pore of basidiospores; and morphological type of basidia. Since the difference between cheilocystidia and pleurocystidia is relatively small and there is a lack of data on subpileipellis, subhymenium, lamellae trama, stipitipellis and stipe trama from more species, the aforementioned morphological characteristics were not used to match here.

According to the definition of Bas (1969), the basidiomata of this lineage are mainly tiny to small-sized (diameter of pileus < 30 mm), occasionally medium-sized. Therefore, we did not adopt the abovementioned general definition but redefined the relative size of the matured fruiting bodies of species in this genus based on actual observations, i.e., large (L: pileus diameter ≥ 10 mm) and small (S: pileus diameter < 10 mm). Shapes of pileus could be conical or flattened. In consideration of the color of pileus changes in growth, we compared the unfaded color (in young basidiomata or at the center of pileus when matured) and the colors could be summarized as: (1) lactous; (2) light purple; (3) light yellow brown; (4) yellow brown; (5) orange brown; or (6) red brown. The matured pileus is plicated or not: (1) yes (Y); or (2) not (N). The presence or absence of secondary pileipellis when mature has two types: (1) secondary pileipellis present (P); or (2) secondary pileipellis absent (A). The presence or absence of sclerocystida on pileus has two states: (1) sclerocystidia present (P); or (2) sclerocystidia absent (A). The connection between stipe and lamellae is divided into three types: (1) adnate (AD); (2) free without obvious pseudocollarium (F); or (3) free with obvious pseudocollarium (FA). The arrangement of caulocystidia at upper part of stipe could be: (1) evenly distributed; or (2) accumulated at terminal part. The basidiospores of this lineage is coded as: (1) small (S: < 10 μm in length); (2) medium-sized (M: 10–12 μm in length); (3) large (L: 12–15 μm in length); or (4) very large (XL: ≥ 15 μm in length). The shape in face view of basidiospores could be summarized as seven types: (1) (sub)globose (G); (2) ellipsoid to oblong (E); (3) ovoid (O); (4) rounded triangular (RT); (5) rhombic (R); (6) rounded pentagonal (RP); or (7) rounded hexagonal (RH); if the basidiospores are constricted or possess two germ pores, they are additionally labeled “-C” and “-D”, respectively. The germ pore on basidiospores has two positions: (1) central; or (2) eccentric. The morphological types of basidia have two states: (1) monomorphic (M: difference between the maximum and minimum length of basidia less than 10 μm); or (2) dimorphic (D: difference between the maximum and minimum length of greater than 10 μm). *Parasola* possesses three types of substrates: (1) soil of grassland or forest (in yellow); (2) woodchips or buried wood (in green); or (3) dung of herbivorous animals (in brown). The geographical distribution could be coded as: (1) wide-speared (W); (2) tropical (T); (3) north subtropical (NS); or (4) north temperate (NT).

## ﻿Results

### ﻿Phylogenetic analyses

**Figure 1.**
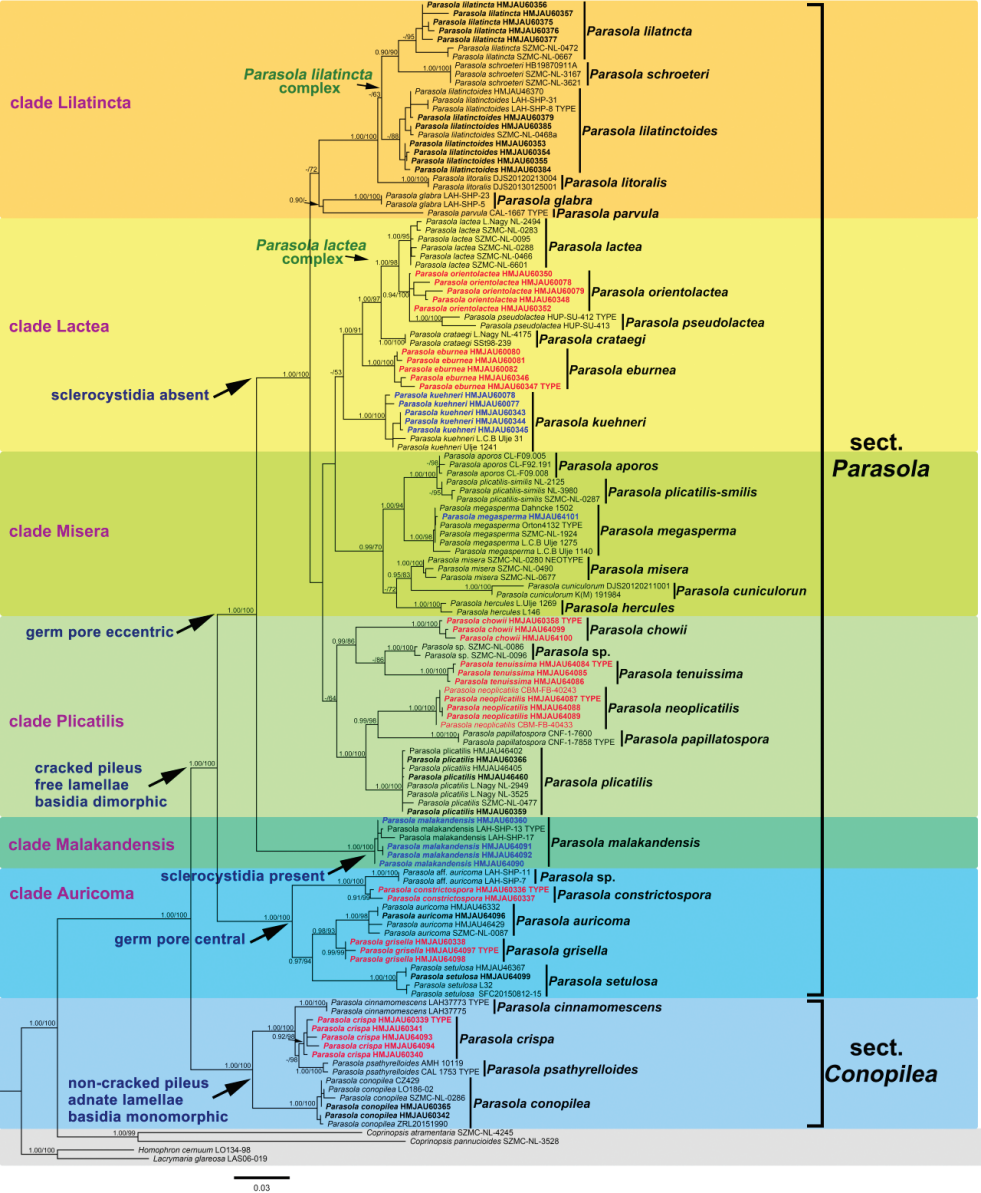
Phylogeny inferred from four-locus dataset (ITS+LSU+tef1-α+β-tublin), with branch lengths based on the Maximum Likelihood analysis. Only BS over 50% and BPP over 0.90 are given in the tree. Eight newly described species and three new records to China are indicated in red and blue bold letters respectively. The blue, green and yellow background indicates sect.Conopileae, transitional taxa and typical taxa of sect.Parasola respectively. The two sections are displayed on the right of the phylogram.

**Figure 2.**
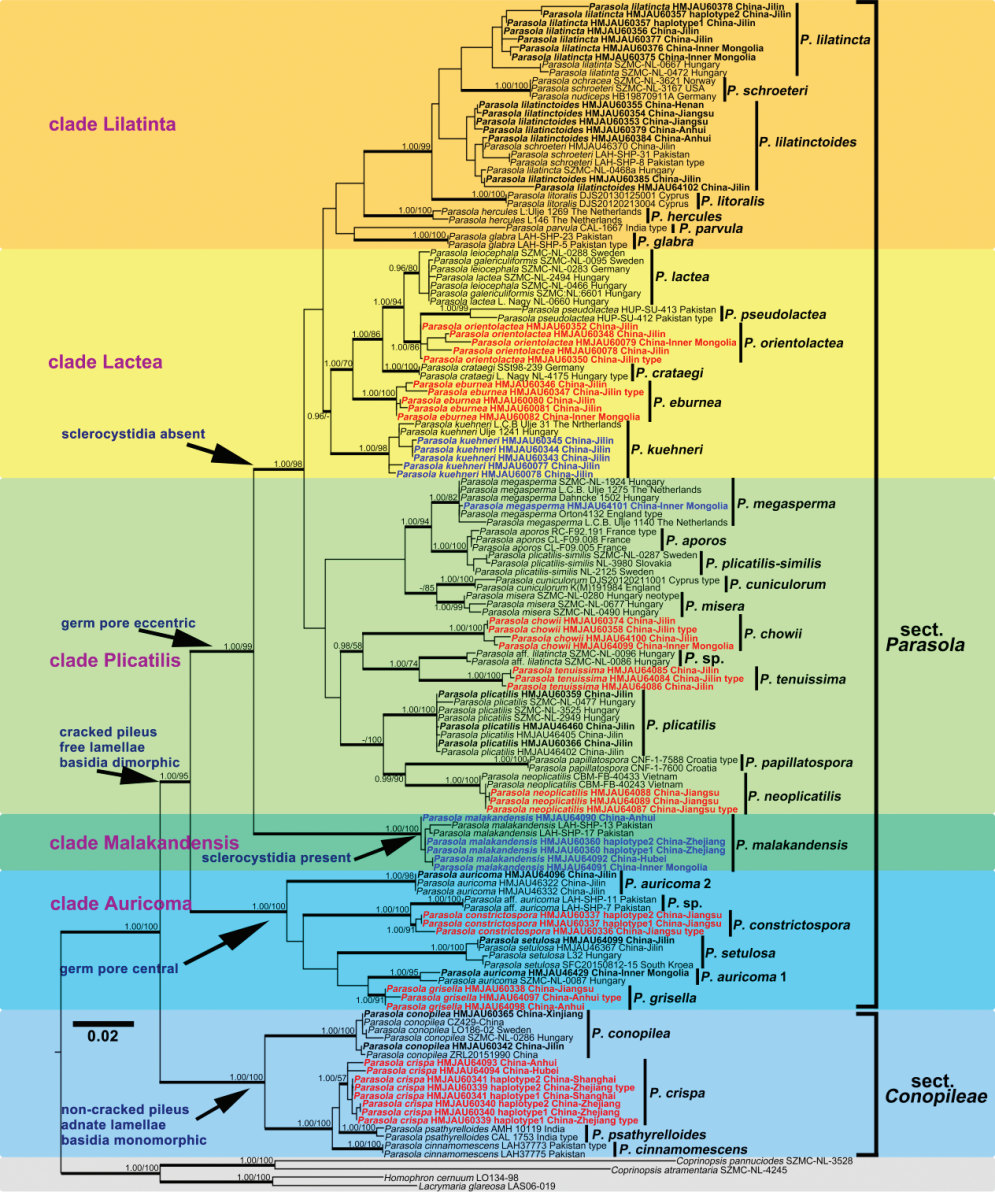
Phylogeny inferred from three-locus dataset (ITS+LSU+tef1-α), with branch lengths based on the Maximum Likelihood analysis. Only BS over 50% and BPP over 0.90 are given in the tree. Eight newly described species and three new records to China are indicated in red and blue bold letters respectively. The blue, green and yellow background indicates sect.Conopileae, transitional taxa and typical taxa of sect.Parasola respectively. The two sections are displayed on the right of the phylogram.

**Figure 3.**
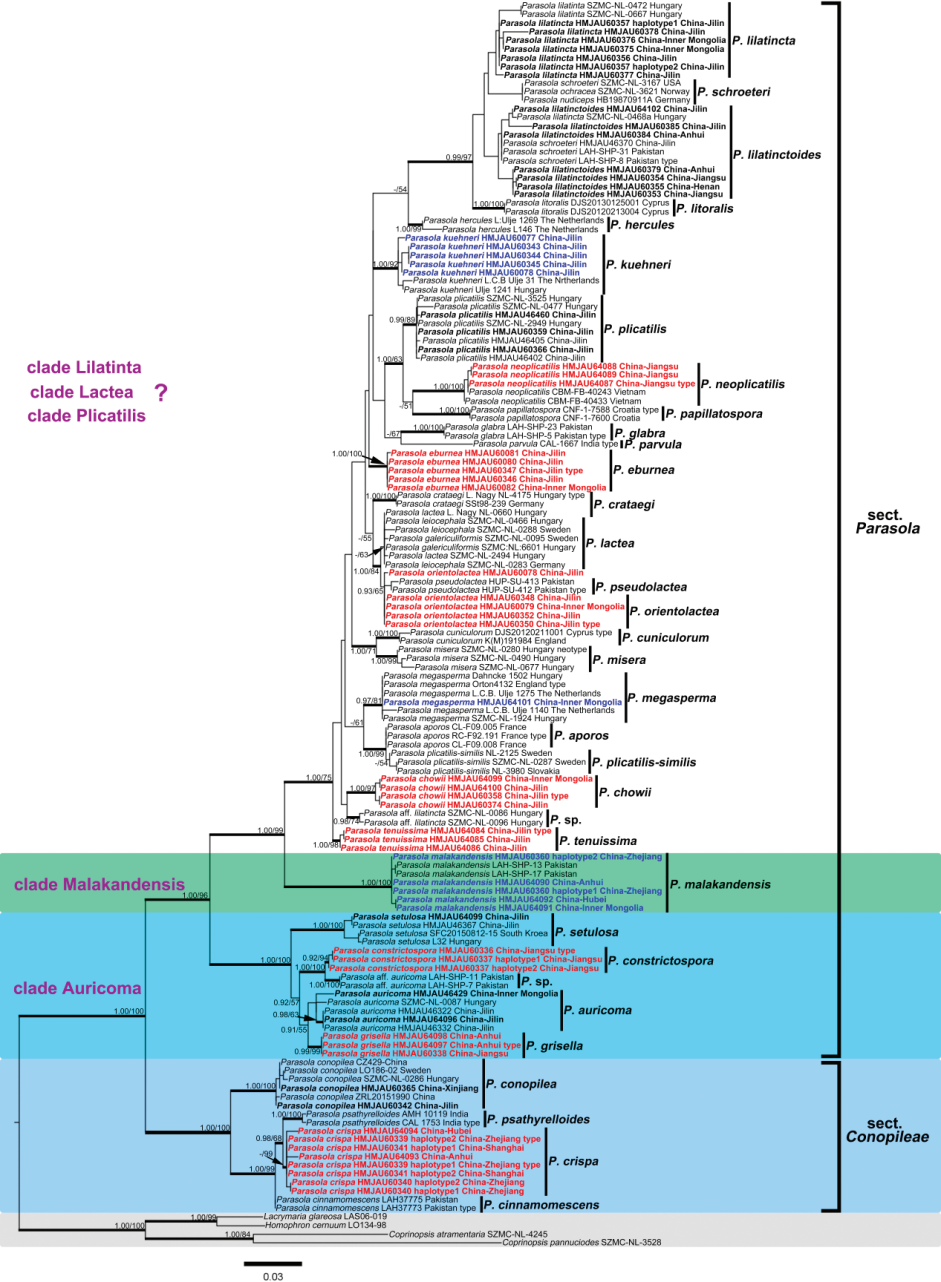
Phylogeny inferred from ITS dataset, with branch lengths based on the Maximum Likelihood analysis. Only BS over 50% and BPP over 0.90 are given in the tree. Eight newly described species and three new records to China are indicated in red and blue bold letters respectively. The blue, green and yellow background indicates sect.Conopileae, transitional taxa and typical taxa of sect.Parasola respectively. The two sections are displayed on the right of the phylogram.

**Figure 4.**
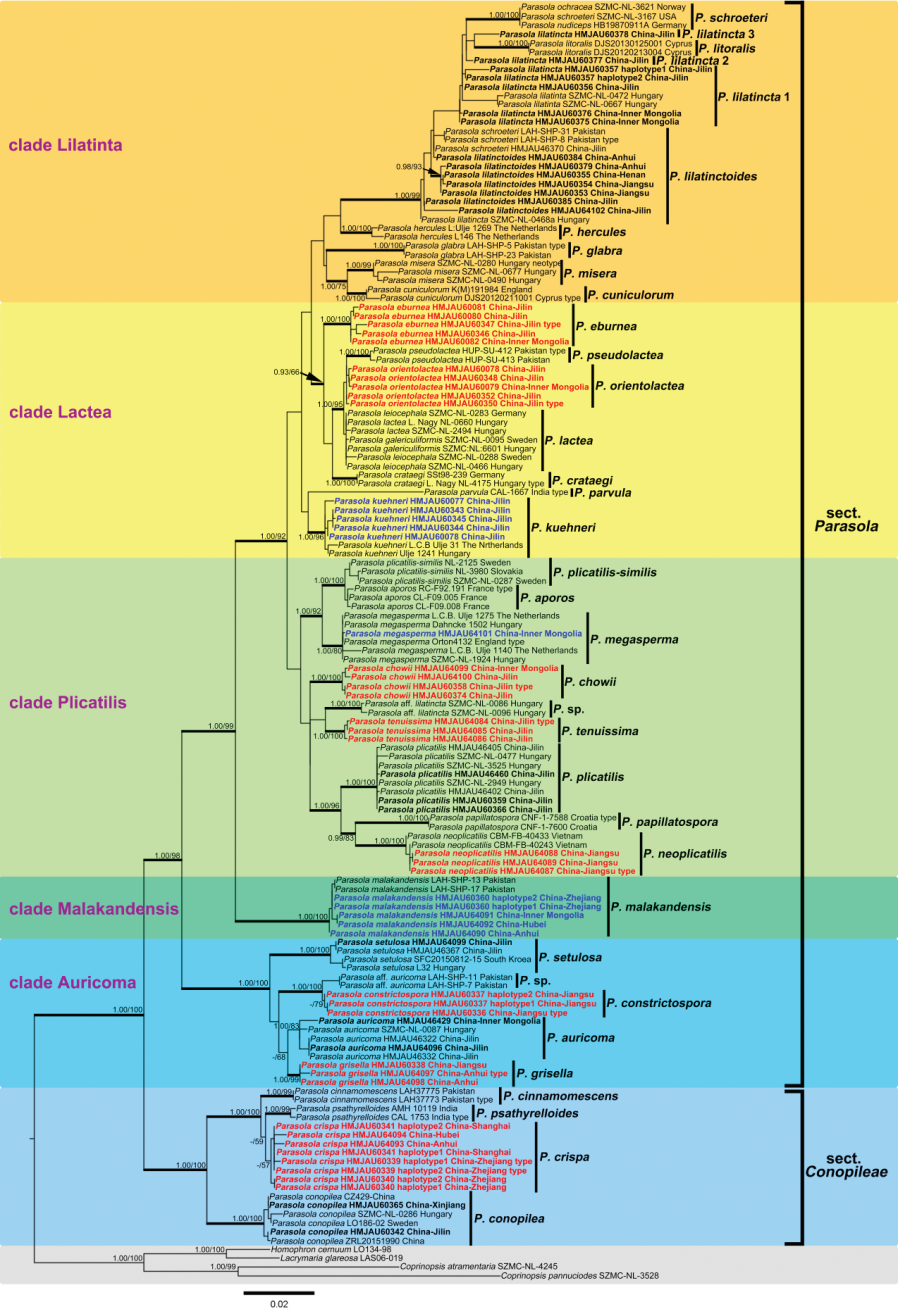
Phylogeny inferred from ITS+LSU dataset, with branch lengths based on the Maximum Likelihood analysis. Only BS over 50% and BPP over 0.90 are given in the tree. Eight newly described species and three new records to China are indicated in red and blue bold letters respectively.

In this study, 285 sequences derived from three gene locus (ITS, LSU and tef1-α) were used to reconstruct phylogenetic trees of *Parasola*, 144 of them were newly generated, including 67 sequences of ITS, 51 of LSU and 26 of tef1-α. Besides, 17 β-tublin sequences were also downloaded from GenBank. The phylogenetic tree resulting from ML and BI analyses for two combined datasets were largely similar to each other and reflected current views on the genus (Figs 1–4) though with few differences in support value. The partition homogeneity test indicated that four genes display a congruent phylogenetic signal (P value = 1.00). The best-fit evolutionary model selected by ModelGui for three regions were all invgamma. The combined dataset (ITS+LSU+tef1-α+β-tublin) had an aligned length of 2963 characters (701 from ITS, 1122 from LSU, 612 from tef-1α and 527 from β-tublin) and included sequences from 141 specimens representing 33 taxa of *Parasola*. The calculated values represented strong supported (BS ≥ 75, BPP ≥ 0.95) and weak supported (BS ≥ 50, BPP ≥ 0.90) were showed in Figs 1–4.

#### ﻿Parasolasect.Conopileae

Three known species *Parasolaconopilea*, *Parasolapsathyrelloides*, *Parasolacinnamomescens*, and one proposed new species, *Parasolacrispa*, were found in sect.Conopileae and as the basal groups of the genus *Parasola* with strong support.

#### ﻿Parasolasect.Parasola

Bayesian and ML analyses with the four-loci dataset revealed a great diversity in sect.Parasola with six clades, representing Clade Auricomi, Malakandensis, Plicatilis, Misera, Lilatinta and Lactea. Among them, three clades (Auricomi, Malakandensis, and Misera) were highly supported (BPP/ML = 1.00/100, 1.00/100, and 0.99/70, respectively), while the other three clades, namely Plicatilis, Lilatincta and Lactea, are with weak support (BPP/ML = -/64, -/53, and -/72, respectively) (Fig. 1). However, in phylogenetic frameworks conducted with ITS+LSU+ tef1-α, ITS+LSU, and ITS datasets, the aforementioned clades could only be partially resolved.

Seven newly found lineages were identified in this section with strongly supported as monophyletic by Bayesian and ML results (BPP ≥ 0.95, ML ≥ 75%) conducted by both combined and single gene datasets.

#### ﻿Clade Auricoma

Based on the four-loci dataset and the ITS (± LSU) dataset, the clade comprised five phylogenetic lineages. However, within the phylogenetic framework constructed using the combined ITS+LSU+tef-1α dataset, this branch encompassed phylogenetic species, resulting in the polyphyly of *Parasolaauricoma*.

Although *Parasolagrisella* is sister to *Parasolaauricoma*, the proposed new species forms a distinct, well-supported lineage (BPP/ML = 0.99/99). *Parasolaconstrictospora* occupies an independent branch within this clade, sister to undetermined species (Parasolaaff.auricoma) collected from Pakistan. In the four-loci phylogenetic framework, all species within the clade were strongly supported.

#### ﻿Clade Malakandensis

Currently, only one species, *Parasolamalakandensis*, is recognized within this clade. The four samples collected from China formed a highly supported cluster with the type specimens from Pakistan (BPP ≥ 0.95, ML ≥ 75%), showing no significant divergence, which strongly suggests that they belong to the same species.

#### ﻿Clade Plicatilis

Six well-supported species were found in this clade, including three new proposed species, namely *Parasolatenuissima*, *Parasolachowii* and *Parasolaneoplicatilis* respectively. Among them, *Parasolaneoplicatilis* is sister to *Parasolapapillatospora*, while *Parasolachowii* is phylogenetically close to *Parasolatenuissima*.

#### ﻿Clade Misera

Clade Misera contains six known species, namely *Parasolaaporos*, *Parasolaplicatilis-similis*, *Parasolamegasperma*, *Parasolamisera*, *Parasolacuniculorum*, and *Parasolahercules*. The specimen HMJAU64101 collected from China was proved to be *Parasolamegasperma* (PP = 1.00, BPP = 89%) and it is the first record of this species in Asia.

#### ﻿Clade Lilatincta

Six phylogenetic species were identified as members of this group. Among them, *Parasolalilatinctoides*, *Parasolaschroeteri* and *Parasolalilatincta* may constitute a species complex, because the independence and monophyly of three species was only proved in phylogenetic framework constructed using the ITS+LSU+tef1-α+β-tublin dataset. Specimens from China previously labeled as *Parasolalilatincta* or *Parasolaschroeteri* were attributed to *Parasolalilatincta* or *Parasolalilatinctoides*, confirmed by the four-loci phylogenetic study. Additionally, the distribution of *Parasolaschroeteri* in China remains unconfirmed.

#### ﻿Clade Lactea

This clade comprised six well-supported phylogenetic species: *Parasolacrataegi*, *Parasolapseudolactea*, *Parasolakuehneri*, *Parasolalactea*, and two newly discovered species, *Parasolaeburnea* and *Parasolaorientolactea*. Specimens of *Parasolakuehneri* collected from northeastern China clustered with those from Europe, confirming their conspecificity based both molecular and morphological evidence. *Parasolaeburnea* constitutes an independent branch distinct from known species, suggesting it represents a novel taxon. *Parasolalactea*, *Parasolapseudolactea* and *Parasolaorientolactea* are closely related, with *Parasolaorientolactea* occupying a transitional position between the other two species. The phylogenetic pattern aligns with morphological observation and geological distribution.

## ﻿Taxonomy

### ﻿ Parasola

Taxon classificationFungiAgaricalesPsathyrellaceae

Redhead, Vilgalys & Hopple

4BA75A4D-DEFC-5241-B28B-40B54D07A1C5

#### Description.

Basidiomata small to medium-sized, most terrestrial, sometimes fimicolous or lignicolous. Pileus radially sulcate or almost glabrous, veil absent, sometimes with brown hair, center with depressing disc or not. Lamellae adnate or free, withering or partial deliquescent at age. Stipes hollow, most glabrous. Basidiospores flattened or not, subglobose, ellipsoid, ovoid, rounded subtriangular, rhombic, subpentangular, hexagonal in front view, ellipsoid or lentiform in side view, germ pore central or eccentric. Basidia monomorphic or dimorphic, usually 4-spored, occasionally 2-spored. Lamellae trama regular, colorless or in brown hue. Lamellae margin infertile, cheilocystidia abundant, subglobose, utriform, ellipsoid, sublageniform or subcylindrical. Pleurocystidia are mainly present, subglobose, utriform, sublageniform or subcylindrical. Original pileipellis a hymeniform, secondary pileipellis is a subcutis when present. Caulocystidia is only present at the upper part of stipes. Clamps present and sometimes present pseudoclamps.

**Figure 5.**
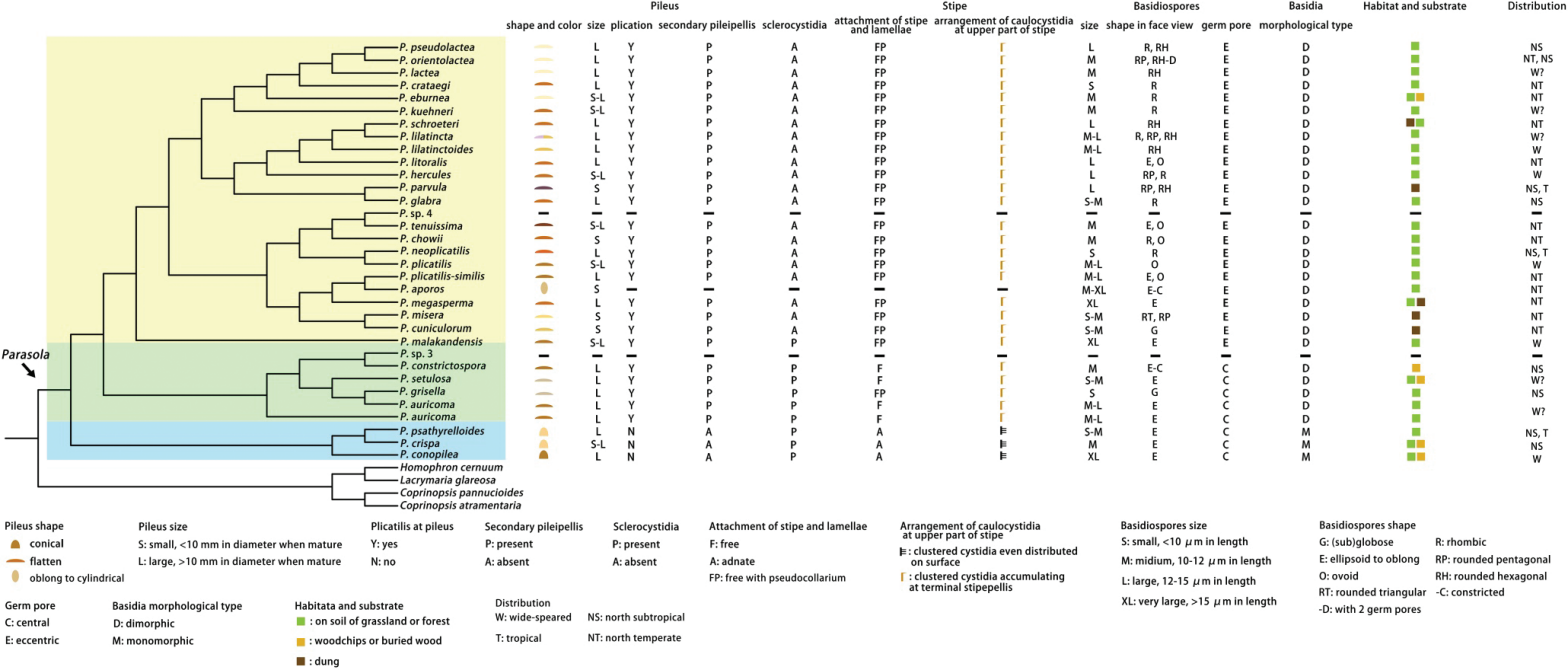
Selected morphological characteristics and ecological information of species in *Parasola* were mapped with the consensus tree of three-locus dataset (ITS+LSU+tef1-α) conducted by one representative collection for each species. Species in sect.Conopileae, transitional taxa and typical taxa of sect.Parasola were enclosed in blue, green and yellow frames respectively. Features of species were given under the consensus tree. Uncertain state for a taxon was given as “-”, other abbreviations were presented and explained

Two sections in *Parasola* are inferred based on molecular data, scoring of macro- and micro-morphological traits (Fig. 5). Macromorphologically, consistent characters supporting the two sections include the pileus shape and the lamellae-stipe attachment type, while micromorphologically diagnostic features encompass the presence or absence of secondary pileipillis, and the basidia morphological type.

### ﻿ Parasola sect. Conopileae

Taxon classificationFungiAgaricalesPsathyrellaceae

Wächter & A. Melzer

BDAF011A-1B48-57CD-9F90-46A2FCA8AF49

#### Description.

Basidiomata small to medium-sized, terrestrial or lignicolous. Pileus conical until mature, almost glabrous to finely sulcate but never cracking into plication, veil absent, brown hairs present. Lamellae adnate, withering at age. Stipes hollow, most glabrous. Basidiospores not flattened, ellipsoid to ovoid in front view and side view, germ pore central. Basidia monomorphic, regularly 4-spored. Lamellae trama regular, colorless or in brown hue. Lamellae margin infertile, cheilocystidia abundant, subglobose, utriform, ellipsoid, sublageniform. Pleurocystidia present or not, when present subglobose, utriform, sublageniform or subcylindrical. Original pileipellis a hymeniform, composed of sphaeropedunculate cells, secondary pileipellis absent. Sclerocystidia present. Caulocystidia present at upper part of stipe, uniformly or in small cluster distributed, subglobose, utriform, sublageniform or (sub)cylindrical. Clamps present and sometimes present pseudoclamps.

### ﻿ Parasola crispa

Taxon classificationFungiAgaricalesPsathyrellaceae

T. Bau, L.Y. Zhu & W.F. Lin sp. nov.

5ADBA7E1-487A-5F3D-BAF2-CC3F2A34F543

MB468520

Figs 6f–k , 7

#### Etymology.

The specific epithet refers to the obscure sulcate-striate pileus of this species.

#### Diagnosis.

Basidiocarps psathyrelloid; pileus conico-convex to paraboloid when mature, sometimes with obvious papilla, lacteous to pale brown-gray, somewhat yellow-brown at center, surface subglabrous to uneven, mainly with obscure sulcate-striate up to 1/3 to 1/2 part from margin to center; lamellae adnate; stipes glabrous with clavate base; basidiospores 10.3–10.7 × 7.3–7.6 × 6.4–7.0 μm, ellipsoid or ovoid in front view and ellipsoid to oblong in side view, germ pore central or slightly eccentric, 1.8–3.3 μm wide; basidia monomorphics, with relatively long sterigma which is up to 8 μm; sclerocystidia present; caulocystidia present, mostly sublageniform or subcylindrical.

#### Type.

**CHINA** • Zhejiang Province, Hangzhou City, Zijingang Campus of Zhejiang University, 30°29'73"N, 120°08'70"E, 51 m a.s.l., on soil mixed with rotten grass in grassland, July 5^th^ 2021, T. Bau, W. F. Lin and L. Y. Zhu, Z21070513 (holotype HMJAU60339).

#### Description.

Basidiocarps small, psathyrelloid, not collapsing. Pileus 6–7 × 6–8 mm at young stage, 10–18 mm when mature, at first conical, finally conico-convex to paraboloid and never flattened, sometimes with obvious papilla; dry; sordid yellow to light cinnamon when young, lacteous to pale brown-gray, somewhat yellow-brown at center when age; surface subglabrous to uneven, sometimes with obscure sulcate-striate up to 1/3 to 1/2 part from margin to center. Context thin, white to pale gray, odor and taste not distinctive. Lamellae crowded, adnate, 1–3 mm in wide, L = 32–39, I = 1 or 3, initially pale gray then become brown gray with somewhat olivaceous hue and finally purple gray or dark gray when mature, more pale at margin; not deliquescent. Stipe 31–74 × 1–2 mm, cylindrical, hollow, equal or attenuate towards the apex, white to sordid yellow, glabrous, clavate and without white hairs at base of stipes. Spore print not recording.

Basidiospores [49, 4, 3] (9.3–)10.3–10.7(–11.4) × (6.6–)7.3–7.6(–8.2) × (5.8–)6.4–7.0(–7.2) μm (10.5 × 7.4 × 6.7 μm in average), Q_1_ = 1.29–1.51, Q_2_ = 1.46–1.80, av. Q_1_ = 1.41, av. Q_2_ = 1.58; ellipsoid or ovoid in front view and ellipsoid to oblong in side view, with apical papilla, with conical base and truncate apex, smooth, dark yellow-brown to almost black; inamyloid; germ pore central or slightly eccentric, 1.8–3.3 μm wide. Basidia monomorphics, 20–37 × 8–13 μm, with relatively long sterigma which up to 8 μm, clavate, hyaline, 4- or 2-spored, surrounded with 4–6 pseudoparaphyses; subhymenium composed of subglobose, ellipsoid, oblong or cylindrical elements, 8–20 × 6–16 μm. Cheilocystidia abundant, 26–76 × 15–28 μm, subglobose, utriform, ellipsoid or sublageniform, smooth, colorless, thin-walled. Pleurocystidia relatively rare, when present, 41–79 × 11–32 μm, broad utriform, sublageniform or (sub)cylindrical. Lamella trama regular, 3–12 μm wide, hyaline, colorless, thin-walled. Pileipellis a hymeniderm mainly made up of sphaeropedunculate cells with inconspicuous short pedicels, 20–43 × 14–21 μm, hyaline, with brown hue at base in most cases, mixed with sclerocystidia; sclerocystidia 28–159 × 4–6 μm, yellow-brown, thick-walled; pileus trama hyphae densely interwoven, thin-walled, hyaline, dark yellow to yellow-brown, 4–6 μm wide. Caulopellis hyphae parallel, 4–9 μm wide, hyaline, thin-walled, slightly diverticulate; hyphae of stipe trama 9–21 μm wide, colorless, thin-walled; caulocystidia present at upper part of stipe, 30–53 × 11–16 μm, mostly in cylindrical or broad lageniform, 16–21 × 11–16 μm, sometimes subglobose to ellipsoid, hyaline, thin-walled. Clamp connection abundant, pseudoclamp also present.

#### Ecology.

Solitary, subfasciculate, or in small groups, grow in lawns and other grassy places, at base of dead trump of broadleaf trees or humus layer of bamboo forest. Fruiting during June to August. Currently only known from East and Central China.

#### Other specimens examined.

**CHINA** • Zhejiang Province, Hangzhou City, Zijingang Campus of Zhejiang University, 30°29'90"N, 120°08'61"E, 51 m a.s.l., July 5^th^ 2021, T. Bau, W. F. Lin and L. Y. Zhu, HMJAU60340 (Z21070507); **CHINA** • Shanghai Municipality, Songjiang District, Tianma Mountain, 31°07'48"N, 121°15'39"E, 82 m a.s.l., June 29^th^ 2021, J. M. Cai, HMJAU67660 (CJM62901); • August 28^th^ 2022, J. M. Cai, HMJAU60341 (SHPC); **CHINA** • Anhui Province, Chizhou City, Shitai County, bamboo forest opposite Shitai Environmental Protection Agency, 30°12'43"N, 117°29'29"E, 55 m a.s.l., June 16^th^ 2022, L. Y. Zhu, H. B. Song, and H. Cheng, HMJAU64093 (Z22061601); **CHINA** • Anhui Province, Hefei City, Feixi County, Zipeng Mountain National Forest Park, 31°43'45"N, 117°00'51"E, 55 m a.s.l., July 22^nd^ 2022, H. Cheng, HMJAU64095 (C22072201); **CHINA** • Hubei Province, Wuhan City, Wuhan Botanical Garden of Chinese Academy of Science, 30°32'56"N, 114°25'07"E, 366 m a.s.l., June 18^th^ 2022, L. Y. Zhu, H. B. Song, and H. Cheng, HMJAU64096 (Z22061809).

#### Notes.

There are three known psathyrelloid species in *Parasola*, namely *Parasolacinnamomescens*, *Parasolaconopilea*, and *ParasolaPsathyrelloides*, and the fourth is the new species *Parasolacrispa*. Different from *Parasolacrispa*, these three species have brown or orange-brown pileus and longer sclerocystidia (in *Parasolaconopilea* 100–300 μm, in *Parasolapsathyrelloid* up to 700 μm and in *Parasolacinnamomescens* 140–395 μm). Additionally, *Parasolaconopilea* has larger basidiospores (in average 14.2 × 7.4 μm) and caulocystidia at top of stipe and its pileus often have metal gloss and sometimes slightly sticky when young from our observation (HMJAU60365, HMJAU60342). Compared with *Parasolacrispa*, *Parasolapsathyrelloides* has finely sulcate-striate up to the center which resembles species in sect.Parasola (Ganga and Manimohan 2019). Differing from *Parasolacrispa*, *Parasolacinnamomescens* processes specialized pileipellis elements that resemble pileocystidia (Khan et al. 2023). Notably, the caulocystidia located at the upper of stipes also serve as a diagnostic feature to distinguish species within this section. Based on our observation, the caulocystidia in *Parasolaconopilea* are predominantly (sub)lageniform or subglobose in shape and relatively large size, averaging 44 × 15 μm (as showed in Fig. 25a–c). Interestingly, *Parasolacrispa* appears to occupy a transitional position between *Parasolaconopilea* and *Parasolapsathyrelloid*, as indicated by the degree of smoothness of their pileus. The detailed comparisons of the aforementioned *Parasola* species are presented in Table 2.

In addition to species within sect.Conopileae, *Parasolacrispa* is often macroscopically confused with *Psathyrellaamarescens* Arnolds and *Psathyrellacorrugis* (Pers.:Fr.) Konr. & Maubl due to their shared similar morphological features, such as conical, lacteous to pale brown-gray pileus with slight folding and slender stipes. However, stipes of these two *Psathyrella* species are covered with small, white, evanescent fibrils, particularly when young, and these two species possess fusiform cheilocystidia and pleurocystidia (Yan 2018; Friebes and Melzer 2009). However, these features are absent in *Parasolacrispa*.

**Figure 6.**
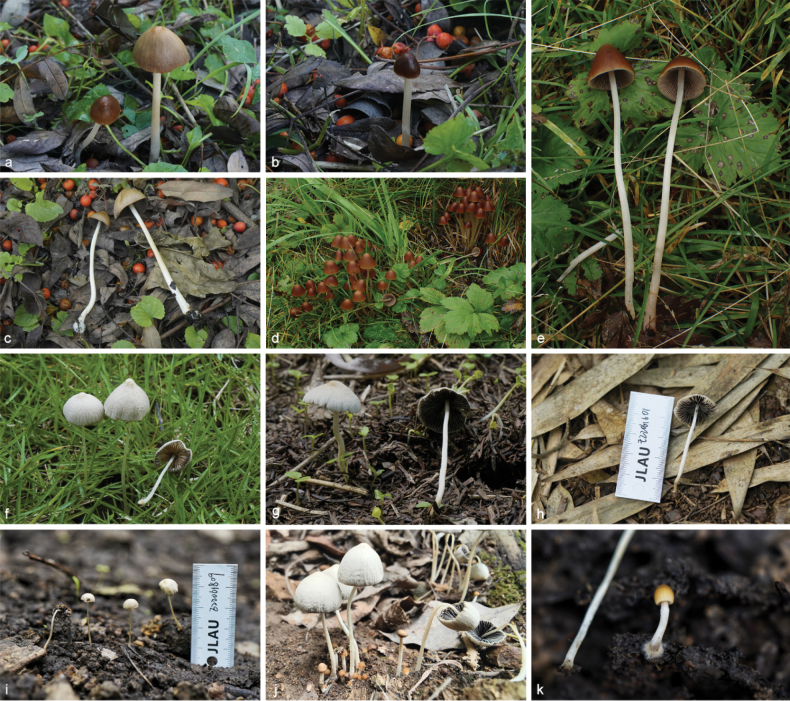
Basidiomata of species in sect.Conopileae. **a–e***Parasolaconopilea*: **a–c** HMJAU60340, **d, e** HMJAU60339; **f–k***Parasolacrispa*: **f** HMJAU60339, **g** HMJAU60340, **h** HMJAU60342, **i** HMJAU64093, **j** HMJAU60341, **k** HMJAU64096.

**Figure 7.**
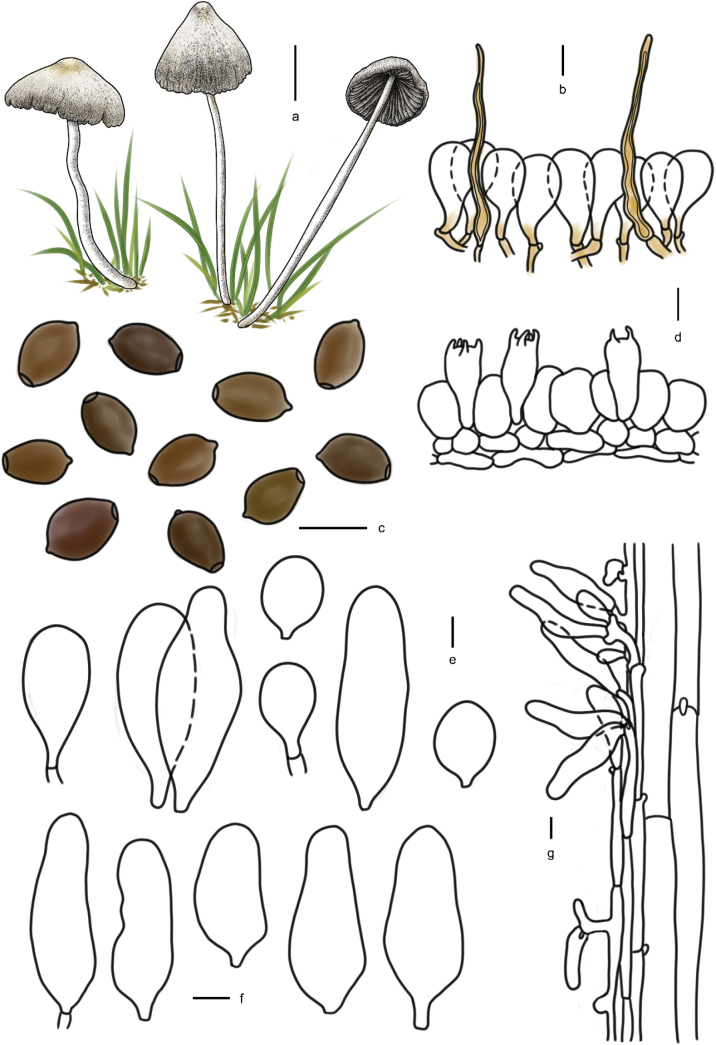
Basidiomata and microscopic structures of *Parasolacrispa*: **a** basidiomata (HMJAU60339, HMJAU60340, HMJAU60341); **b** pileipellis (HMJAU60339); **c** basidiospores (HMJAU60339, HMJAU64093, HMJAU64096); **d** basidia (HMJAU60339); **e** cheilocystidia (HMJAU60339, HMJAU60341); **f** pleurocystidia (HMJAU60339, HMJAU60341); **g** stipitipellis (HMJAU60339). Scale bars: 1 cm (**a**); 10 μm (**b–g**).

**Table 2.** Comparative characters of *Parasolacrispa* and related taxa.

Species	Pileus size	Pileus color	Sulcate-striate at pileus	Basidiospores size	Specialized pileipellis cells	Sclerocystidia size	Caulocystidia shape and size
*Parasolacinnamomescens*	5–16 mm	dull orange to yellow to light gray	absent	9.5–13 × 6–8 µm	present	140–395 × 2–6 µm	32–60 × 12–17 µm, lageniform or clavate, fusiform, or fusoid ventricose
*Parasolaconopilea*	20–50 mm	dark reddish brown	absent	14–18 × 6–8 µm	absent	100–300 μm in length	30–85 × 14–30 µm, mainly (sub)lageniform
*Parasolacrispa*	10–18 mm	lacteous to sordid yellow	absent	9–11 × 6–7 × 7–8 μm	absent	28–159 × 4–6 μm	30–53 × 11–16 μm, mainly cylindrical or broad lageniform
*Parasolapsathyrelloides*	2–20 mm	light brown to brownish orange	present	8–12 × 7–9 × 6–8 µm	absent	300–700 μm in length	–

Note: “–” indicates that the information is not recorded.

### ﻿ Parasola sect. Parasola

Taxon classificationFungiAgaricalesPsathyrellaceae

Redhead, Vilgalys & Hopple

FA3C6FC8-1F6E-5AEF-AC76-0604D5EAE1A9

#### Description.

Basidiomata small-sized, mainly terrestrial, sometimes fimicolous or lignicolous, withering or partial deliquescent at age. Pileus almost flattened cracking into plication when mature, veil absent. Lamellae free, mostly remote from stipe, withering. Stipes hollow, most glabrous, occasionally with flour scales. Basidiospores subglobose, ellipsoid, ovoid, rounded subtriangular, rhombic, subpentangular, hexagonal in front view, ellipsoid or lentiform in side view, germ pore central or eccentric. Basidia dimorphic, regularly 4-spored. Lamellae trama regular, colorless or in brown hue. Lamellae margin infertile, cheilocystidia abundant, subglobose, utriform, ellipsoid, sublageniform or subcylindrical. Pleurocystidia mainly present, subglobose, utriform, sublageniform or subcylindrical. Original pileipellis hymeniform, secondary pileipellis subcutis. Caulocystidia only accumulate at top of stipe forming pseudocollarium. Clamps present and sometimes present pseudoclamps.

### ﻿ Parasola constrictospora

Taxon classificationFungiAgaricalesPsathyrellaceae

T. Bau & L.Y. Zhu sp. nov.

46CC4C4E-BAA7-51C9-ACFA-9DC9953C88C8

MB846521

Figs 8h–i , 9

#### Etymology.

The specific epithet “*constrictospora*” means the constricted ellipsoid shape of basidiospores.

#### Diagnosis.

Pileus sordid yellow to cinnamon with grayish hue when young, orange-brown to chestnut-color at center, brown-gray to gray at margin when age; fine sulcate-striate up to center; context thin, pale gray to gray; basidiospores 10.7–11.4 × 8.8–9.1 × 6.4–6.9 μm, broad ellipsoid to ellipsoid, usually constricted in middle part, dark red-brown to almost black, germ pore central; basidia dimorphic; cheilocystidia globose, subglobose, utriform, ellipsoid or broad lageniform; pleurocystidia similar to cheilocystidia; sclerocystidia present.

#### Type.

**CHINA** • Jiangsu Province, Nanjing City, North Garden of Nanjing Botanical Garden Mem. Sun Yat-sen, 32°05'55"N, 118°83'61"E, 207 m a.s.l., August 28^th^ 2021, H. T. Luo, LHT01 (holotype HMJAU60336).

#### Description.

Pileus 8–12 × 5–10 mm when still closed, 12–19 mm when mature, at first conical to convex, finally become flat, with glabrous disc at center, sometimes slightly depresses; dry; sordid yellow to cinnamon with grayish hue when young, orange-brown to chestnut-color at center, brown-gray to gray at margin when aged; fine sulcate-striate up to center. Context thin, pale gray to gray, odor and taste not distinctive. Lamellae crowded, almost free to free, 1–2 mm in wide, L = 30–41, I = 0 or 1, initially pale gray then become gray to dark gray when mature, more pale at margin; hardly deliquescent. Stipe 49–61 × 2–3 mm, cylindrical, hollow, almost equal, pale gray and with somewhat brown hue at base, subglabrous with sparse tiny hairs, clavate. Spore print without recording.

Basidiospores[42, 3, 2] (9.6–)10.7–11.4(–13.2) × (7.6–)8.8–9.1(–9.8) × (6.1–)6.4–6.9(–7.5) μm, Q_1_ = 1.07–1.42, Q_2_ = 1.36–1.59, av. Q_1_ = 1.24, av. Q_2_ = 1.47; broad ellipsoid to ellipsoid, or mitriform, usually constricted in middle part, with apical papilla and convex base in front view, slightly flattened, ellipsoid in side view; smooth, dark red-brown to almost black; inamyloid; germ pore central, 2.5–3.1 μm wide. Basidia dimorphic, 20–32 × 11–12 μm, sterigma 3–6 μm in length, clavate, hyaline, 4- or 2-spored, surrounded with 4–6 pseudoparaphyses; subhymenium composed of ellipsoid, oblong or cylindrical elements, 18–28 × 10–18 μm. Cheilocystidia 20–70 × 11–26 μm, globose, subglobose, utriform, ellipsoid or broad lageniform, smooth, colorless, thin-walled. Pleurocystidia 15–60 × 11–25 μm, similar to cheilocystidia. Lamella trama regular, 3–13 μm in diam, hyaline, colorless, thin-walled. Pileipellis a hymeniderm at yellow-brown sulcate, made up of sphaeropedunculate cells, 19–43 × 11–21 μm, hyaline, with brown hue at base in most cases; other part of pileus with brown-gray hue a cutis, made up of hyaline, brown to brown-gray, 3–8 μm in diam; pileus trama hyphae densely interwoven, thin-walled, hyaline, yellow-brown to brown, 4–17 μm wide; sclerocystidia 29–161 × 6–9 μm, yellow-brown, thick-walled, wall 1.4–2.8 μm in thickness. Caulopellis hyphae parallel, 4–9 μm wide, hyaline, thin-walled, sometimes diverticulate; caulocystidia unseen, however, clustered tiny hairs at stipe pellis; hyphae of stipe trama 9–21 μm wide, colorless, thin-walled. Clamp connection present at all tissues.

#### Ecology.

Solitary, subfasciculate, or in small groups, grow on wood chips. Fruiting in August. Only known from type locality.

#### Other specimens examined.

**CHINA** • Jiangsu Province, Nanjing City, North Garden of Nanjing Botanical Garden Mem. Sun Yat-sen, 32°05'55"N, 118°83'61"E, 207 m a.s.l., August 28^th^ 2021, W. J. Li, HMJAU60338 (LWJ03).

#### Note.

Differed from other species with sclerocystidia, *Parasolaconstrictospora* has distinct brown pileus, constricted ellipsoid basidiospores and lignicolous habitat. The sparse tiny hairs on stipitipellis might be the veil residue. This species may also distribute in Pakistan based on phylogenetic results (in Figs 1–4Parasolaaff.auricoma LAH-SHP-7 and Parasolaaff.auricoma LAH-SHP-11).

**Figure 8.**
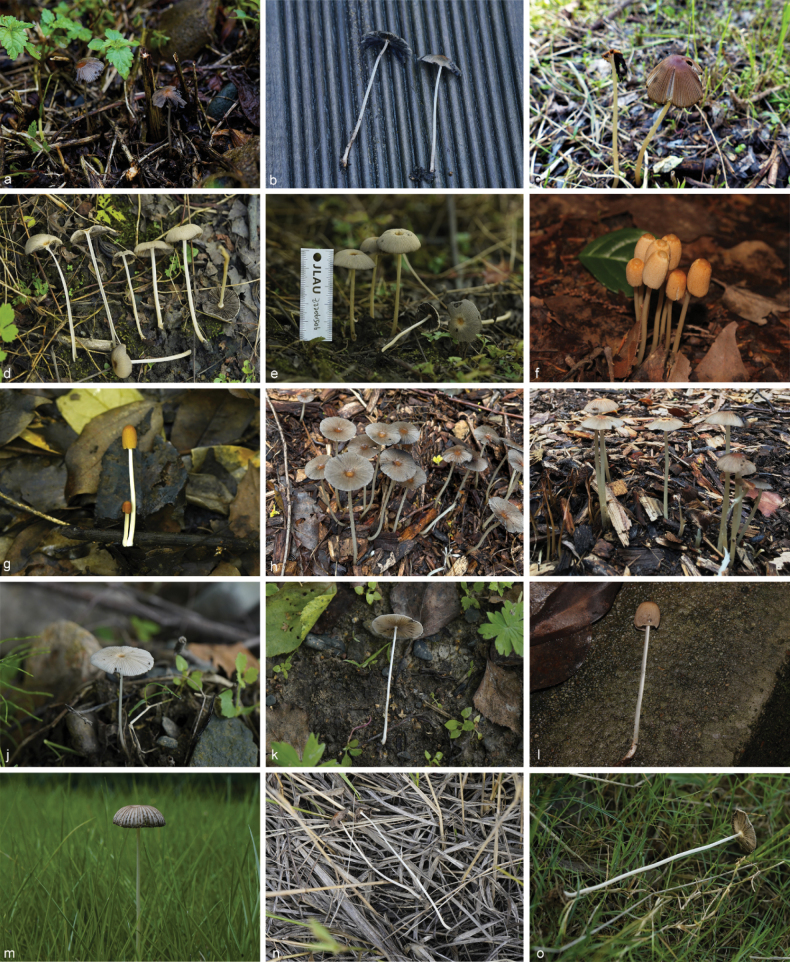
Basidiomata of species with sclerocystidia in sect.Parasola: **a–c***Parasolaauricoma*: **a, b**HMJAU 64094, **c** HMJAU46369; **d–g***Parasolagrisella*: **d** HMJAU64097, **e** HMJAU64098, **f** HMJAU60094, **g** HMJAU60095; **h–i***Parasolaconstrictospora*: **h** HMJAU60336, **i** HMJAU60337; **j–l***Parasolasetulosa*: **j, k** HMJAU60096, **l** HMJAU60097; **m–o***Parasolamalakandensis*: **m** HMJAU60360, **n** HMJAU64091, **o** HMJAU64090.

**Figure 9.**
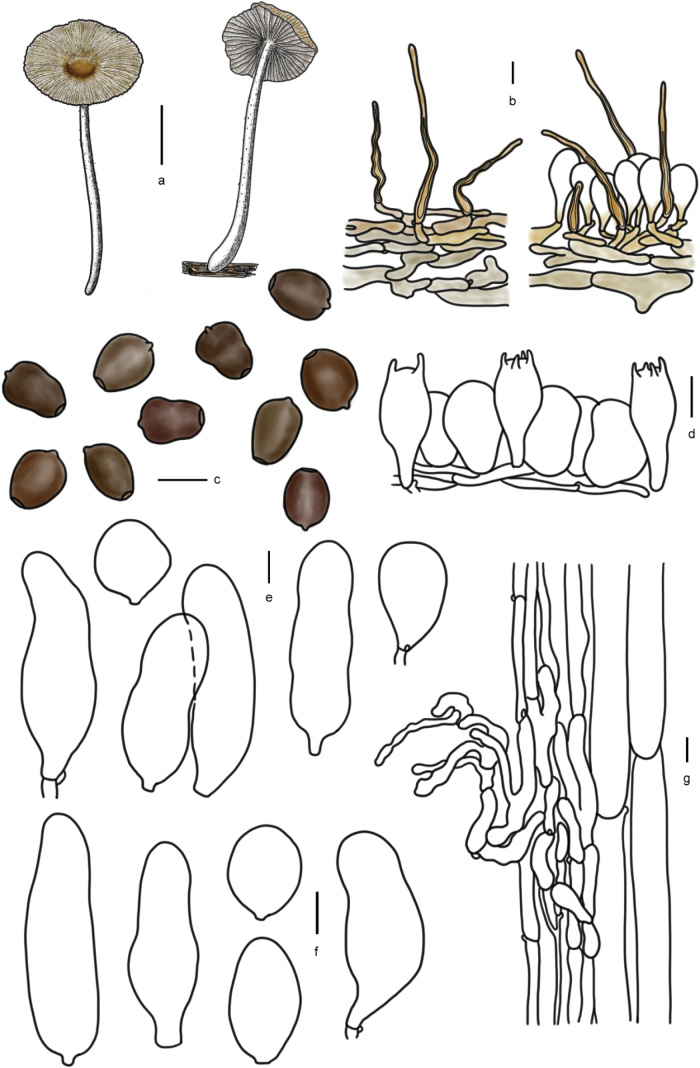
Basidiomata and microscopic structures of *P.constrictospora*: **a** basidiomata (HMJAU60336, HMJAU60337); **b** pileipellis (HMJAU60336); **c** basidiospores (HMJAU60336, HMJAU60337); **d** basidia (HMJAU60336); **e** cheilocystidia (HMJAU60336); **f** pleurocystidia (HMJAU60336); **g** stipitipellis (HMJAU60336). Scale bars: 1 cm (**a**); 10 μm (**b–g**).

**Table 3.** Comparative characters of species with sclerocystidia in sect.Parasola.

Species	Pileus size and color	Growth patterns	Basidiospores size and shape	Basidiospores constriction present or absent	Cheilocystidia Shape	Pleutocystidia present or absent	Sclerocystidia wall thickness	Substrate
*Parasolaauricoma*	10–60 mm, chestnut-color	solitary or subfasciculate	10.0–14.5 × 6.0–8.0 μm, ellipsoid to oblong	absent	utriform, sublageniform, subcylindrical or ellipsoid	present	0.9–1.4 μm	soil, wood chips, and grassy places
*Parasolaconstrictospora*	12–19 mm, sordid yellow to cinnamon	solitary, subfasciculate, or in small groups	10.7–11.4 × 8.8–9.1 × 6.4–6.9 μm, broad ellipsoid to ellipsoid, or mitriform	present	globose, subglobose, utriform, ellipsoid or broad lageniform	present	1.4–3.2 μm	wood chips
*Parasolagrisella*	14–27 mm, pearly gray	fasciculate or in small groups	9.3–10.1 × 7.7–8.0 × 6.3–6.5 μm, broad ellipsoid	absent	subglobose, utriform, ellipsoid or broad lageniform	present	1.5–2.5 μm	humus layer or soil near trumps
*Parasolamalakandensis*	8–12 mm, light ochreous or brown	solitary or in small groups	14.9–16.3 × 10.6–11.4 × 10.4–11.2 μm, ellipsoid	absent	ellipsoid or sublageniform	rare	1.4–2.8 μm	grassy land
*Parasolasetulosa*	10–25 mm, pale gray	solitary	9.7–10.4 × 6.9–8.1 μm, ellipsoid	absent	lageniform of clavate	present	2.8–4.4 μm	soil

### ﻿ Parasola grisella

Taxon classificationFungiAgaricalesPsathyrellaceae

T. Bau & L.Y. Zhu sp. nov.

C6B001E2-835D-5367-9012-5E72E6EB8902

MB846522

Figs 8d–g , 10

#### Diagnosis.

Basidiomata relatively large. Pileus pearly white to pale gray at margin and yellow-brown at the center; with annular zone at the junction of the lamellae and stipe, basidiospores 9.3–10.1 × 7.7–8.0 × 6.3–6.5 μm, broad ellipsoid in front view, brown to dark brown; basidia dimorphic, 4-spored; cheilocystidia 19–77 × 8–19 μm, subglobose, utriform, ellipsoid or broad lageniform; pleurocystidia 55–80 × 19–25 μm, (sub)cylindrical or lageniform; pileipellis a hymeniderm mixed with sphaeropedunculate and clavate cells; sclerocystidia present; clustered tiny hairs at stipipellis.

#### Etymology.

The specific epithet “*grisella*” refers the distinct pearly gray color of pileus when mature.

#### Type.

CHINA • Anhui Province, Chizhou City, Shitai County, Guniujiang National Nature Reserve, 30°01'39"N, 117°52'89"E, 164 m a.s.l., June 15^th^ 2022, L. Y. Zhu, H. B. Song and H. Cheng, Z22061506 (HMJAU60338, holotype).

#### Description.

Basidiomata small to medium-sized. Pileus 5–9 × 5–8 mm when still closed, 14–27 mm when mature, at first conical to subglobose, finally become flat, with glabrous disc at center; dry; yellow brown to red brown at first, then fade from edge of pileus, finally become pearly gray except sordid yellow to yellow brown at center; fine sulcate-striate up to center. Context thin, cream to pale gray, odor and taste not distinctive. Lamellae crowded, free, with annular zone at the junction of the lamellae and stipe, 1–2 mm in wide, L = 35–43, I = 0–2, initially pale gray then become gray with olive-green hue, dark gray when mature, more pale at margin; hardly deliquescent. Stipe 43–64 × 2–3 mm, cylindrical, hollow, almost equal, cream to pale gray, subglabrous with sparse tiny hairs, clavate. Spore print not recorded.

Basidiospores[63, 5, 3] (7.7–)9.3–10.1(–10.5) × (6.8–)7.7–8.0(–8.4) × (5.8–)6.3–6.5(–6.7) μm, Q_1_ = 1.18–1.28, Q_2_ = 1.26–1.56, av. Q_1_ = 1.21, av. Q_2_ = 1.40; broad ellipsoid, occasionally with 5- or 6-rounded angles, with apical papilla and convex base in front view, slightly flattened, narrow ellipsoid to ellipsoid in side view; smooth, brown to dark brown in H_2_O and olive-brown in 5% KOH; inamyloid; germ pore central, 1.7–2.7 μm wide. Basidia bimorphic, 17–32 × 8–11 μm, sterigma 3–6 μm in length, clavate, sometimes constricted in middle part, hyaline, 4-spored, surrounded with 3–5 pseudoparaphyses; subhymenium composed of ellipsoid, oblong or cylindrical elements, 7–20 × 8–11 μm. Cheilocystidia 19–77 × 8–19 μm, subglobose, utriform, ellipsoid or broad lageniform, smooth, colorless, thin-walled. Pleurocystidia 55–80 × 19–25 μm, (sub)cylindrical or lageniform. Lamellar trama regular, 8–16 μm in diam, hyaline, colorless, thin-walled. Pileipellis a hymeniderm at sulcate, made up of sphaeropedunculate and clavate cells, 29–43 × 9–22 μm, hyaline, with brown hue at base in most cases; other part of pileus a cutis, made up of hyaline, colorless or slightly yellow hyphae, 3–8 μm in diam; pileus trama hyphae densely interwoven, thin-walled, hyaline, yellow-brown to brown, 6–17 μm wide; sclerocystidia 29–181 × 5–11 μm, yellow-brown, thick-walled, wall 2.5–3.2 μm in thickness. Stipitipellis hyphae parallel, 4–10 μm wide, hyaline, thin-walled, sometimes diverticulate; caulocystidia unseen, while clustered terminal cells of the stipitipellis protrude from the surface; hyphae of stipe trama 9–16 μm wide, colorless, thin-walled. Clamp connections present.

#### Ecology.

Subfasciculate or in small groups, grow on clayey soil, humus layer or adnate to trumps in broad-leaved tree with *Liriodendronchinense*, *Liquidambarformosana*, *Osmanthusfragrans* and *Camphoraofficinarum*. Fruiting in May to July. Known from East and Central China regions.

#### Other specimens examined.

**CHINA** • Same location with holotype, June 15^th^ 2022, L. Y. Zhu, H. B. Song and H. Cheng, HMJAU64098 (Z22061508); **CHINA** • Jiangsu Province, Nanjing City, North Garden of Nanjing Botanical Garden Mem. Sun Yat-sen, 32°05'55"N, 118°83'61"E, 191 m a.s.l., May 16^th^ 2021, Z. H. Zhang, HMJAU64097 (ZZH516); **CHINA** • Hubei Province, Wuhan City, lawn beside Art College of Wuhan University, No. 421 Luojiashan Road, 30°32'27"N,114°21'45"E, 39 m a.s.l., May 29^th^ 2022, M. H. Tang, HMJAU60093 (355); **CHINA** • Hubei Province, Wuhan City, Lover Slope of Wuhan University, 30°32'27"N, 114°21'31"E, 51 m a.s.l., June 2^nd^ 2022, M. H. Tang, HMJAU60094 (390); **CHINA** • Hunan Province, Changsha City, Yuelu Mountain, 28°11'01"N, 112°55'59"E, 218 m a.s.l., July 8^th^ 2022, L. Y. Zhu, H. B. Song and H. Cheng, HMJAU60095 (Z22070833).

#### Note.

This species is a sister to *Parasolaauricoma*, which differs from the former in having a yellowish-brown pileus at maturity, ellipsoid to elongated ellipsoid basidiospores in frontal view (Q_1_ = 1.35–2.05), and sclerocystidia with wall thickness of 0.9–1.4 μm (Uljé 2005; Huang 2019). Geographically, the known distribution range of *Parasolagrisella* is the middle and lower reaches of the Yangtze River in China, which belongs to the subtropical region, while the suitable habitat of *Parasolaauricoma* is concentrated in the northern temperate zones of Asia and Europe.

*Parasolagrisella* is macroscopically similar to *Parasolasetulosa* (Berk. & Broome) Redhead, Vilgalys & Hopple, as both species exhibit grayish-white pilei at maturity. However, the latter is usually solitary, lacks an annular zone at the junction of the lamellae and stipe, has ellipsoid basidiospores in frontal view, lageniform or clavate cheilocystidia, and sclerocystidia with wall thickness of 2.8–4.4 μm (Cho et al. 2018; Huang Mei 2019). Additionally, *Parasolaconstrictospora*, which is also distributed in the middle and lower reaches of the Yangtze River, differs from this species by its constricted basidiospores in front view.

**Figure 10.**
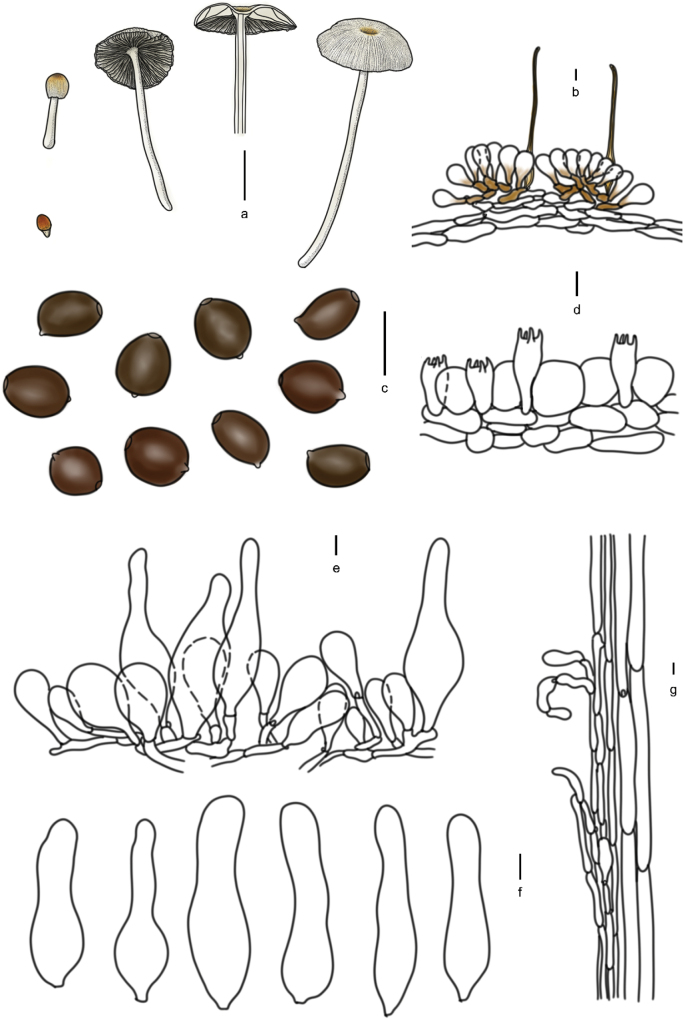
Basidiomata and microscopic structures of *Parasolagrisella*: **a** basidiomata (HMJAU60338, HMJAU64098, HMJAU60095); **b** pileipellis (HMJAU60338); **c** basidiospores (HMJAU60338, HMJAU64097, HMJAU64095, HMJAU60095, HMJAU60093); **d** basidia (HMJAU60338); **e** cheilocystidia (HMJAU60338, HMJAU64097); **f** pleurocystidia (HMJAU60338, HMJAU64097); **g** stipitipellis (HMJAU60338, HMJAU64097). Scale bars: 1 cm (**a**); 10 μm (**b–g**).

### ﻿ Parasola malakandensis

Taxon classificationFungiAgaricalesPsathyrellaceae

S. Hussain, Afshan & H. Ahmad, in Hussain, Afshan, Ahmad, Khalid & Niazi, Mycoscience 58: 72 (2017).

6DE436BE-CA81-5A8B-B7A8-540F80167BA2

Figs 8m–o , 11

#### Description.

Pileus 7–10 × 4–8 mm when still closed, 8–12 mm when mature, at first ovoid or ellipsoid, expanded pileus conical to flattened; dry; sordid yellow, ochreous to red brown at center, gray or pale gray at margin, sometimes with slightly brown hue; sulcate-striate up to center. Context extremely thin, almost unseen in membrane part of pileus, white to pale gray, odor and taste not distinctive. Lamellae crowded, free and remote from stipe, with a circular empty space which is visible around the apex of the stipe, 1–2 mm in wide, L = 35–41, I = 1–2, first white to beige, then become purple gray to dark gray, hardly deliquescent with age. Stipe 20–51 × 1–2 mm, cylindrical, hollow, equal or attenuate towards the apex, white, glabrous; with white tomentose at base. Spore print not recorded.

Basidiospores [40, 2, 1] (14.6–)14.9–16.3(–17.1) × (9.3–)10.6–11.4(–12.6) × (10.1–)10.4–11.2(–12.2) μm, Q_1_ = 1.24–1.59, Q_2_ = 1.31–1.68, av. Q_1_ = 1.39, av. Q_2_ = 1.46; ellipsoid, with apical papilla and convex base in front view, ellipsoid in side view; smooth, dark brown to almost black, with olive-brown oil droplet; inamyloid; germ pore central, 2.6–4.9 μm wide. Basidia dimorphic, 22–43 × 9–12 μm (in average 33 × 15 μm), sterigma 3–7 μm, clavate, sometimes constricted in middle part, hyaline, 4-spored, surrounded with 5–8 pseudoparaphyses; subhymenium composed of subglobose, ellipsoid, oblong or cylindrical elements. Cheilocystidia 34–53 × 23–34 μm, abundant, ellipsoid or sublageniform, smooth, colorless, thin-walled. Pleurocystidia rare, if present, (sub) cylindrical, 56–81 × 15–22 μm, colorless, thin-walled. Lamella trama regular, 3–9 μm wide, hyaline, colorless, thin-walled. Pileipellis a hymeniderm, made up of sphaeropedunculate and clavate cells, 21–44 × 9–23 μm, hyaline, most with brown hue at base; pileus trama hyphae densely interwoven, thin-walled or slightly thick-walled, hyaline, yellow-brown to brown, 3–7 μm wide; sclerocystidia present, 34–135 × 4–7 μm, yellow-brown to brown, thick-walled, wall 1.4–2.8 μm in thickness. Stipipellis hyphae parallel, 2–6 μm wide, hyaline, thin-walled, sometimes with short branches; hyphae of stipe trama 8–18 μm wide, colorless, thin-walled; caulocystidia unseen. Clamp connection abundant.

#### Ecology.

Solitary or in small groups, grow on soil in grassy places. July. Recorded with certainty from Pakistan, Hungary and United States (Hussain et al. 2016a); other reports also from South Africa and Tanzania (https://www.gbif.org/); known from Inner Mongolia Autonomous Region, Zhejiang Province, Anhui Province and Hubei Province in China so far. Fruiting in May to November.

#### Specimens examined.

**CHINA** • Inner Mongolia Autonomous Region, Tongliao City, Horqin Left Back Banner, Muritu Town, 43°42'36"N, 122°97'13"E, 186 m a.s.l., August 22^nd^ 2022, T. Bau, L. Y. Zhu and S. E. Wang, HMJAU64091 (Z22082220); **CHINA** • Zhejiang Province, Hangzhou City, Zijingang Campus of Zhejiang University, 30°30'22"N, 120°08'67"E, 94 m a.s.l., July 5^th^ 2021, T. Bau, L. Y. Zhu and W. F. Lin, HMJAU60360 (Z21070507); **CHINA** • Anhui Province, Chizhou City, Chizhou High Speed Railway Station, 30°62'69"N, 117°52'84"E, 26 m a.s.l., L. Y. Zhu, H. B. Song and H. Cheng, June 14^th^ 2022, HMJAU64090 (Z22061403); **CHINA** • Hubei Province, Wuhan City, Luojia Square of Wuhan University, 30°32'15"N, 114°21'21"E, 34 m a.s.l., November 5^th^ 2021, M. H. Tang, HMJAU64092 (TMH115).

#### Notes.

*Parasolamalakandensis* is usually macro-morphologically confused with *Parasolaauricoma* and *Parasolaconstrictospora*. Distinct from *Parasolamalakandensis*, the basidiospores of *Parasolaauricoma* are smaller (10.0–14.5 × 6.0–8.0 μm) and ellipsoid to oblong in front view (Uljé 2005; Huang 2019). Compared to *Parasolamalakandensis*, the basidiospores of *Parasolaconstrictospora* are constricted in front view and this species is lignicolous.

**Figure 11.**
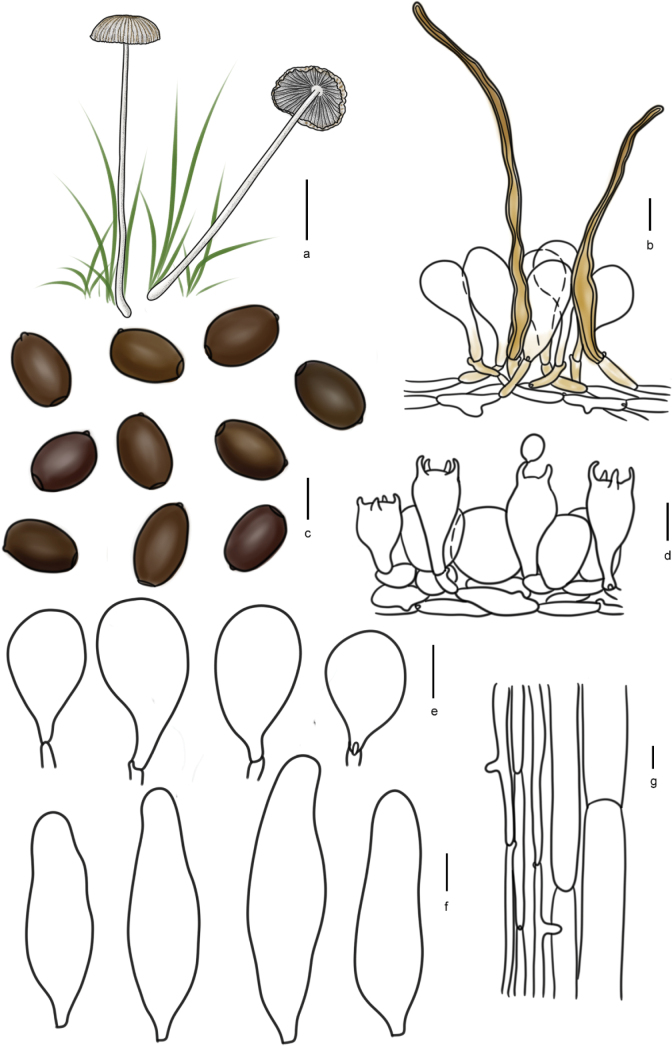
Basidiomata and microscopic structures of *Parasolamalakandensis* (HMJAU60360, HMJAU64090, HMJAU64091, HMJAU64092): **a** basidiomata (HMJAU60360, HMJAU64090); **b** pileipellis (HMJAU60360); **c** basidiospores (HMJAU60360); **d** basidia (HMJAU64090); **e** cheilocystidia (HMJAU60360); **f** pleurocystidia; **g** stipitipellis. Scale bars: 1 cm (**a**); 10 μm (**b–g**).

### ﻿ Parasola tenuissima

Taxon classificationFungiAgaricalesPsathyrellaceae

T. Bau & L.Y. Zhu sp. nov.

B9B7E464-5C90-55C4-9A3E-5E49E73E2C8A

MB846523

Figs 12a–c , 13

#### Diagnosis.

Basidiomata tiny-sized. Pileus less than 10 mm in diameter, orange-brown in center, often with a conspicuous depressed disc; stipe white to pale gray at upper part, brown to sandy beige at lower part, sometimes translucent; basidiospores 9.7–10.1 × 7.9–8.2 × 6.1–6.6 μm, most in ovoid, ellipsoid or limoniform in front view, flattened, ellipsoid to narrow ellipsoid in side view, dark olive-brown to almost black; basidia dimorphic, 4-spored; lamella trama dark olive-brown; cheilocystidia 18–49 × 11–24 μm, utriform, ellipsoid or fusiform; pleurocystidia 38–77 × 16–26 μm, sublageniform or (sub)cylindrical; pileipellis a hymeniderm at yellow-brown sulcate, mainly made up of ellipsoid or clavate cells, sometimes subglobose or utriform with short pedicels; sclerocystidia absent.

#### Etymology.

The epithet “*tenuissima*” refers to the slender basidiocarps of this species.

#### Type.

**CHINA** • Jilin Province, Changchun City, Nanhu Park, 43°85'25"N, 125°29'98"E, 218 m a.s.l., on humus layer of broad-leaved tree, August 17^th^ 2022, S. E. Wang and L. Y. Zhu, HMJAU64084 (E2208213, holotype).

#### Description.

Basidiomata tiny-sized. Pileus 4–6 × 7–10 mm when still closed, 8–10 mm when mature, at first ovoid or ellipsoid, finally almost flattened, often with a conspicuous depressed disc at center; dry; cream to light yellow-brown at margin and orange-brown in center when young, pale gray but always with brown hue at age, sometimes with water-soaking texture; sulcate-striate almost up to center. Context extremely thin, pale brown or almost unseen, odor and taste not distinctive. Lamellae medium crowded, free and remote from stipe by pseudocollarium, 1 mm in wide, L = 27–32, I = 0 or 1, first white to beige, pale gray when expanded; hardly deliquescent with age. Stipe 35–51 × 1–2 mm, cylindrical, hollow, equal or attenuate towards the apex, slender, white to pale gray at upper part, brown to sandy beige at lower part, sometimes translucent, glabrous. Spore print not recorded.

Basidiospores [50, 4, 3] (8.9–)9.7–10.1(–12.2) × (7.4–)7.9–8.2(–9.2) × (5.9–)6.1–6.6(–6.7) μm, Q_1_ = 1.10–1.47, Q_2_ = 1.41–1.75, av. Q_1_ = 1.25, av. Q_2_ = 1.53; most in ovoid, ellipsoid or limoniform in front view, with apical papilla and convex base, flattened, ellipsoid to oblong in side view; smooth, dark olive-brown, dark red-brown to almost black, with yellow-brown oil droplet; inamyloid; germ pore eccentric, 1.5–2.5 μm wide. Basidia dimorphic, 17–36 × 8–13 μm, sterigma 4–7 μm, clavate, occasionally constricted in middle part, hyaline, 4-spored, surrounded with 4–6 pseudoparaphyses; obvious subhymenium unseen. Cheilocystidia abundant, 18–49 × 11–24 μm, utriform, ellipsoid or fusiform, smooth, colorless, thin-walled. Pleurocystidia 38–77 × 16–26 μm, sublageniform or (sub)cylindrical, colorless, thin-walled. Lamellar trama regular, 4–7 μm wide, hyaline, dark olive-brown, thin-walled. Pileipellis a hymeniderm at yellow-brown sulcate, mainly made up of clavate cells, sometimes subglobose or utriform with short pedicels, 16–42 × 11–23 μm, hyaline, with brown hue at base; other part of pileus with gray hue a cutis, made up of hyaline, colorless to light brown, 2–3 μm; pileus trama hyphae slightly interwoven, thin-walled, hyaline, yellow-brown to yellow, 4–7 μm wide. Sclerocystidia absent. Caulopellis hyphae parallel, 2–7 μm wide, hyaline, thin-walled, often short-diverticulate; hyphae of stipe trama 10–16 μm wide, colorless, thin-walled; caulocystidia unseen. Clamp connection and pseudoclamp present.

#### Ecology.

Solitary, subfasciculate, or in small groups grow on humus layer in broad-leaved forest with *Quercusmongolica*, *Malusbaccata*, *Acer* and *Ulmus* or under bush of *Chimonanthusnitens*. Fruiting in August to September. Only known from northeast China.

#### Other specimens examined.

**CHINA** • Jilin Province, Jilin City, Jiaohe City, Qianjin Forest Farm, 43°54'35.8"N, 127°39'11.8"E, 343 m a.s.l., July 23^rd^ 2022, L. Y. Zhu, H. B. Song and X. Wang, HMJAU60098 (Z22072316); **CHINA** • Jilin Province, Yanbian Korean Autonomous Prefecture, Antu County, Erdaobaihe Town, Chinese Mermanser Park, 42°41'73.7"N, 128°11'40.5"E, 719 m a.s.l., August 1^st^ 2022, L. Y. Zhu, HMJAU64085 (Z22080101); **CHINA** • Jilin Province, Changchun City, Jingyuetan National Forest Park, 43°79'72.1"N, 125°46'52.3"E, 308 m a.s.l., August 18^th^ 2022, L. Y. Zhu, HMJAU64086 (Z22081803).

#### Notes.

Phylogenetically, *Parasolatenuissima* and *Parasolachowii* are closely related. Distinguishing itself from the former, *Parasolachowii* exhibits slightly larger basidiocarps, with expanded pileus reaching up to 1.4 cm in diameter. Moreover, the lamellae are densely arranged with L = 35–41, and the basidiospores appear subglobose to broadly ellipsoid or ovoid in front view.

Known species with brown stipes in *Parasola* until now are only *Parasolaplicatilis-similis* L. Nagy, Szarkándi & Dima, *Parasolaparvula* Ganga & Manimohan and *Parasolatenuissima*, however, *Parasolaplicatilis-similis* has relatively larger basidiocarps whose pileus are up to 15–45 mm when expanded (Szarkándi et al. 2017). Differed from *Parasolatenuissima*, *Parasolaparvula* has pileus with purple hue and sparser lamellae (L = 19–20) (Ganga and Manimohan 2018).

**Figure 12.**
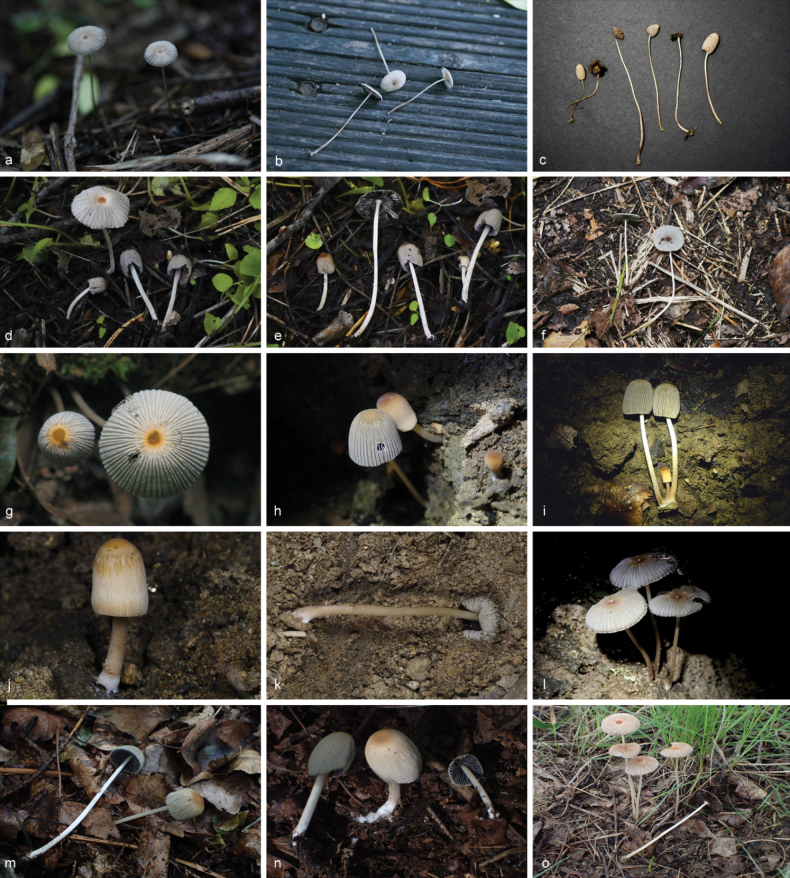
Basidiomata of species in Clade Plicatilis and Clade Misera of sect.Parasola. **a–c***Parasolatenuissima*: **a** HMJAU64085, **b** HMJAU64086, **c** HMJAU64084; **d–f***Parasolachowii*: **d** HMJAU60358, **e** HMJAU60374, **f** HMJAU64099; **g–l***Parasolaneoplicatilis*: **g, h** HMJAU64087, **i** HMJAU64088, **j, k** HMJAU60084, **l** HMJAU64089; **m, n***Parasolaplicatilis*: **m** HMJAU60367, **n** HMJAU60366; **o***Parasolamegasperma* HMJAU60099.

**Figure 13.**
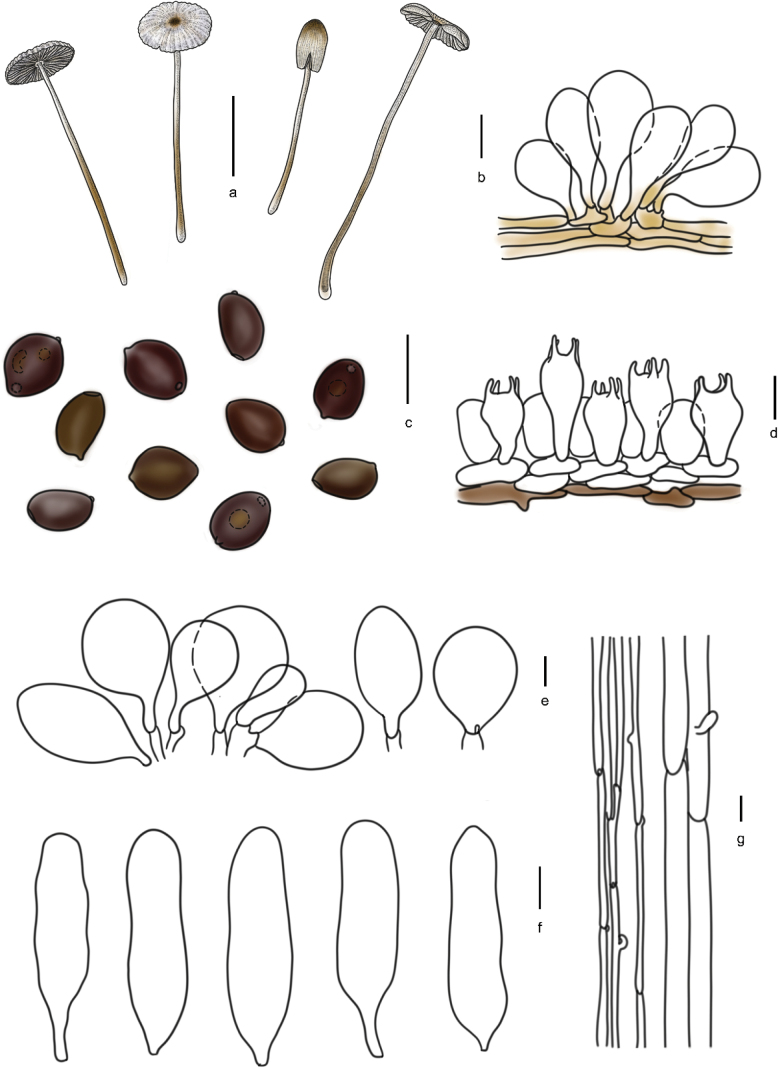
Basidiomata and microscopic structures of *Parasolatenuissima* (HMJAU60098, HMJAU64084, HMJAU64085, HMJAU64086): **a** basidiomata, **b** pileipellis, **c** basidiospores, **d** basidia, **e** cheilocystidia, **f** pleurocystidia, **g** stipitipellis. Scale bars: 1 cm (**a**); 10 μm (**b–g**).

### ﻿ Parasola chowii

Taxon classificationFungiAgaricalesPsathyrellaceae

T. Bau & L.Y. Zhu sp. nov.

3708EAE6-EB3E-5E74-B1AF-AE3F41A352C8

MB846524

Figs 12d–f , 14

#### Diagnosis.

Pileus sordid yellow, ochreous to dark red-brown at center and gray or pale gray at margin when mature; basidiospores 11.4–12.0 × 10.0–10.4 × 8.4–9.4 μm, ovoid or broadly ellipsoid, sometimes lemon-shaped in front view, dark red-brown to almost black, germ pore eccentric, 1.7–3.5 μm wide; basidia dimorphic; cheilocystidia 30–75 × 12–27 μm, ellipsoid, sublageniform or constricted-cylindrical; pleurocystidia 34–103 × 10–25 μm, narrow utriform, sublageniform, (sub)cylindrical or clavate.

#### Etymology.

The specific epithet “*chowii*” is a tribute to the Chinese mycologist Chung-Hwang Chow for his contribution to the study of ontogeny of coprinoid fungi.

#### Type.

**CHINA** • Jilin Province, Changchun City, Jingyuetan National Forest Park, 43°78'61"N, 125°44'54"E, 281 m a.s.l., on humus layer of conifer tree, August 26^th^ 2021, L. Y. Zhu, HMJAU60358 (Z21082609, holotype!).

#### Description.

Pileus 6–8 × 9–12 mm when still closed, up to 14 mm when mature, at first ovoid or ellipsoid, expanded pileus conical to flattened; dry; sordid yellow, ochreous to dark red brown at center, gray or pale gray at margin, sometimes with slightly brown hue; sulcate-striate up to center. Context extremely thin, almost unseen in membrane part of pileus, somewhat fleshy and up to 0.8 mm in central disc, white to pale gray, odor and taste not distinctive. Lamellae crowded, free and remote from stipe, with a circular empty space which is visible around the apex of the stipe, 1–2 mm in wide, L = 35–41, I = 1–2, first white to beige, then become purple-gray to dark gray, hardly deliquescent with age. Stipe 20–51 × 1–2 mm, cylindrical, hollow, equal or attenuate towards the apex, white, glabrous; with white tomentose at base. Spore print not recorded.

Basidiospores [60, 4, 3] (10.2–)11.4–12.0(–13.8) × (9.0–)10.0–10.4(–11.4) × (7.8–)8.4–9.4(–9.9) μm, Q_1_ = 1.06–1.28, Q_2_ = 1.22–1.40, av. Q_1_ = 1.14, av. Q_2_ = 1.28; subglobose, ovoid or broadly ellipsoid, sometimes lemon-shaped, with apical papilla and convex base in front view, flattened, broadly ellipsoid to ellipsoid in side view; smooth, dark red-brown to almost black, with yellow-brown oil droplet; inamyloid; germ pore eccentric, 1.7–3.5 μm wide. Basidia dimorphic, 20–41 × 9–12 μm, sterigma 4–7 μm, clavate, sometimes constricted in middle part, hyaline, 4-spored, surrounded with 4–7 pseudoparaphyses; subhymenium composed of subglobose, ellipsoid, oblong or cylindrical elements, 10–28 × 7–19 μm. Cheilocystidia abundant, 30–75 × 12–27 μm, ellipsoid, sublageniform or constricted cylindrical, smooth, colorless, thin-walled. Pleurocystidia 34–103 × 10–25 μm, narrow utriform, sublageniform, (sub)cylindrical or clavate, colorless, thin-walled. Lamella trama regular, 3–5 μm wide, hyaline, colorless, thin-walled. Pileipellis a hymeniderm, made up of sphaeropedunculate cells, 33–49 × 15–36 μm, hyaline, with brown hue at base; pileus trama hyphae densely interwoven, thin-walled, hyaline, yellow brown to brown, 3–5 μm wide. Stipipellis hyphae 3–11 μm in wide, hyaline, thin-walled, colorless, thin-walled; hyphae of stipe trama 8–16 μm wide, colorless, thin-walled; caulocystidia unseen. Clamp connection abundant.

#### Ecology.

Solitary, subfasciculate, or in small groups, grow on coniferous forest or coniferous and broad-leaved mixed forest. Fruiting in August. Currently only known from China

#### Other specimens examined.

**CHINA** • Jinlin Province, Changchun City, Jingyuetan National Forest Park, 43°45'40"N, 125°28'11"E, 312 m a.s.l., August 18^th^ 2022, L.Y. Zhu, HMJAU60374; **CHINA** • Inner Mongolia Autonomous Region, Tongliao City, Daqinggou National Reserve, 42°47'34"N, 112°10'38"E, 247 m a.s.l., August 23^rd^ 2022, T. Bau and L.Y. Zhu, HMJAU64099.

#### Notes.

*Parasolachowii* is morphologically close to *Parasolaplicatilis*, *Parasolaplicatilis-similis*, and *Parasolamegasperma*. *Parasolachowii* distinguishes itself from the abovementioned species by its subglobose, ovoid or broadly ellipsoid spores (Q = 1.06–1.28) and unique habitat of humus layer of forest of *Pinus*. Compared with *Parasolachowii*, basidiospores of *Parasolaplicatilis* are usually narrower, angularly ovoid with five rounded angles or ellipsoid (Q = 1.05–1.80), in *Parasolamegasperma* spores have larger size which could grow up to 17 μm (Uljé and Bas 1988). Apart from *Parasolachowii*, the abovementioned species grow in lawns and other grassy places and *Parasolamegasperma* sometimes also grow on horse dung (Uljé and Bas 1988; Uljé 2005; Szarkándi et al. 2017). Besides, *Parasolaplicatilis-similis* have darker pileus that is honey-colored, ochre-brown when young and still grayish upon ageing with a pale ochre-brown button when mature (Szarkándi et al. 2017).

**Figure 14.**
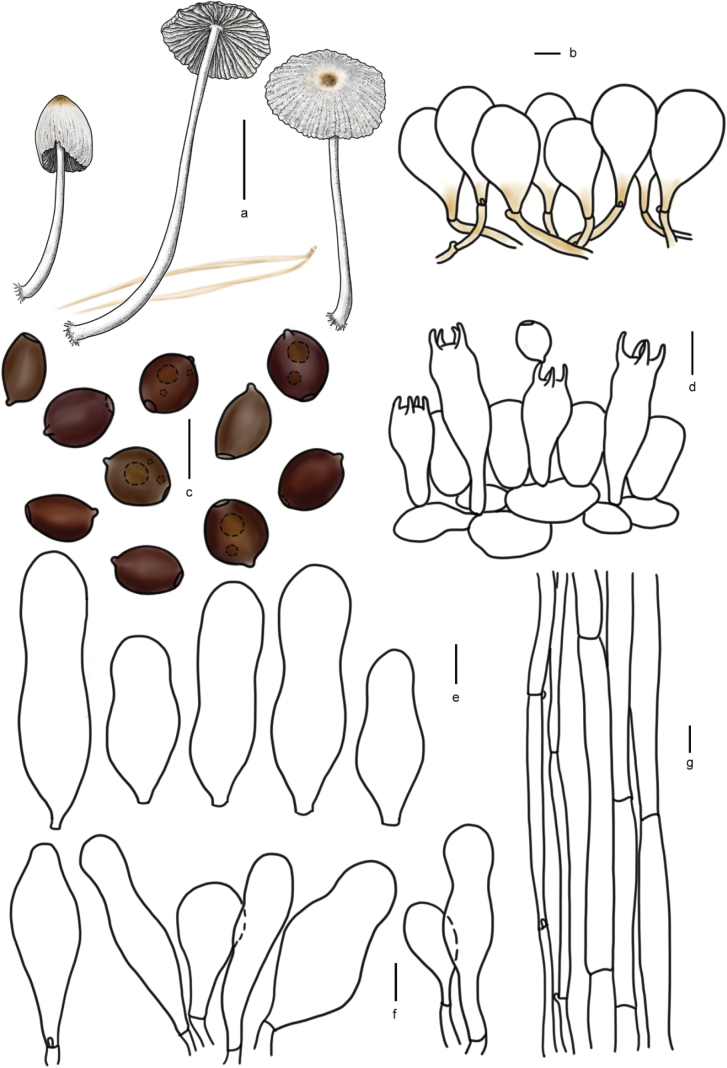
Basidiomata and microscopic structures of *Parasolachowii*: **a** basidiomata (HMJAU60358), **b** pileipellis (HMJAU60358), **c** basidiospores (HMJAU60358, 60374, HMJAU64099), **d** basidia (HMJAU60358), **e** cheilocystidia (HMJAU60358, HMJAU64099), **f** pleurocystidia (HMJAU60358), **g** stipitipellis (HMJAU60358). Scale bars: 1 cm (**a**); 10 μm (**b–g**).

### ﻿ Parasola neoplicatilis

Taxon classificationFungiAgaricalesPsathyrellaceae

T. Bau, L.Y. Zhu & Q.Q. Liu sp. nov.

978B5B39-E4A5-5853-91B0-CCC19486878C

MB846526

Figs 12g–l , 15

#### Diagnosis.

Pileus light yellow-brown at margin and orange-brown in center when young, light gray-brown and with a bright orange-brown center when mature; stipe white to pale gray at upper part, light brown at lower part; basidiospores 7.4–8.1 × 5.8–6.1 × 5.0–5.4 μm, most in ovoid, mitriform, ellipsoid or limoniform in front view, flattened, ellipsoid to narrow ellipsoid in side view, dark olive-brown to almost black; germ pore slightly eccentric; basidia dimorphic, 16–31 × 5–9 μm, 4-spored; cheilocystidia 25–54 × 10–26 μm, abundant, utriform, broad ellipsoid or broad lageniform, sometimes apically mastoid; pleurocystidia 37–100 × 13–27 μm, sublageniform or (sub)cylindrical; pileipellis a hymeniderm at yellow-brown sulcate, mainly made up of ellipsoid or clavate cells, sometimes subglobose or utriform with short pedicels; sclerocystidia absent; some terminal elements of caulopellis hyphae obtuse, attenuated or cystidioid.

#### Etymology.

The epithet “*neoplicatilis*” means that this species has similar morphological characteristics and close genetic relationship with *P.plicatilis*.

#### Type.

**CHINA** • Jiangsu Province, Nanjing City, Purple Mountain (near Wong Ka Wan MTR Station), 32°08'16"N, 118°83'99"E, 137 m a.s.l., on clayed soil under broad-leaved tree, May 13^th^ 2022, Q. Q. Liu, X. Chen, W. Q. Zhu and Y. Huang, HMJAU64087 (HY51302, holotype).

#### Description.

Pileus 4–6 × 6–9 mm when still closed, 11–15 mm when mature, at first mitriform or ellipsoid, finally almost flattened, sometimes with a conspicuous depressed disc at center; dry; light yellow-brown at margin and orange-brown in center when young, light gray at margin and with a bright orange-brown center at age, sometimes with water-soaking texture; sulcate-striate almost up to center. Context extremely thin, brown in center and almost unseen at margin, odor and taste not distinctive. Lamellae crowded, free and remote from stipe by pseudocollarium, 1 mm in wide, L = 31–40, I = 0 or 1, first white to beige, pale brown-gray when expanded; hardly deliquescing with age. Stipe 48–67 × 2 mm, cylindrical, hollow, equal or attenuate towards the apex, slender, white to pale gray at upper part, light brown at lower part, white to cream at base, glabrous or with sparse tiny hairs. Spore print not recorded.

Basidiospores [58, 5, 3] (6.0)7.4–8.1(9.4) × (4.5)5.8–6.1(6.7) × (4.3)5.0–5.4(6.3) μm, Q_1_ = 1.09–1.51, Q_2_ = 1.33–1.73, av. Q_1_ = 1.28, av. Q_2_ = 1.53; most in ovoid, mitriform, ellipsoid or limoniform with apical papilla and convex base in front view, flattened, ellipsoid to narrow ellipsoid in side view; smooth, dark brown to almost black, with yellow-brown oil droplet; inamyloid; germ pore slightly eccentric, 1.3–1.7 μm wide. Basidia dimorphics, 16–31 × 5–9 μm, sterigma 3–6 μm, clavate, hyaline, 4-spored, surrounded with (3)4–7 pseudoparaphyses; subhymenium composed of subglobose, ellipsoid, oblong or cylindrical elements, 9–21 × 4–7 μm. Cheilocystidia 25–54 × 10–26 μm, abundant, utriform, broad ellipsoid or broad lageniform, sometimes apically mastoid, smooth, colorless, thin-walled. Pleurocystidia 37–100 × 13–27 μm, sublageniform or (sub)cylindrical. Lamella trama regular, 5–12 μm wide, hyaline, colorless, thin-walled. Pileipellis a hymeniderm at yellow-brown sulcate, mainly made up of ellipsoid or clavate cells, sometimes subglobose or utriform with short pedicels, 34–59 × 11–23 μm, hyaline, with brown hue at base; other part of pileus with gray hue a cutis, made up of hyaline, colorless to light brown, 4–9 μm; context at pilei center composed of densely interwoven hyphae, thin-walled, hyaline, yellow brown to brown, 7–14 μm wide. Sclerocystidia absent. Caulopellis hyphae parallel, 4–13 μm wide, hyaline, thin-walled, often diverticulate; hyphae of stipe trama 9–20 μm wide, colorless, thin-walled; caulocystidia unseen but some terminal elements of caulopellis hyphae obtuse, attenuated or cystidioid. Clamp connection and pseudoclamp present.

#### Ecology.

Solitary, subfasciculate, or in small groups, grow on clayey soil or moss layer in broad-leaf forest with *Quercusfabri* and *Zelkovaserrata* or under bush of *Ilexcornuta*. Fruiting in May to September. Known from China by specimen study; other possible distributions are Vietnam and United States based on sequences downloaded from GenBank with invalid epithet “*neoplicatilis*”.

#### Other specimens examined.

**CHINA** • Same place with holotype, 132 m a.s.l., May 13^th^ 2022, Q. Q. Liu, X. Chen, W. Q. Zhu and Y. Huang, HMJAU64088 (CX219); • 127 m a.s.l., June 8^th^ 2022, Q. Q. Liu, Z. H. Zhang, J. M. Cheng, X. Chen, W. Q. Zhu, B. F. Wang, Y. Huang and Y. Chen, HMJAU64089 (HY60902); • 132 m a.s.l., June 25^th^ 2022, Q. Q. Liu, Y. Zhang, X. Chen, and W. Q. Zhu, HMJAU64090; • 135 m a.s.l., September 28^th^ 2022, X. Chen, HMJAU60084 (Z22006).

#### Notes.

*Parasolaneoplicatilis*, *Parasolaplicatilis* and *Parasolapapillatospora* together constitute a unique clade in *Parasola*. Morphologically, these three species also share similar characteristics: red-brown hue at pilei center, sordid whitish to pale yellow-brown stipe, resemble lamellae density, ovoid basidiospores, utriform and sublageniform cheilocystidia and subcylindrical pleurocystidia which up to 100 μm (Uljé 2005; Huang 2019; Pošta et al. 2023). The morphological dissimilarity between the sister species, *Parasolaneoplicatilis* and *Parasolapapillatospora*, is minimal, while *Parasolapapillatospora* processes grayer pileus, broader basidiospores in front view (with width 6.2–8.7 μm), longer basidia (15–45 μm in length) which are surrounded by 4–6 pseudoparaphyses (Pošta et al. 2023). Differs from *Parasolaneoplicatilis*, *Parasolaplicatilis* have larger basidiospores which up to 15.3 μm and basidia which is 20–42 × 9–12 μm in size based on the description by Uljé (2005) and our studies of specimens of *Parasolaplicatilis* (HMJAU46397, HMJAU46402, HMJAU46460, HMJAU60359, HMJAU60366 and HMJAU60367). Among the above mentioned three species, *Parasolaplicatilis* is a widespread species in the north temperate zone, while *Parasolapapillatospora* and *Parasolaneoplicatilis* are only known from East Europe and East Asia, respectively.

Macroscopically, *Parasolaglabra* S. Hussain, Afshan, H. Ahmad & Khalid and *Parasolaplicatilis-similis* are similar to *Parasolaneoplicatilis*. However, *Parasolaglabra* is distinguished from *Parasolaneoplicatilis* by its larger basidiospores (14.5–16.5 × 9.5–11.5 × 8.0–10.5 μm) and shorter pleurocystidia (60–75 × 22–38 μm) (Hassain et al. 2018). Compared to *Parasolaneoplicatilis*, pileus of *Parasolaplicatilis-similis* is darker and its basdiospores are mostly in broad ellipsoid to broad hexagon and larger in size (11.8 × 9.7 μm in average) (Szarkándi et al. 2017).

**Figure 15.**
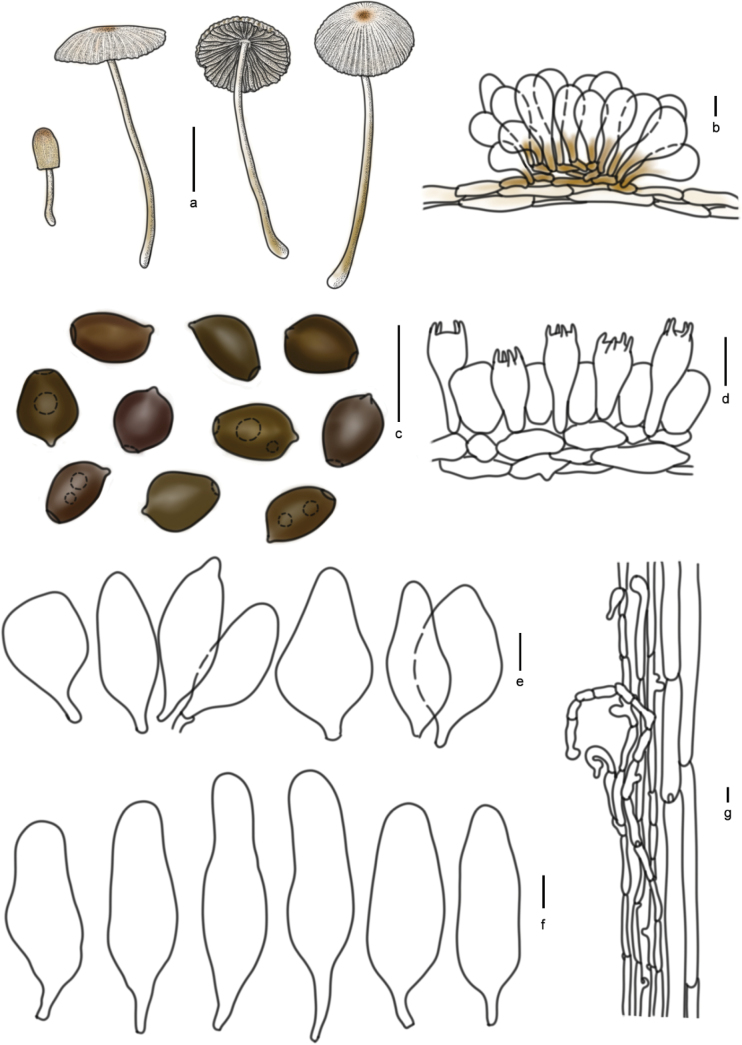
Basidiomata and microscopic structures of *Parasolaneoplicatilis*: **a** basidiomata (HMJAU60084, HMJAU64087, HMJAU67089), **b** pileipellis (HMJAU64087), **c** basidiospores (HMJAU60084, HMJAU64087, HMJAU67089), **d** basidia (HMJAU60084), **e** cheilocystidia (HMJAU60084, HMJAU64087), **f** pleurocystidia (HMJAU60084), **g** stipitipellis (HMJAU60084). Scale bars: 1 cm (**a**); 10 μm (**b–g**).

### ﻿ Parasola megasperma

Taxon classificationFungiAgaricalesPsathyrellaceae

(P.D. Orton) Redhead, Vilgalys & Hopple, in Redhead, Vilgalys, Moncalvo, Johnson & Hopple, Taxon 50(1): 236 (2001).

F316F3B0-1111-5E0C-B365-A71384005FA3

Figs 12o , 16

#### Basionym.

*Coprinusmegaspermus* P.D. Orton, Notes R. bot. Gdn Edinb. 32(1): 141 (1972).

#### Description.

Pileus 8–11 × 6–9 mm when still closed, 15–18 mm when mature, at first semiglobose to mitriform, expanded pileus conical to flattened, mostly with a depressing disc at center; dry; ochreous to dark yellow-brown at center, surrounded with a water-soaked circulus, pale brown-gray at margin, dark yellow-brown at ridge of plication; sulcate-striate up to center. Context extremely thin, almost unseen in membrane part of pileus, cream to sordid yellow, odor and taste not distinctive. Lamellae crowded, free and remote from stipe, with a circular empty space which is visible around the apex of the stipe, 1 mm in wide, L = 35–42, I = 0 or 1, first pale-yellow gray then become gray to purple-gray, or dark gray; hardly deliquescent with age. Stipe 48–71 × 2–3 mm, cylindrical, hollow, equal or slightly attenuate towards the apex, cream and darker at base, glabrous. Spore print not recorded.

Basidiospores [37, 2, 1] (13.1–)14.4–15.1(–16.3) × (9.3–)10.1–10.5(–11.9) × (8.3–)8.8–9.5(–9.7)μm, Q_1_ = 1.28–1.55, Q_2_ = 1.59–1.61, av. Q_1_ = 1.43, av. Q_2_ = 1.60; ellipsoid, with apical papilla and convex base in front view, narrow ellipsoid in side view; smooth, dark brown to almost black; inamyloid; germ pore central, 2.0–2.9 μm wide. Basidia dimorphic, 17–44 × 11–15 μm (in average 32 × 13 μm), sterigma 4–6 μm, clavate, sometimes constricted in middle part, hyaline, 4-spored, surrounded with 5–7 pseudoparaphyses; subhymenium yellow brown, composed of subglobose, ellipsoid, oblong or cylindrical elements. Cheilocystidia 25–55 × 23–34 μm, abundant, subglobose, utriform, ellipsoid or sublageniform, smooth, colorless, thin-walled. Pleurocystidia very rare, when present 49–75 × 22–26 μm, oblong to cylindrical, smooth, colorless, thin-walled. Lamella trama regular, 6–10 μm wide, hyaline, colorless, thin-walled. Pileipellis a hymeniderm, made up of sphaeropedunculate and clavate cells, 30–43 × 13–21 μm, hyaline, with yellow-brown hue at base; pileus trama hyphae densely interwoven, thin-walled or slightly thick-walled, hyaline, yellow-brown, 5–8 μm wide; sclerocystidia absent. Stipipellis hyphae parallel, 4–8 μm wide, hyaline, thin-walled, occasionally with short branches; hyphae of stipe trama 9–20 μm wide, colorless, thin-walled; caulocystidia unseen. Clamp connection present.

#### Ecology.

Solitary, subfasciculate or in small groups, grow on sandy soil or soil with manure in grassy place. Fruiting in July to August. Recorded with certainty from Netherlands, Britain, Poland in Europe (Uljé 2005; Gierczyk et al. 2011; Schafer 2014) and America (Mainland and Hawaii Islands) (Keirle et al. 2004); other reports also from New Zealand (https://www.gbif.org/); only known from Inner Mongolia Autonomous Region in China so far.

#### Specimens examined.

**CHINA** • Inner Mongolia Autonomous Region, Tongliao City, Horqin Left Back Banner, Udantala Forest Farm, July 16^th^ 2022, 42°59'31"N, 122°46'37"E, 207 m a.s.l., T. Bau, W. N. Hou, HMJAU60099 (H2207124); **CHINA** • Inner Mongolia Autonomous Region, Hulunbuir City, New Barag Left Banner, Amugulang Town, Jianggaihuduge, 48°08'41"N, 118°21'05"E, 696 m a.s.l., August 7^th^ 2022, T. Bau and L. Y. Zhu, HMJAU64101 (Z22080716).

#### Notes.

*Parasolamegasperma* could be easily distinguished from other species without sclerocystidia by its large dark ellipsoid basidiospores which are up to 14 μm in average. Moreover, the dark yellow-brown ridge on pileus when mature and the water-soaked circulus around the brown central could also be recognizable characteristics of this species. *Parasolamegasperma* has been often confused with *Parasolaschroeteri* and *Parasolahercules* due to the relatively large basidiospores shared by these three species. However, the shape of basidiospores differ significantly: *Parasolahercules* exhibits basidiospores that are predominantly rounded triangular to weakly 5- or even 7-angular, while those of *Parasolaschroeteri* are consistently rounded triangular (Uljé 2005).

The presence of pleurocystidia in *Parasolamegasperma* varies with specimens. In the specimens examined here, pleurocystidia were very rare and exclusively cylindrical. In contrast, other studies by Keirle et al. (2004), Uljé (2005) and Gierczyk et al. (2011) reported pleurocystidia as ellipsoid to narrow ellipsoid. Notably, this structure was not mentioned in Schafer’s description (2014).

**Figure 16.**
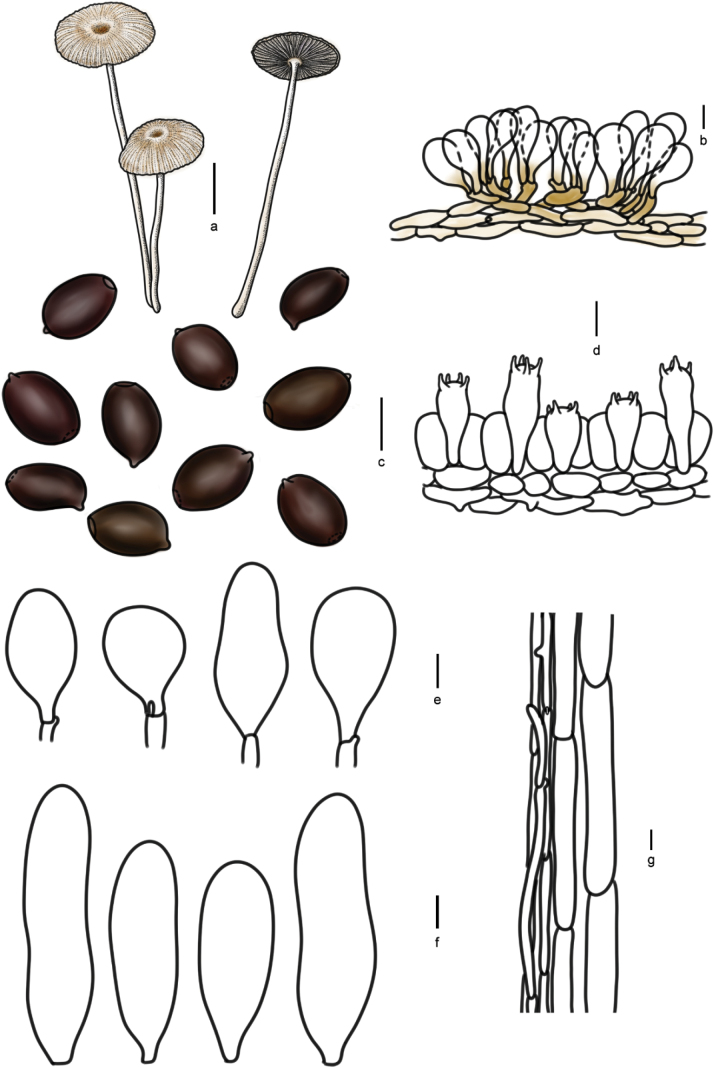
Basidiomata and microscopic structures of *Parasolamegasperma*: **a** basidiomata (HMJAU64101), **b** pileipellis (HMJAU60099), **c** basidiospores (HMJAU60099), **d** basidia (HMJAU60099), **e** cheilocystidia (HMJAU60099), **f** pleurocystidia (HMJAU60099, HMJAU64101), **g** stipitipellis (HMJAU60099). Scale bars: 1 cm (**a**); 10 μm (**b–g**).

### ﻿ Parasola eburnea

Taxon classificationFungiAgaricalesPsathyrellaceae

T. Bau & L.Y. Zhu sp. nov.

056F140A-ABE1-5C4D-B9E3-D90BD099814D

MB846528

Figs 17a–f , 18

#### Diagnosis.

Pileus lacteous, pale gray to gray at margin, sordid yellow to slightly ochreous in center, sulcate-striate almost up to center, ridge of plication fine grained; lamellae free and remote from stipe by pseudocollarium; basidiospores 9.7–10.9 × 10.0–10.4 × 8.4–9.4 μm, rhomboid or mitriform in front view, ellipsoid to narrow ellipsoid in side view, with slightly eccentric or almost central germ pore of 1.8–2.1 μm in diam; sclerocystidia absent.

#### Etymology.

The specific epithet “*eburnea*” refers to the white with slightly sordid yellow color of the basidiocarp.

#### Type.

**CHINA** • Jilin Province, Changchun City, campus of Jilin Agricultural University, 43°80'91"N, 125°40'94"E, 201 m a.s.l., September 15^th^ 2021, L. Y. Zhu, HMJAU60347 (Z21091503, holotype).

#### Description.

Pileus 4–8 × 5–10 mm when still closed, 5–13 mm when mature, at first ovoid or ellipsoid, then paraboloid, finally almost flattened, sometime with a depressed disc at center; dry; sordid yellow to slightly ochreous and in center, sometimes with red-brown hue when dry, lacteous, pale gray or gray at margin, slight brown at center at age; sulcate-striate almost up to center, ridge edge fine-grained. Context extremely thin, almost unseen, odor and taste not distinctive. Lamellae medium crowded, free and remote from stipe by pseudocollarium, 1–2 mm in wide, L = 19–28, I = 1, first white to beige, pale gray to purple-gray when expanded, hardly deliquescent with age. Stipe 15–45 × 1–2 mm, cylindrical, hollow, equal or attenuate towards the apex, white to gray brown, somewhat translucent when moist, glabrous, at base somewhat swollen. Spore print not recorded.

Basidiospores[47, 4, 2] (9.0–)9.7–10.9(–12.1) × (9.0–)10.0–10.4(–11.4) × (7.8–)8.4–9.4(–9.9)μm, Q_1_ = 1.16–1.40, Q_2_ = 1.35–1.83, av. Q_1_ = 1.30, av. Q_2_ = 1.58; rhomboid or mitriform, with apical papilla and convex base in front view, flattened, ellipsoid to narrow ellipsoid in side view; smooth, yellow-brown to dark brown-gray, with yellow-brown oil droplet; inamyloid; germ pore slightly eccentric or almost central, 1.8–2.1 μm wide. Basidia dimorphic, 17–43 × 8–16 μm, sterigma 3–6 μm, clavate, occasionally constricted in middle part, hyaline, 4- or 2-spored, surrounded with 4–6 pseudoparaphyses; subhymenium composed of subglobose, ellipsoid, oblong or cylindrical elements, 8–19 × 6–14 μm. Cheilocystidia 16–52 × 10–22 μm, abundant, subglobose, utriform, ellipsoid, sublageniform or subcylindrical, smooth, colorless, thin-walled. Pleurocystidia 32–82 × 11–32 μm, utriform, ellipsoid, sublageniform or (sub)cylindrical. Lamella trama regular, 3–10 μm wide, hyaline, colorless, thin-walled. Pileipellis a hymeniderm at yellow-brown sulcate, mainly made up of sphaeropedunculate cells, sometimes ellipsoid or utriform with inconspicuous short pedicels, 21–46 × 13–22 μm, hyaline, with brown hue at base in most cases; other part of pileus with gray hue a cutis, made up of hyaline, almost colorless hyphae, 6–8 μm; pileus trama hyphae densely interwoven, thin-walled, hyaline, yellow-brown to brown, 3–8 μm wide. Sclerocystidia absent. Caulopellis hyphae parallel, 3–9 μm wide, hyaline, thin-walled; hyphae of stipe trama 7–15 μm wide, colorless, thin-walled or slightly thick-walled; caulocystidia unseen, occasionally seen specialized terminal elements. Clamp connection present.

#### Ecology.

Solitary, subfasciculate, or in small groups, grow on clayey soil in grassy places or decayed wood chips or humus layer in broad-leaf or coniferous forest, particularly under trees or shrubs. Fruiting in August to September. Only known from northeast China, however, quite common.

#### Other specimens examined.

**CHINA** • Jilin Province, Changchun City, Jilin Agricultural University, 43°80'78"N, 125°41'67"E, 193 m a.s.l., September 11^th^ 2021, L. Y. Zhu, HMJAU60346 (Z21091102), HMJAU60361 (Z21091107), HMJAU60363 (Z21091108); **CHINA** • same place with holotype, September 11^th^ 2021, S. E. Wang, HMJAU60364 (Z21091112); **CHINA** • Jilin Province, Changchun City, Jingyuetan National Forest Park, 43°47'31"N, 125°28'34"E, 206 m a.s.l., August 31^st^ 2021, L. Y. Zhu, HMJAU60362 (Z21083131); **CHINA** • Jilin Province, Changchun City, Nanhu Park, 43°51'14"N, 125°17'57"E, 220 m a.s.l., August 17^th^ 2022, L. Y. Zhu, W. N. Hou, HMJAU60080 (Z22081717), HMJAU60083 (Z22081703); **CHINA** • Jilin Province, Changchun City, Nanhu Park, 43°50'39"N, 125°18'24"E, 221 m a.s.l., August 17^th^ 2022, X. Wang, and L. S. Mu, HMJAU60081 (WX251); **CHINA** • Liaoning Province, Shenyang City, Zhao Mausoleum, ﻿41°84'98"N, 123°42'16"E, 51 m a.s.l., September 21^st^ 2024, Q. Q. Dong and Q. Q. Ye, HMJAU67659 (Z24092110); **CHINA** • Inner Mongolia Autonomous Region, Tongliao City, Horqin Left Back Banner, Daqinggou National Reserve, 42°47'34"N, 112°10'38"E, 247 m a.s.l., August 23^rd^ 2022, T. Bau and L. Y. Zhu, HMJAU60082 (m247).

#### Notes.

The distinguishing characteristics of *Parasolaeburnea* are pileus with relatively lighter color, lamellae free and remote from stipe by pseudocollarium and rhomboid or mitriform basidiospores with slightly eccentric or almost central germ pore. Macroscopically, *Parasolaeburnea* is similar to *Parasolalactea* and *Parasolapseudolactea* for the whitish color of pileus especially when young, however, the latter two species could be easily distinguished from *Parasolaeburnea* by their lamellae adnate or nearly free to apex of stipe and pseudocollarium absent, besides size of basidiospores of *Parasolapseudolactea* is relatively larger, in average 14.0 × 11.3 × 9.7 µm (Smith and Hesler 1946; Nagy et al. 2010a; Hussain et al. 2018). Though sharing a close relationship with *Parasolaeburnea*, *Parasolacrataegi* has yellow brown basidiospores with smaller size (avg 7.4 × 6.5 × 4.8 µm) and almost always grow in close vicinity to *Crataegusmonogyna* (Szarkándi et al. 2017).

**Figure 17.**
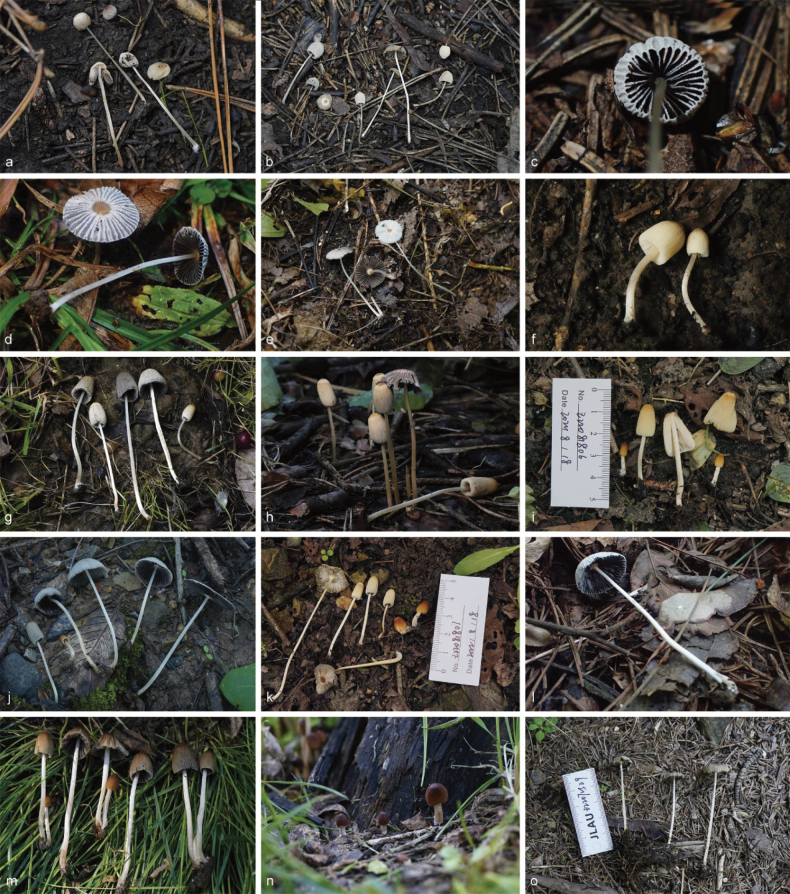
Basidiomata of species in Clade Lactea in sect.Parasola. **a–f***Parasolaeburnea*: **a** HMJAU60080, **b** HMJAU60083, **c** HMJAU60081, **d** HMJAU60347, **e** HMJAU60082, **f** HMJAU60362; **g–l***Parasolaorientolactea*: **g** HMJAU60348, **h** HMJAU60350, **i** HMJAU60085, **j** HMJAU60091, **k** HMJAU60086, **l** HMJAU60089; **m–o***Parasolakuehneri*: **m** HMJAU60343, **n** HMJAU60344, **o** HMJAU60077.

**Figure 18.**
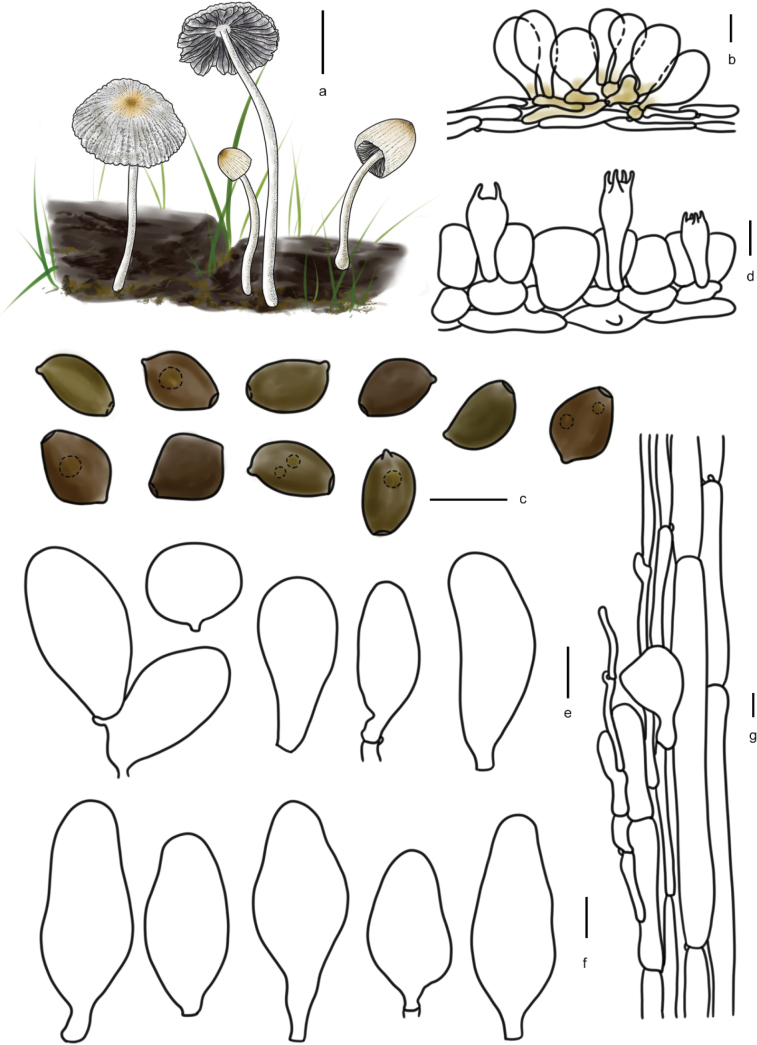
Basidiomata and microscopic structures of *Parasolaeburnea*: **a** basidiomata (HMJAU60347, HMJAU60361, HMJAU60080), **b** pileipellis (HMJAU60347), **c** basidiospores (HMJAU60347, HMJAU60080), **d** basidia (HMJAU60080), **e** cheilocystidia (HMJAU60347, HMJAU60080), **f** pleurocystidia (HMJAU60347, HMJAU60080), **g** stipitipellis (HMJAU60080). Scale bars: 1 cm (**a**); 10 μm (**b–g**).

### ﻿ Parasola orientolactea

Taxon classificationFungiAgaricalesPsathyrellaceae

T. Bau & L.Y. Zhu sp. nov.

97A24335-59CE-5DE8-A7D6-D693612E122B

MB846529

Figs 17g–l , 19

#### Diagnosis.

Pileus lacteous to cream at margin, light brown at center; basidiospores 11.0–11.7 × 11.3–11.8 × 6.9–7.4 μm, with variable shapes, ranging from triangular, pentangular to heart-shape in front view, sometimes with two germ pore, yellow-brown or olive-brown; basidia dimorphic, 2-spored; cheilocystidia 25–47 × 9–22 μm, utriform, ellipsoid, sublageniform, fusiform or clavate; pleurocystidia 40–65 × 13–26 μm, ellipsoid or sublageniform; pileipellis a hymeniderm mixed with clavate and ellipsoid cells; sclerocystidia absent.

#### Etymology.

The specific epithet “*orientolactea*” is combined with “oriento” (eastern) which refers the occurrence of the new species in the Far East and “*lactea*” meaning the close affinity to *Parasolalactea*.

#### Type.

**CHINA** • Jilin Province, Changchun City, National Forest Park, 43°79'65"N, 125°45'51"E, 202 m a.s.l., August 23^rd^ 2021, L. Y. Zhu, HMJAU60350 (Z21082316, holotype).

#### Description.

Pileus 4–7 × 5–9 mm when still closed, 8–13 mm when mature, at first ovoid or ellipsoid, finally almost flattened, often with a depressed disc at center; dry; lacteous to cream at margin and sometimes with brown hue in center when young, pale gray at age, sometimes with water-soaking texture; sulcate-striate almost up to center. Context extremely thin, almost unseen, odor and taste not distinctive. Lamellae medium crowded, free and remote from stipe by pseudocollarium, 1–2 mm in wide, L = 26–39, I = 0 or 1, first white to beige, pale gray to purple-gray when expanded; hardly deliquescent with age. Stipe 35–81 × 1–2 mm, cylindrical, hollow, equal or attenuate towards the apex, white to pale gray, glabrous. Spore print without being recorded.

Basidiospores [64, 5, 3] (10.2–)11.0–11.7(–14.6) × (10.6–)11.3–11.8(–13.7) × (6.0–)6.9–7.4(–8.1) μm, Q_1_ = 0.92–1.07, Q_2_ = 1.45–1.86, av. Q_1_ = 0.98, av. Q_2_ = 1.64; mostly in triangular, pentangular or heart-shape with apical papilla and convex base and occasionally in broad heart-shape or subrectangle when with two germ pore in front view, flattened, ellipsoid to narrow ellipsoid or narrow fabiform in side view; smooth, yellow-brown or olive-brown, with yellow-brown oil droplet; inamyloid; germ pore eccentric, 2.3–4.3 μm wide. Basidia dimorphic, 15–30 × 9–12 μm, sterigma 4–8 μm, clavate, occasionally constricted in middle part, hyaline, 2-spored, surrounded with 4–6 pseudoparaphyses; subhymenium composed of subglobose, ellipsoid, oblong or cylindrical elements, 7–21 × 8–16 μm. Cheilocystidia 25–47 × 9–22 μm, abundant, utriform, ellipsoid, sublageniform, fusiform or clavate, smooth, colorless, thin-walled. Pleurocystidia 40–65 × 13–26 μm, ellipsoid or sublageniform. Lamella trama regular, 4–12 μm wide, hyaline, colorless, thin-walled. Pileipellis a hymeniderm at yellow-brown sulcate, mainly made up of clavate cells, sometimes ellipsoid or utriform with inconspicuous short pedicels, 35–64 × 10–19 μm, hyaline, sometimes with light-brown hue at base; other part of pileus with gray hue a cutis, made up of hyaline, almost colorless hyphae, 4–10 μm; pileus trama hyphae densely interwoven, thin-walled, hyaline, colorless or with pale brown hue, 6–8 μm wide. Sclerocystidia absent. Stipipellis hyphae parallel, 3–6 μm wide, hyaline, thin-walled, often diverticulate; hyphae of stipe trama 8–21 μm wide, colorless, thin-walled; caulocystidia absent. Clamp connection and pseudoclamp present.

#### Ecology.

Solitary, subfasciculate, or in small groups, grow on clayey soil in lawns or decayed wood chips or rotten wood in broad-leaf forest with *Crataegus*, *Ulmus*, *Robinia* and *Quercusmongolica*. August to September. Only known from China, however, quite common.

#### Other specimens examined.

**CHINA** • Same place with holotype, August 31^st^ 2021, L. Y. Zhu, HMJAU60348 (Z21083131); • August 23^rd^ 2021, L. Y. Zhu, HMJAU60349 (Z21082317); • August 26^th^ 2021, L. Y. Zhu, Q. Q. Dong, and Q. Q. Ye, HMJAU60351(Z21082326); • August 24^th^ 2021, L. Y. Zhu, X. Wang, F. Guo, and L. S. Mu, HMJAU60352 (Z21082407); • September 18^th^ 2021, L. Y. Zhu and Q. Q. Ye, HMJAU60091 (Z21091821); • August 18^th^ 2022, L. Y. Zhu and W. N. Hou, HMJAU60085 (Z22081806), HMJAU60086 (Z22081807); **CHINA** • Jilin Province, Changchun City, Changchun Zoological and Botanical Park, 43°51'49"N, 125°19'46"E, 208 m a.s.l., August 23^rd^ 2022, X. Wang, HMJAU60078 (WX379); **CHINA** • Jilin Province, Yanbian Korean Autonomous Prefecture, Antu County, Jianshui River National Wetland Park, 42°24'50"N, 128°07'04"E, 726 m a.s.l., August 1^st^ 2022, H. B. Song and S. E. Wang, HMJAU60088 (S22080103); **CHINA** • Inner Mongolia Autonomous Region, Tongliao City, Horqin Left Back Banner, Daqinggou National Reserve, 42°47'35"N, 112°11'34"E, 223 m a.s.l., August 23^rd^ 2022, T. Bau and L. Y. Zhu, HMJAU60079 (Z2208C3); **CHINA** • Jilin Province, Jilin City, Lake Songhua Scenic Area, 42°72'09"N, 126°70'79"E, 230 m a.s.l., September 20^th^ 2024, X. Y. Zhou, HMJAU67658 (ZXY24092003); **CHINA** • Guizhou Province, Guiyang, Guiyang Forest Park, 26°33'31"N, 106°45'20"E, 1187 m a.s.l., September 28^th^ 2021, T. Bau and L. Y. Zhu, HMJAU60089 (Z21092831); **CHINA** • Guizhou Province, Guiyang City, Changpoling National Forest Park, 26°39'31"N, 106°40'06"E, 1336 m a.s.l., September 29^th^ 2021, T. Bau and L. Y. Zhu, HMJAU60090 (Z21092922).

#### Notes.

Macroscopically, *Parasolaorientolactea* exhibits morphological similarities to *Parasolalactea* and *Parasolapseudolactea*. However, the newly discovered species could be readily distinguished from the latter two species by several characteristics: The presence of 2-spored basidia, yellow-brown or olive-brown basidiospores that sometimes exhibit two germ pores, and the polymorphic cheilocystidia. Consistent with phylogenetic results, our microscopic observation suggested that *Parasolaorientolactea* is the transition species between *Parasolalactea* and *Parasolapseudolactea* (as detailed in Table 4). These three species appear to have relatively restricted geographic distributions, with *Parasolalactea* occurring in Europe, *Parasolapseudolactea* in Central Asia (reported in Pakistan), and *Parasolaorientolactea* in East Asia (specifically China) (Smith and Hesler 1946; Uljé 2005; Nagy et al. 2010a; Hussain et al. 2018). Although BLAST analysis revealed over 98% sequence similarity among these species, both morphological and phylogenetic evidence strongly support their classification into three distinct clades. Notably, these three species share a unique characteristic within the genus *Parasola*—the absence of pigment in their pileipellis cells.

**Figure 19.**
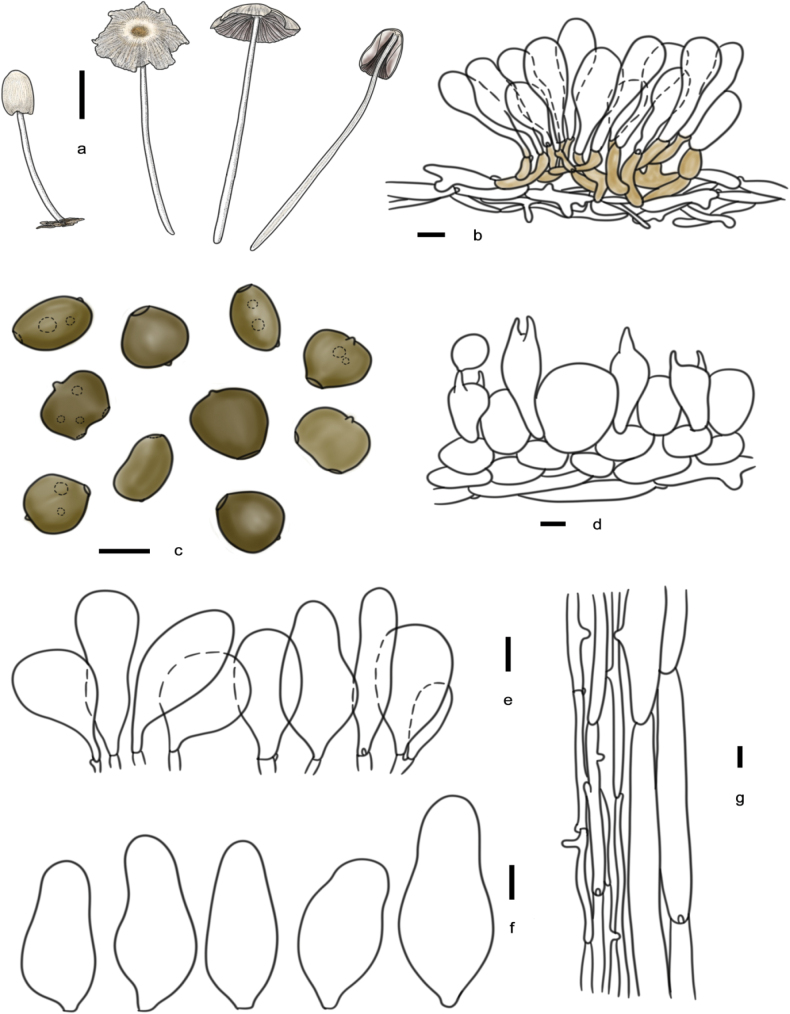
Basidiomata and microscopic structures of *Parasolaorientolactea*: **a** basidiomata (HMJAU60086, HMJAU60350), **b** pileipellis (HMJAU60086, HMJAU60090), **c** basidiospores (HMJAU60086, HMJAU60090, HMJAU60350), **d** basidia (HMJAU60350, HMJAU60349), **e** cheilocystidia (HMJAU60086, HMJAU60350), **f** pleurocystidia (HMJAU60086, HMJAU60350), **g** stipitipellis (HMJAU60350). Scale bars: 1 cm (**a**); 10 μm (**b–g**).

**Table 4.** Characteristics distinguishing *Parasolalactea* and *Parasolaorientolactea* and *Parasolapseudolactea*.

Taxa	Basidiospores		Cheilocystidia	
	size	shape	size	Shape
*Parasolalactea*	8.1–11.8 × 7.1–10.5 × 5.3–7.0 μm	rounded 5-angular and heart-shaped	23–40 × 10–17 μm	utriform or lageniform
*Parasolaorientolactea*	11.0–11.7 × 11.3–11.8 × 6.9–7.4 μm	rounded 3, 5-angular and heart-shaped	25–47 × 9–22 μm	utriform, ellipsoid, sublageniform, fusiform and clavate
*Parasolapseudolactea*	13.5–14.5 × 10.5–12.0 × 9.5-–10.5 μm	rounded 3-angular and heart-shaped	55–70 × 22–29 μm	clavate, broadly clavate to broadly cylindrical

### ﻿ Parasola kuehneri

Taxon classificationFungiAgaricalesPsathyrellaceae

(Uljé & Bas) Redhead, Vilgalys & Hopple, in Redhead, Vilgalys, Moncalvo, Johnson & Hopple, Taxon 50(1): 235 (2001).

DBE2DBAB-ED34-517A-B084-3D473D57D369

Figs 17m–o , 20

#### Basionym.

*Coprinuskuehneri* Uljé & Bas, Persoonia 13(4): 438 (1988).

#### Description.

Pileus 7–9 × 9–14 mm when still closed, up to 19 mm when mature, at first subglobose or ovoid, expanded pileus conical or occasionally flattened; dry; ochreous to red-brown at center, especially when young, usually with shades of gray at margin; at first with distinct longitudinal ridge at center, sulcate-striate up to 3/4 part from margin to center at age, sometimes with cracking edge. Context extremely thin, almost unseen in membrane part of pileus, whitish, young transparent light yellow or slightly brown-gray, brittle, odor and taste not distinctive. Lamellae rather crowded, free, 1–3 mm in wide, L = 46–53, I = 0 or 1, first white to beige, then become pale gray, brown-gray or gray, hardly deliquescent with age. Stipe 46–105 × 2 mm, cylindrical, hollow, almost equal or attenuate towards the apex, sordid white or cream, with brown or ochreous hue at bottom of stipe, glabrous; with white tomentose at base. Spore print not recorded.

Basidiospores (8.5–)9.3–10.5(–12.9) × (7.4–)7.9–8.7(–10.5) × (6.3–)6.5–6.9(–7.5) μm, Q_1_ = 1.15–1.29, Q_2_ = 1.30–1.52, av. Q_1_ = 1.21, av. Q_2_ = 1.44; strongly lentiform, round-angled triangular, rhomboid, ovoid or mitriform, usually with apical papilla and convex base in face view, ellipsoid to narrow ellipsoid in side view; smooth, yellow-brown to brown-black in water and red-brown in 5% KOH, with yellow-brown oil droplet; inamyloid; germ pore eccentric, 1.7–2.8 μm in wide. Basidia dimorphic, 18–36 × 8–10 μm, with brachybasidia which are up to 15 μm in length, sterigma 3–6 μm, clavate, sometimes constricted in middle part, hyaline, 4- or 2-spored, surrounded with 4–7 pseudoparaphyses; subhymenium composed of ellipsoid, oblong or cylindrical elements, 12–28 × 8–17 μm. Cheilocystidia 33–55 × 14–23 μm, abundant, ellipsoid, fusiform or sublageniform, sometimes with a capitate apex, rarely globose, smooth, colorless, thin-walled. Pleurocystidia 46–76 × 17–33 μm, sublageniform or (sub)cylindrical, usually constricted in middle part. Lamella trama regular, 10–19 μm wide, hyaline, colorless, thin-walled. Pileipellis a hymeniderm at yellow-brown sulcate, mainly made up of clavate cells or rarely sphaeropedunculate cells, 25–43 × 13–18 μm, hyaline, with brown hue at base in most cases; other part of pileus with gray hue a cutis, made up of hyaline, almost colorless hyphae, 5–11 μm; pileus trama hyphae densely interwoven, thin-walled, hyaline, yellow-brown to brown, 3–7 μm wide. Sclerocystidia absent. Stipipellis hyphae parallel, 3–7 μm wide, hyaline, thin-walled, often diverticulate; hyphae of stipe trama 11–24 μm wide, colorless, thin-walled; caulocystidia unseen. Clamp-connection and pseudoclamps present.

#### Ecology.

Solitary, fasciculate, or in small groups, usually grow on soil in grassy place (especially near trunks) or soil-covered wood. Fruiting in June to August. Recorded with certainty from Netherlands, France, Germany, British, Poland and Italy in Europe (Uljé and Bas 1988; Uljé 2005), Japan in Asia (treated as Coprinusplicatilisf.microsporus) (Hongo and Aoki 1964); other reports also from North America and Oceania (f) ; only known from Jilin Province in China so far.

#### Specimens examined.

**CHINA** • Jilin Province, Changchun City, campus of Jilin Agricultural University, 43°81'50"N, 125°40'99"E, 147 m a.s.l., June 18^th^ 2021, L. Y. Zhu, HMJAU60343 (Z1); • same place as HMJAU60343, June 19^th^ 2021, L. Y. Zhu, HMJAU60344 (Z2); **CHINA** • Jinlin Province, Jilin City, Jiaohe, Qianjin Forest Farm, 43°54'37"N, 127°38'57"E, 341 m a.s.l., July 22^nd^ 2022, L. Y. Zhu and H. B. Song, HMJAU60078 (Z22072231); **CHINA** • Jilin Province, Jilin City, Jiaohe City, Shansong Ridge, 43°32'57"N, 127°08'07"E, 333 m a.s.l., July 25^th^ 2022, T. Bau, L. Y. Zhu and S. E. Wang, HMJAU60077 (Z22072530).

#### Notes.

*Parasolakuehneri* could be easily distinguished from other species in *Parasola* with its distinct pileus which have red-brown hue and longitudinal ridge at center. It is recorded that *Parasolakuehneri* from Europe have relatively small basidiospores (6.5–10.8 × 5.5–8.2 × 5.0–6.3 μm) which are sometimes heart-shaped and larger cheilocystidia and pleurocystidia which are up to 80 μm and 100 μm, respectively; besides, the European groups prefer to be terrestrial on naked soil that differs from our observations (Uljé and Bas 1988; Uljé 2005; Schafer 2014). *Parasolakuehneri* was recognized as a variety of *Parasolaplicatilis* (Kühner and Josserand 1934; Hongo and Aoki 1964), while the latter have larger basidiospores (10.0–14.5 × 7.0–10.5 × 6.5–8.0 μm) (Uljé 2005).

**Figure 20.**
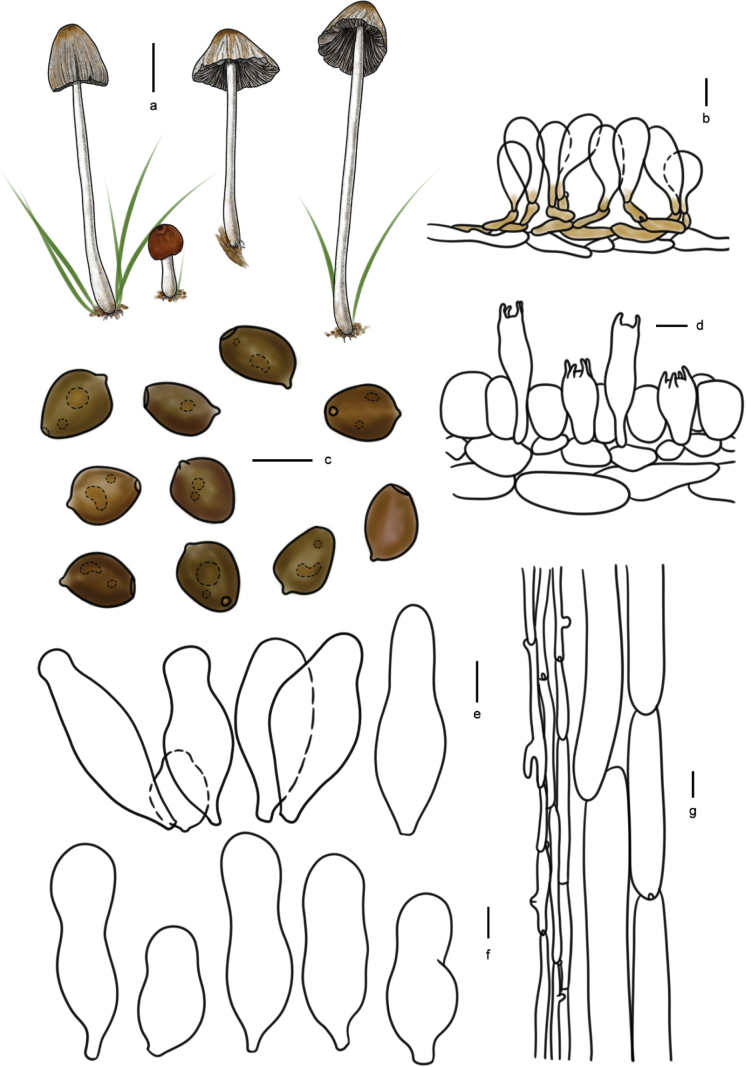
Basidiomata and microscopic structures of *Parasolakuehneri*: **a** basidiomata (HMJAU60343, HMJAU60344); **b** pileipellis (HMJAU60344), **c** basidiospores (HMJAU60344, HMJAU60078), **d** basidia (HMJAU60344), **e** cheilocystidia (HMJAU60343, HMJAU60344), **f** pleurocystidia (HMJAU60342), **g** stipitipellis (HMJAU60343). Scale bars: 1 cm (**a**); 10 μm (**b–g**).

##### ﻿Key to *Parasola*

**Table:** 

1	Basidiomata psathyrelloid; lamellae adnate; basidia monomorphic; caulocystidia even distributed at upper part of stipe	**sect.Conopileae**
–	Basidiomata non-psathyrelloid; lamellae free; basidia dimorphic; caulocystidia accumulate at upper part of stipe	**sect.Parasola**

##### ﻿Key to sect.Conopileae

**Table:** 

1	Pileus without finely sulcate-striate margin	**2**
–	Pileus with finely sulcate-striate margin	**3**
2	Basidiomata small-sized; pileus lacteous to pale brown-gray when mature	***Parasolacrispa***
–	Basidiomata middle-sized; pileus brown to dark brown when mature	***Parasolaconopilea***
3	Specialized pileipellis cells which resemble to pileocystidia present	***Parasolacinnamomescens***
–	Specialized pileipellis cells which resemble to pileocystidia absent	***Parasolapsathyrelloides***

##### ﻿Key to sect.Parasola

**Table:** 

1	Basidiomata secotioid	***Parasolaaporos***
–	Basiodiomata parasoloid	**2**
2	Sclerocystidia present.	**3**
–	Sclerocystidia absent	**7**
3	Average length of basidiospores around 17 μm; germ pore eccentric	***Parasolamalakandensis***
–	Average length of basidiospores less than 15 μm; germ pore central	**4**
4	Pileus in gray hue when mature	**5**
–	Pileus in ochreous or brown hue when mature	**6**
5	Sclerocystidia very thick, often stick together, thickness over 2 μm; pleurocystidia clavate; basidiospores usually with rounded angles	***Parasolasetulosa***
–	Sclerocystidia medium thick, thickness around 1 μm; pleurocystidia subcylindrical or lageniform; basidiospores without rounded angles	***Parasolagrisella***
6	Basidiospores usually present constriction in middle part	***Parasolaconstrictospora***
–	Basidiospores without constriction	***Parasolaauricoma***
7	Basidiocarps always or usually grow on dung	**8**
–	Basidiocarps mainly grow on other substrates	**10**
8	Pileus with purple hue	***Parasolaparvula***
–	Pileus without purple hue	**9**
9	Basidia 2-spored	***Parasolacuniculorum***
–	Basidia 4-spored	***Parasolamisera***
10	Basidiospores longer than 15 µm on average	**11**
–	Basidiospores less than 13 µm on average	**13**
11	Basidiospores ellipsoid, ovoid or oblong in front view, Q > 1.30	**12**
–	Basidiospores rounded triangular in front view, Q < 1.30	***Parasolaschroeteri***
12	Pileus with brown hue at margin	***Parasolamegasperma***
–	Pileus without brown hue at margin	***Parasolaglabra***
13	Grows in winter months in coastal embryonic dunes with scattered halophytic vegetation	***Parasolalitoralis***
–	Grows in other months and habitats	**14**
14	Basidiospores broad ellipsoid, ellipsoid or ovoid, sometimes with rhomboidal outline in face view	**15**
–	Basidiospores round three to seven rounded angles or heart shapes in face view	**19**
15	Basidiocarps relatively small, pileus mean diameter less than 10 mm when expanded	***Parasolatenuissima***
–	Basidiocarps relatively large, pileus mean diameter more than 12 mm when expanded	**16**
16	Average length of basidiospores < 10 µm	**17**
–	Average length of basidiospores between 10 µm to 12 µm	**18**
17	Pileus center cream to pale brown..	***Parasolapapillatospora***
–	Pileus center bright orange-brown	***Parasolaneoplicatilis***
18	Basidiospores mainly in ovoid in face view; pleurocystidia utriform or ellipsoid	***Parasolaplicatilis***
–	Basidiospores mainly in ellipsoid in face view; pleurocystidia narrow lageniform	***Parasolaplicatilis-similis***
19	Grows on grazed grasslands under *Crataegusmonogyna*	***Parasolacrataegi***
–	Grows on other habitats	**20**
20	Pileus slightly yellow-brown, cream or lacteal when young	**21**
–	Pileus strongly orange, ochre to red-brown when young	**24**
21	Basidiospores rhomboid or mitriform in front view	***Parasolaeburnea***
–	Basidiospores in rounded triangle, pentangle or heart-shaped in front view	**22**
22	Basidia 2-spored	***Parasolaorientolactea***
–	Basidia 4-spored	**23**
23	Average length of basidiospores > 13 µm; cheilocystidia clavate to broadly cylindrical	***Parasolapseudolactea***
–	Average length of basidiospores < 11 µm; cheilocystidia utriform, lageniform, ellipsoid or ovoid	***Parasolalactea***
24	Pileus in ochrous or red-brown hue until mature; basidiospores round angled rhomboid, ovoid or mitriform in front view	***Parasolakuehneri***
–	Pileus in gray hue when mature; basidiospores rounded 3-, 5- to 7-angular in front view	**25**
25	Basidiospores mainly rounded 3-angular in face view; cheilocystidia without yellow-gray oil drops	***Parasolahercules***
–	Basidiospores mainly rounded 5- to 7-angular in face view; cheilocystidia with yellow-gray oil drops	**26**
26	Basidiocarps mostly grow in woodland; pileus mainly with depressed disc when completely expanded; stipe white to grayish white, mostly opaque; basidiospores relatively small, 10.7–12.3 × 9.5–10.1 μm in average	***Parasolalilatincta***
–	Basidiocarps mostly grow in lawn; pileus hardly with depressed disc when completely expanded; stipe gray, mostly translucent; basidiospores relatively small, 14.2–14.5 × 12.5 μm in average	***Parasolalilatinctoides***

##### ﻿Plectology of structures in *Parasola*

Pileus of species in sect.Parasola goes through three stages during maturation: (1) the first stage sees the original pileipellis palisaded with stem or clavate cells (Fig. 23a), similar to species of sect.Conopileae (Fig. 22a, b); then hyphae of the secondary pileus extend horizontally and form the groove of the pileus, while the primary pileus remains in the fold’s ridge (Fig. 23b); in the third stage, the pileus is fully extended, the original pileipellis often only retains its pigmented bottom and the secondary hyphae is lighter or colorless (Fig. 23c).

**Figure 21.**
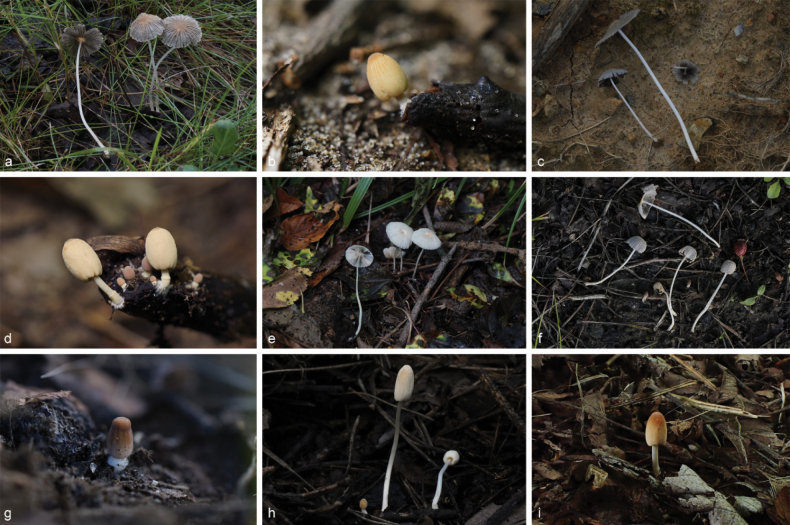
Basidiomata of species in Clade Lilatincta in Sect.Parasola. **a, b***Parasolalilatinctoides*: **a** HMJAU60372, **b** HMJAU60092; **c–f***Parasolalilatincta*: **c** HMJAU60376, **d** HMJAU60375, **e, f** HMJAU60377.

**Figure 22.**
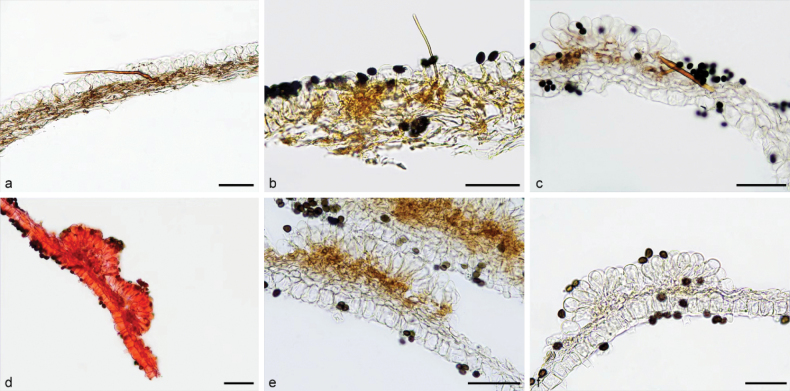
Pileipellis of species of *Parasola*. **a, b**Parasolasect.Conopileae, pileipellis only with original pileipellis in hymeniderm when mature **a***Parasolaconopilea* (HMJAU60342); **b***Parasolacrispa* (HMJAU64096); **c–f**Parasolasect.Parasola, pileipellis with original pileipellis in hymeniderm at ridge part and secondary pileipellis in cutis at groove part when mature **c***Parasolagrisella* (HMJAU64097); **d***Parasolaeburnea* (HMJAU60347); **e***Parasolakuehneri* (HMJAU60343); **f***Parasolaorientolactea* (HMJAU60350). Scale bars: 50 µm (**a–f**).

**Figure 23.**
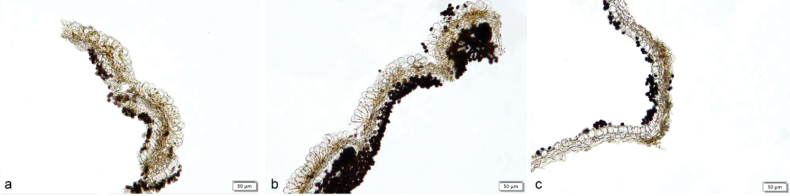
Pileipellis of *Parasolachowii* in different development stage (HMJAU60358): **a** unexpanded stage; **b** initial expanded stage; **c** fully expanded stage. Scale bars:50 µm (**a–c**).

## ﻿Discussion

### ﻿Infrageneric treatment and morphological characteristics of *Parasola*

Our phylogenetic findings corroborate the establishment of sect.Conopileae and sect.Parasola proposed by Wächter and Melzer (2020), based on the monophyly of these two sections. Currently, the primary morphological criteria for distinguishing infrageneric taxa within *Parasola* are the presence or lack of radially sulcate pileus and sclerocystidia (Nagy et al. 2009; Hussain et al. 2018; Wächter and Melzer 2020). However, as previously noted, these criteria do not consistently align with the monophyletic groupings of *Parasola*, as species exhibiting sclerocystidia or a radially grooved pileus are found in both sections. Consequently, alternative morphological characteristics should be considered for grouping criteria.

We conducted a species tree based on multi-gene sequence data, integrating macro- and micro-morphological traits along with ecological information. Our results revealed significant macromorphological differences between these two sections, particularly in the connection type between stipe and lamellae, the shape of pileus, and the presence or absence of cracked plication upon maturity. Species within sect.Conopileae exhibit psathyrelloides basidiomata, characterized by a conical, uncracked pileus at maturity and the lamellae that are narrowly adnated or adnated to the stipe. In terms of microscopic structures, sect.Conopileae is characterized by the absence of a cutis-like secondary pileipellis upon maturation, the monomorphic basidia, and the caulocystidia are distributed along the upper part of the stipe but are not concentrated at the apex. Notably, *Parasolapsathyrelloides*, a member of sect.Conopileae, differs from species in sect.Parasola in that, despite having finely sulcate-striate pileus, its pileus is not cracked and fully expanded when mature (Ganga and Manimohan 2019).

In contrast, most species in sect.Parasola (with the exception for *Parasolaaporos*) have parasoloides basidiomata, featuring a cracked and partially to fully expanded pileus, and a free connection between lamellae and stipe. The absence of lamellae support in the pseudocollarium for the free-type connection is believed to result in the depressed “disc” in the middle portion of pileus in sect.Parasola. Compared to sect.Conopileae, species in sect.Parasola exhibits three distinct features: Microscopic examination reveals three distinct features: the presence of a cutis-like secondary pileipellis upon maturation, the dimorphic basidia (occasionally trimorphic), and the caulocystidia are concentrated at the apex of the stipe. Its development of a secondary pileipellis appears to be directly related to the fully expanded pileus and cracked plication; in addition, the accumulation of terminal caulocystidi may contribute to the free-type stipe-lamellae connection.

**Figure 24.**
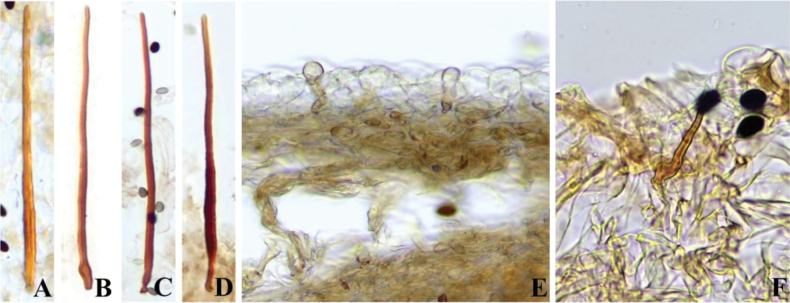
Sclerocystia of species of *Parasola*. **A–D** Xanthochroic reaction of sclerocystidia of *Parasolaconopilea* (HMJAU60365), in 5% citric acid aq., tap water, 5% NaOH aq. and 10% NaOH aq. respectively; it is darker and in red-brown hue in alkaline solution, brighter and in yellow-brown hue in acidic solution; **E** young sclerocystidia of *Parasolaconopilea* (HMJAU60365); **F** clamp connection between sclerocystidia and subpeleipellis hyphae in *Parasolaauricoma* (HMJAU60338).

The presence or absence of sclerocystidia is no longer used as a criterion to distinguish these two sections, and this feature has disappeared during the differentiation of the genus. We further examined whether the growth patten of sclerocystidia is consistent within both sections. Except for sclerocystidia of *Parasolaauricoma* that grow on the secondary pileipeillis hyphae (Fig. 24f), those of other species in genus *Parasola* develop on the subpellis hyphae which is the same as the primary pileipellis cells. Similar to the setae on the hymenium of *Hydnoporiatabacina* (Sowerby) Spirin, Miettinen & K.H. Larss. (referred to as *Hymenochaetetabacina* by Breitenbach and Kränzlin 1986), the sclerocystidia of *Parasola* species exhibit xanthochroic properties, becoming brighter in acidic solution (5% citric acid aq.) and darker in alkaline solution (5% and 10% NaOH aq.) (Fig. 24a–d).

*Parasola*, *Tulosesus* and *Narcissea* D. Wächt. & A. Melzer share similar morphological features and habitats, which can lead to confusion during field work. However, the type of pileipellis (hymeniform in *Parasola*, paraderm in *Tulosesus*, and cutis in *Narcissea*), along with the presence of a veil and thin-walled pileicystidia and caulocystidia, could be useful to distinguish these three genera (Schafer 2010; Wächter and Melzer 2020; Zhu et al. 2022).

### ﻿Phylogenetic problems in *Parasola*

The topology structures of the current phylogenetic trees were like results from former studies (Nagy et al. 2009; Szarkándi et al. 2017; Hussain et al. 2018; Wächter and Melzer 2020), regardless of whether multi-gene or single-gene analysis were employed. While ITS alone or in combination with other genes performs well at resolving relationships at the genus and section levels, it exhibits relatively low discriminatory power among species within sect.Parasola. Adding more gene fragments could enhance and reveal more clades in sect.Parasola and resolve the relationship among Clade Lilatincta and Clade Auticoma.

The high degree of genetic homology among species causes the relatively short average interspecific divergence of *Parasola* (Wächter and Melzer 2020). An obvious example is the extremely short distance between parasoloid-type *Parasolaplicatilis-similis* and secotioid-type *Parasolaaporos*. On the other hand, some widespread species, such as *Parasolaplicatilis* and *Parasolalilatincta*, exhibit relatively large intraspecific distances in our results. For instance, the ITS region similarity between two specimens of *Parasolaplicatilis* (HMJAU60359 and SZMC-NL-0295) is only 95.3%. The long-branch attraction effect caused by extensive intraspecific divergence—may further limit the utility of species discrimination in *Parasola* (Stiller and Hall 1999; Wei et al. 2003; Li et al. 2013; Fagan-Jefferies et al. 2019; Susko and Roger 2021).

### ﻿Suspicious species in *Parasola*

*Parasolaschroeteri* has been a controversial species since its discovery. On the one hand, it is often confused with *Parasolamegasperma* due to its relatively large basidiospores (13.1 × 11.4 μm) (Uljé and Bender 1997); on the other hand, the rounded triangular to ovoid basidiospores of *Parasolaschroeteri* which resemble to other closed related species (*Parasolalilatincta*, *Parasolalilatinctoides* and *Parasolahecules*) often caused misidentification of these species (Hussain et al. 2018; Zhu et al. 2022). According to the evolution trend of basidiospore shape in front view deduced above, *Parasolaschroeteri* should be attributed to Clade Lilatincta rather than Clade Plicatilis for its rounded triangular basidiospores. Based on our results and several other phylogenetic studies (Szarkándi et al. 2017; Wächter and Melzer 2020; Schafer et al. 2022), *Parasolaschroeteri*, *Parasolanudiceps* and *Parasolaochracea* might be the same species because these three species converge into one clade without obvious divergence. Besides, these three species share several morphological common characteristics: (1) the ochre-brown hue at the center of pileus, (2) similar shape and size of basidiospores and (3) the cheilocystidia with similar shapes (Uljé and Bas 1988; Uljé and Bender 1997; Uljé 2005; Nagy et al. 2010a; Szarkándi et al. 2017; Schafer et al. 2022). Considering that the name of Karsten has priority over *Parasolanudiceps* and *Parasolaochracea*, we introduced here the name *Parasolaschroeteri* (P. Karst.) Redhead, Vilgalys & Hopple for *Parasolanudiceps* and *Parasolaochracea* which should be treated as later synonymy of this species. Schafer et al. (2022) also treated these three names as referring to the same species, however they chose *Parasolanudiceps* as the species name for the sequences of holotype specimens of *Parasolaschroeteri* was not available.

In compiling the key of *Parasola*, we noted that the attribution of the four species, *Parasolacystistipitata* P. Voto, *Parasolapallidifusca* P. Voto, *Parasolaelwhaensis* P. Voto and *Parasolabogartii* P. Voto, to this genus was still in doubt as their pileipellis are cellular which do not fit the recognized description of *Parasola* and the central germ pore of these plicate-cap species also reinforce our suspicion; overall, more detailed morphological and phylogenetic studies should be conducted on these species to confirm their taxonomic status (Voto 2019).

### ﻿Formation of pseudocollarium in genus *Parasola*

Currently, there is limited research focusing on the formation of different types of attachment between stipe-lamellae attachment in macrofungi. Based on our observation, the attachment type could serve as a basis for subgenus classification of *Parasola*. Specifically, lamellae are adnate in sect.Conopileae and free in sect.Parasola.

**Figure 25.**
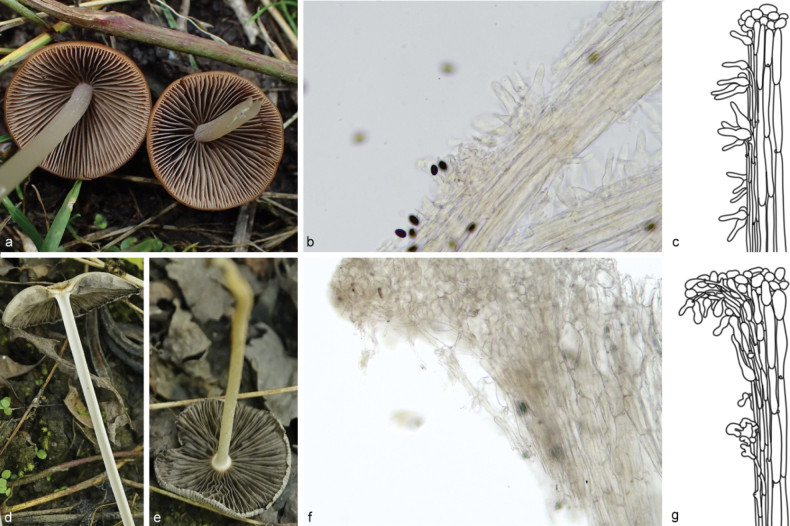
Different connection type of lamellae and stipe of species in sect.Conopileae and sect.Parasola in macro- and micro-scopic **a–c** narrow adnate to adnate in sect.Conopileae (taking *Parasolaconopilea* as an example): **a** the gill-stipe junction is equal in diameter or slightly narrower than the rest of the stipe, the surface of upper part of stipe pubescent; **b** clustered caulocystidia occur on the upper surface of the stipe; **c** illustration of junction part; **d–g** free in sect.Parasola (taking *Parasolagrisella* as an example): **d** the connection part of stipe swelled into inverted trapezoidal shape in lateral view; **e** famellae removed from stipe with pseudocollarium in top view; **f** anatomical section of pseudocollarium in microscopic; **g** illustration of junction part.

In sect.Conopileae, exemplified by *Parasolaconopilea*, caulocystidia are clustered and evenly distributed on the surface of upper stipe (Fig. 25a, c, e). In contrast, in sect.Parasola, such as *Parasolagrisella*, caulocystidia are predominantly grouped at the junction between stipe and pilei context (Fig. 25b, d, f). Focusing on *Parasolagrisella*, the top part of the stripe slightly enlarges into inverted trapezoidal shape macroscopically (Fig. 25b). Microscopically, the terminal hyphae at the top of stipe creep horizontally, accumulating clustered cystidia that forms a pseudocollarium at the stipe-context junction. This phenomenon is common in sect.Parasola and seen in other linages with free lamellae, such as *Agaricusbeijingensis* R.L. Zhao, Z.L. Ling & J.L. Zhou and *Lepiotacristata* (Bolton) P. Kumm in *Agaricaceae* (unpublished). Given the inward growth pattern of lamellae–from the edge of pileus toward the stipe—the accumulating clustered cystidia may act as a physical barrier, preventing the adherence of lamellae and stipe and maintaining a separation between these structures.

It is noteworthy that though the presence of terminal caulocystidia is common in sect.Parasola, it differed from multiform of caulocystidia of *Conocybe* (Hausnecht 2009), the shapes and size of these elements exhibit little variation between species. Thus, terminal cystidia may serve as an intersection classification basis but are not useful for distinguishing species within the same section.

Although the specific function of caulocystidia, especially those on the upper part of stipe, remains unclear, insights can be drawn from carpogenesis of *Parasola*. Taking *Parasolaauricoma* as an example, the stipe could be divided into the upper part and the lower part based on the relative position of the junction to the cap edge (Chi 2013). During development, the upper part of the stipe and the primordial gill grow together within a “cube” covered by a veil. The caulocystidia-like elements on the primary stipe might serve as an “isolated area” keeping gill and stipe growing separately (fig. 5c, d in Chi’s study). However, Chi’s study did not examine the microscopic morphology of these cystidia-like cells on the upper stipe, leaving their identity and developmental trajectory unresolved.

### ﻿Formation of plicate pileus in *Parasola*

To date, only a limited number of studies have focused on the evolution and development of cortical layers of gilled mushroom. Lohwag (1941) proposed a classification of pileipellis structures based on whether the hyphae are repent, or erect and whether they consist of hyphal or cellular elements. The structures include cutis, paraderm, palisoderm, trichoderm and hymeniderm. Vellinga (1988) defined hymeniderm as “A derm made up of non-septate elements originating at the same level”. Although the origin and variation of hymeniderm remain unresolved, insights can be drawn from species with hymeniderm pileipellis and their closely related groups with non-hymeniderm cortex.

One perspective suggests that hymeniderm evolves from cutis or trichoderm, with terminal elements becoming erect and swollen. This is observed in sections in *Entoloma* (Fr.) P. Kumm., where pileipellis transitions from cutis or ixocutis (ixo-: gelatinous) to trichoderm and finally to hymeniderm. The morphogenetic relation between the hymeniderm-like (at center) and the cutis-like (at margin) parts of the pileipellis in *Entolomaappalachianense* Hesler supports this view (Nooderloos 1981, 1988). A similar phenomenon is seen in *Bolbitiaceae*, where basal taxa like *Descolea* Singer exhibit plagiotricholoma (intermediate of cutis and trichoderm with erect but non-swell terminal cells), while core groups such as *Bolbitius* Fr., *Conocybe* Fayod and *Pholiotina* Fayod display hymeniderm (Singer 1951; Hausnechet 2009; Toth et al. 2013). A comparable pattern is also observed in *Pluteus* (Breitenbach and Kränzlin 1995). Another perspective posits that hymeniderm and paraderm could transform into each other, as seen in *Agrocybe* Fayod and *Homophron* (Britzelm.) Örstadius & E. Larss. (Nauta 1987; Yan 2018).

Specific to *Parasola*, the secondary pileipellis composing plitication is repent, i.e., cutis-type. As *Parasola* represents the root taxa of *Psathyrellaceae*, we compared the cortical layer type of the sister group, *Mythicomycetaceae*. Notably, the pileipellis of all known species of *Mythicomycetaceae* is ixocutis, sometimes with a yellow pigment resembling subpileipellis (pileus trama) or secondary pileipellis of *Parasola* species (Vizzini et al. 2019). This suggests that the hymeniderm of *Parasola* may have derived from cutis and could transform to cutis at pleats of species in sect.Parasola. Additionally, the pileus of young basidiomata of *Parasolaconopilea* — a basal species of *Parasola* — exhibits slight stickiness when wet (see in Fig. 6d–f), a trait reminiscent of members of *Mythicomycetaceae*.

In the process of fruiting body maturation of species in sect.Parasola, the proliferation of subpileipellis causes the original pileipellis to crack, forming the ridges of the pleats, and the furrows of the pleats are supported by gills. The pileus matures in much the same way that we open a sunshade, giving more distance between the “bones” (gills) and we speculate that this pattern allows for more efficient basidiospores project. The evolutionary success of species is strongly connected to their reproductive efficiency. Accordingly, traits related to basidiospore generation and dispersal are the top priority of terrestrial mushrooms (Varga 2021). On the one hand, mushrooms form as many gills as possible with a minimal investment of biomass to increase the surface area for spore production; on the other hand, a sufficient integral distance making sure basidiospores are settling downward is necessary as well (Fischer and Money 2010; Iapichino et al. 2021). Mushrooms need to maintain a balance between two strategies and develop one of them as their advantage to successively reproduce (Varga 2021). In coprinoid fungus, *Parasola* and other taxa (*Tulosesus*, *Narcissea* and some species in *Coprinellus* P. Karst) with small basidiomata tend to evolve sulcate-plicate pileus to increase space between mature gills to avoid ejected spores entrapped within hymenium; while the relatively large species, such as *Corprinopsisatramentaria* and *Coprinellusmicaceus* (Bull.) Vilgalys, Hopple & Jacq. Johnson, develop rather crowded gills to produce great numbers of spores. The probable reason for the different choices of these groups is gill spacing becomes inefficient at large radii of pileus (Fischer and Money 2010). Additionally, thick pilei context of large coprinoid species might hamper the formation of pleats, though these taxa usually have large pleurocystidia with function as space makers keeping the gills from colliding (Brefeld 1877; Wettstein 1887; Buller 1910).

*Parasola* is thought to be monovelangiocarpic defined by Reijnder (1963) as its hymenial rudiment is covered only by a universal veil based on previous research about carpogenesis of *Parasolaauricoma* by our team (Chi 2013); however, according to a series of morphological studies of this genus, veil was never observed in mature basidiocarps. This raises several questions: how does the original veil change in development and what exactly is the carpogenesis type of *Parasola*? One possible answer is that the veil does disappear after fruiting body matures; in other words, the mature basidiome is naked. In this condition, the ontogeny process could be defined as hypovelaniocarpy based on Reijnders’ terminology (Reijnders 1963; Clémençon et al. 2012). Another possible answer is that the veil does present when mature, in that case, *Parasola* is monovelandiocarpy like other coprinoid fungi without species of *Coprinus*. Here, we found the erect attenuated terminal hyphae on the suprapellis of stipe in aged fruiting bodies of *Parasolaconstrictospora* and *Parasolagrisella*. Moreover, similar hyphae were also found in young basidiomata of *Parasola* which could match up the veil-like structure in the middle part of the stipe macromorphologically. Interestingly, semblable phenomenon was found in young basidiocap of *Parasolaconiculorum* and *Parasolalilatincta* in Schafer’s study (2014) of British *Parasola* species (in fig. 4a and fig. 8b of his study, respectively). Whether these stipepellis elements are veil residue is still unresolved and requires more ontogeny and histology research about this lineage.

## Supplementary Material

XML Treatment for Parasola

XML Treatment for Parasola sect. Conopileae

XML Treatment for Parasola crispa

XML Treatment for Parasola sect. Parasola

XML Treatment for Parasola constrictospora

XML Treatment for Parasola grisella

XML Treatment for Parasola malakandensis

XML Treatment for Parasola tenuissima

XML Treatment for Parasola chowii

XML Treatment for Parasola neoplicatilis

XML Treatment for Parasola megasperma

XML Treatment for Parasola eburnea

XML Treatment for Parasola orientolactea

XML Treatment for Parasola kuehneri

## ﻿Additional information

### Adherence to national and international regulations

All the fungal strains used in this study have been legally obtained, respecting the Convention on Biological Diversity (Rio Convention).

### Author ORCIDs

Li Yang Zhu https://orcid.org/0000-0001-9376-7507

Tolgor Bau https://orcid.org/0000-0003-2461-9345

### Data availability

The combined alignments used for the phylogenetic analyses are deposited in Zenodo (https://doi.org/10.5281/zenodo.14287746). The new-generated sequences are deposited in GenBank.
